# Modelling the spatial heterogeneity and molecular correlates of lymphocytic infiltration in triple-negative breast cancer

**DOI:** 10.1098/rsif.2014.1153

**Published:** 2015-02-06

**Authors:** Yinyin Yuan

**Affiliations:** Division of Molecular Pathology, Centre for Evolution and Cancer and Centre for Molecular Pathology, The Institute of Cancer Research, London SM2 5NG, UK

**Keywords:** image analysis, immune response, tumour microenvironment, statistical modelling

## Abstract

Lymphocytic infiltration is associated with a favourable prognosis and predicts response to chemotherapy in many cancer types, including the aggressive triple-negative breast cancer (TNBC). However, it is not well understood owing to the high levels of spatial heterogeneity within tumours, which is difficult to analyse by traditional pathological assessment. This paper describes an unbiased methodology to statistically model the spatial distribution of lymphocytes among tumour cells based on automated analysis of haematoxylin-and-eosin-stained whole-tumour section images, which is applied to two independent TNBC cohorts of 181 patients with matched microarray gene expression data. The novelty of the proposed methodology is the fusion of image analysis and statistical modelling for an integrative understanding of intratumour heterogeneity of lymphocytic infiltration. Using this methodology, a quantitative measure of intratumour lymphocyte ratio is developed and found to be significantly associated with disease-specific survival in both TNBC cohorts independent to standard clinical parameters. The proposed image-based measure compares favourably to a number of gene expression signatures of immune infiltration. In addition, heterogeneous immune infiltration at the morphological level is reflected at the molecular scale and correlated with increased expression of *CTLA4*, the target of ipilimumab. Taken together, these results support the fusion of high-throughput image analysis and statistical modelling to offer reproducible and robust biomarkers for the objective identification of patients with poor prognosis and treatment options.

## 1. Introduction

Accumulating evidence supports the clinical significance of immune response in many cancer types [1–3]. Consistent studies have reported associations between immune activity and disease outcome as well as treatment response [1–6]. Furthermore, data from clinical trials have demonstrated the potential of immunotherapies in certain types of cancer [7,8]. This is perhaps best exemplified in late-stage melanoma where recent clinical trials have shown an increased survival advantage in patients receiving the *CTLA4* monoclonal antibody ipilimumab [7]. This has led to the development of more standardized methods of characterizing tumour immune infiltrate in cancers, such as the ‘immunescore’ that aims to quantify the *in situ* immune infiltrate in addition to standardized clinical parameters to aid prognostication and patient selection for immunotherapy in colorectal cancers [9].

However, to facilitate the standardization and reproducibility of scoring immune infiltration, objective approaches are urgently needed [9]. Furthermore, such approaches need to account for the complexity of immune infiltration in tumours. Abundance, spatial heterogeneity and type of immune cells are the key parameters of immune infiltration [9,10]. For example, the spatial locations of immune cells have been shown to be useful in predicting the prognosis of colorectal cancer [1]. Indeed, the pathological ‘immunscore’ is based on the numeration of two lymphocyte populations (*CD8*+ and *CD45RO*+ cells), both in the core of the tumour and in the invasive margin that maximizes the prognostic power [9]. Similarly, large-scale studies of breast cancer have demonstrated that pathological assessment of tumour-infiltrating lymphocytes, based on haematoxylin and eosin (H&E)-stained core biopsies, is a significant predictor for response to neoadjuvant chemotherapy in 1058 breast cancer samples [2]. Recently, a prospective study demonstrated that in *HER2*-negative breast cancer stromal lymphocytes can be an independent predictor of response to neoadjuvant chemotherapy [11]. Thus, the spatial organization of lymphocytic infiltration in the context of nearby cancer cells is an important clinicopathological feature of tumours.

Specifically for triple-negative breast cancer (TNBC), an active immune response has been associated with favourable prognosis [2–4]. A large-scale immunohistochemistry study of 3400 breast cancer samples showed that TNBC is the only subtype of breast cancer to demonstrate a significant link between *CD8*-positive immune cells and a good prognosis [4]. Assessment of lymphocytic infiltration based on whole-tumour H&E sections has been associated with favourable outcome in 256 patients after anthracycline-based chemotherapy [3]. A recent prospective study showed that the presence of tumour-infiltrating lymphocytes in residual tumours after neoadjuvant chemotherapy is predictive of good prognosis in TNBC [12]. Given the lack of targeted molecular treatment of TNBC, this may suggest new therapeutic opportunities for this aggressive tumour type [8]. For instance, accumulating data support that anthracyclines mediate their action through activation of *CD8*+ T-cell responses, hence combination with certain immunotherapies could be especially effective for TNBC [8].

Despite these advances in our understanding of the importance of immune infiltration for TNBC, there is a lack of reproducible approaches to objectively assess immune infiltration based on pathological sections. Previously, we have demonstrated that automated image analysis can be used to objectively and accurately score heterogeneous cell types in breast cancer H&E sections [13]. In particular, using this methodology, we have shown that a measure of the proportion of lymphocytes among all cell types is predictive of disease-specific survival in oestrogen receptor (ER)-negative breast tumours [13]. In parallel, other groups have demonstrated the efficiency and accuracy of image analysis to identify immune cells in pathological samples [14,15]. Specifically, Basavanhally *et al*. [15] were among the first to demonstrate how an automated approach combining image analysis with machine learning can be used to characterize lymphocytes and their spatial distribution in 12 *HER2*+ breast cancer patients.

Given the clinical importance of the immune cell infiltration in TNBC, the aims of this study are to (i) develop an unbiased statistical based computational model of immune infiltrate using automated image analysis from H&E tumour sections, (ii) use this to assess the spatial heterogeneity and clinical implications of immune infiltration in TNBC in two independent TNBC cohorts from the METABRIC study [16] and (iii) assess the molecular heterogeneity of immune cell infiltrate through integration with gene expression molecular profiling data.

## 2. Results

### 2.1. Statistical modelling of the spatial heterogeneity of immune infiltration

**Figure 1.**
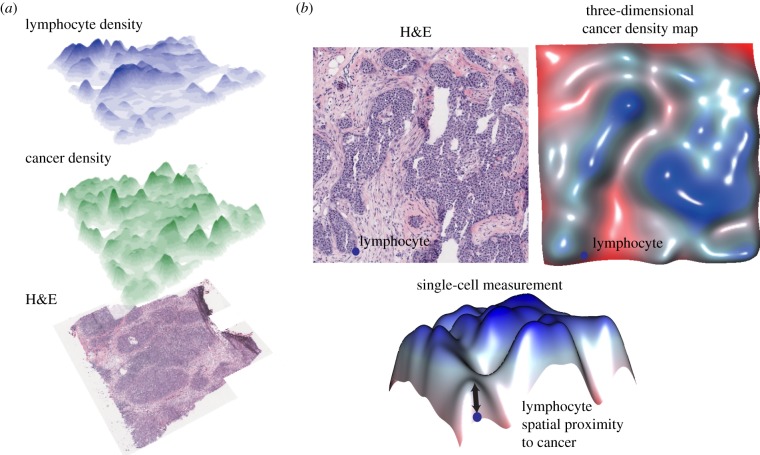
Intratumour heterogeneity of cancer cell and lymphocyte distributions. (*a*) Three-dimensional landscapes illustrating the spatial heterogeneity of cancer cells and lymphocytes in an H&E breast whole-tumour section. The height of the hills in the three-dimensional landscape represents the density of cells. (*b*) Combined analysis of the spatial distribution of cancer and lymphocytes can lead to quantification of lymphocytic infiltration. Shown are a small H&E image and the corresponding three-dimensional cancer density map, which facilitate the measurement of spatial proximity to cancer for every single lymphocyte in the image. (Online version in colour.)

Our image analysis tool identifies cancer, lymphocytes and stromal cells encompassing fibroblasts and endothelial cells based on their nuclear morphologies in H&E whole-tumour section slides [13]. The main component of this tool is a classifier trained by pathologists over randomly selected tumour regions and validated in 564 breast tumours with 90% accuracy [13]. As a result of image analysis, the types and spatial locations of on average 110 000 cells are recorded in every breast tumour section. Thus, this fully automated tool enables the mapping of spatial distributions of all cancer cells and lymphocytes within a tumour section, which can be subsequently visualized as a three-dimensional landscape (figure 1*a*). The spatial relationships of immune and cancer cells are then analysed with a statistical pipeline exemplified in figure 1*b*. First, to globally profile the spatial distribution of cancer cells, the cancer cell density was quantified using a kernel estimate (Methods). Intuitively, this builds a ‘cancer landscape’ where hills indicate tumour regions densely populated with cancer cells. The height of a hill thus correlates with cancer density at a specific location in the tumour (figure 1*b*). Second, for every lymphocyte, its spatial proximity to cancer can be directly quantified with the cancer density landscape at its specific location. Thus, a quantitative measurement of the spatial proximity to tumour cells can be efficiently obtained for every lymphocyte (figure 1*b*).

**Figure 2.**
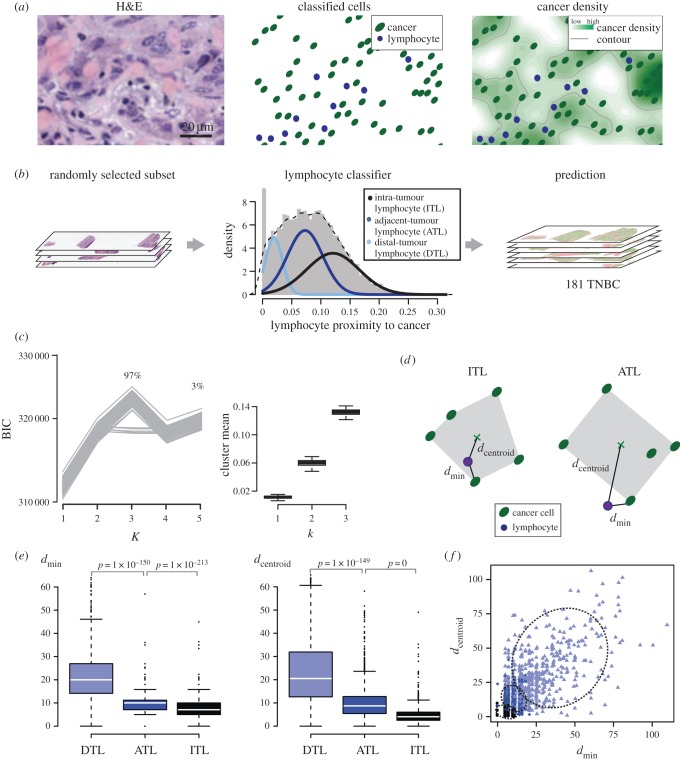
Quantifying the intratumour heterogeneity of lymphocytic infiltration. (*a*) Schematic depiction of the computational pipeline exemplified with a small region of a breast cancer H&E section: H&E image; classified cells using automated image analysis; a map of cancer density based on image analysis result to quantify cancer–immune spatial relationships. (*b*) Discovery of three categories of lymphocytes with unsupervised clustering based on the spatial proximities of lymphocytes to cancer in a subset of TNBC samples. These data were then used to predict the categories of all lymphocytes in all TNBC samples. (*c*) Optimal number of cluster *K* as suggested by BIC over 200 random sampling is 3 in 97% of the repeats and 5 in 3% of the repeats. BIC curves for the 200 sampling are shown on the left, and boxplot showing cluster means for *K* = 3 solutions in 200 sampling on the right. (*d*) Illustration of the distance to the nearest cancer cell *d*_min_ and the distance to the centroid of convex hull region formed by five nearby cancer cells *d*_centroid_. (*e*) Boxplots to show the differences among lymphocyte classes in terms of *d*_min_ and *d*_centroid_ (*p*-values by *t*-test). (*f*) Scatter plot showing *d*_min_ and *d*_centroid_ for 1000 randomly selected lymphocytes, coloured based on the three classes; dashed ellipses showing three clusters fitted to *d*_min_ and *d*_centroid_.

Using this approach, we quantified the spatial proximity to cancer for every lymphocyte in 181 TNBC samples in the METABRIC study (Methods and figure 2*a*). In principle, lymphocytes that differ in their spatial positioning to cancer can be differentiated based on these quantitative spatial measurements. We thus asked whether data-driven clustering methods based on normal distribution can be used to differentiate different classes of lymphocytes, because cell spatial distribution is a naturally emerged pattern. Unsupervised Gaussian mixture model clustering [17] was employed to identify lymphocyte clusters based on their spatial proximity to cancer using a training set of 100 000 randomly sampled lymphocytes (figure 2*b* and Methods). Subsequently, a three-cluster solution that identifies three classes of lymphocytes was considered the optimal by the Bayesian information criterion (BIC) [18] (figure 2*b*). This three-class solution is the optimal 97% of the time upon 200 repeated sampling, whereas the five-class solution was considered optimal 3% of the time (Methods and figure 2*c*). In addition, the cluster structure of the three-class solution was stable (median of cluster mean: 0.011, 0.06, 0.13; standard deviation (s.d.): 0.002, 0.0047, 0.0045; figure 2*c*), indicating that the same clusters were identified in each random sampling. We named the three classes of lymphocytes as intratumour lymphocyte (ITL), adjacent-tumour lymphocyte (ATL) and distal-tumour lymphocyte (DTL). Subsequently, a classifier was trained based on the lymphocyte classes to predict the types of lymphocytes in all TNBC samples (Methods).

**Figure 3.**
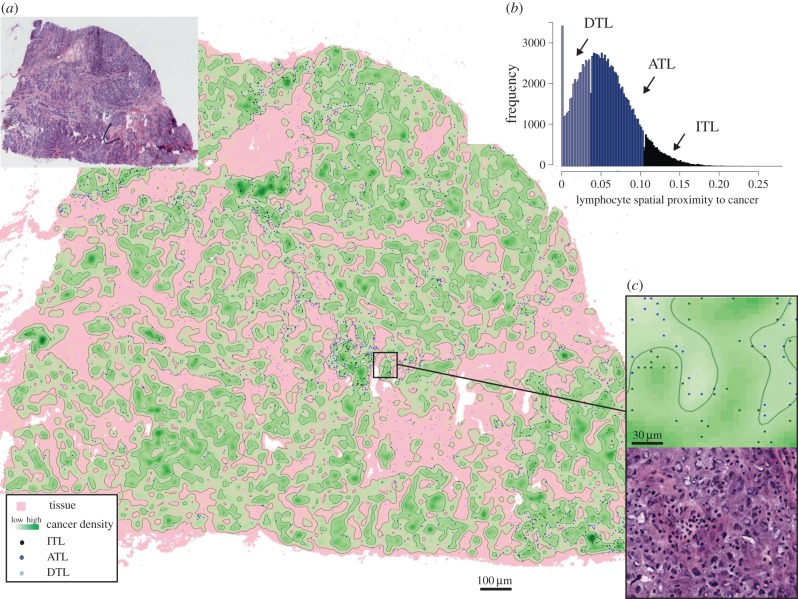
A representative example illustrating three classes of lymphocytes in cancer density map of a tumour (middle section). (*a*) Density map of cancer and the spatial distribution of three classes of lymphocytes (spatial points coloured according to the classes). Black contour lines denote cut-off thresholds for the three classes of lymphocytes according to cancer density. (*b*) Histogram showing the three types of lymphocytes in this sample. (*c*) A higher-resolution image of a region in this sample; colour codes follow (*a*).

To examine differences among the newly proposed lymphocyte classes, we derived additional measures based on direct physical distances. First, for each lymphocyte, its distance to the nearest cancer cell can be quantified (*d*_min_, Methods and figure 2*d*). We found that ITLs have a median distance of 7 μm (interquartile range 5–10) to the nearest cancer cell, whereas it is 10 μm (7–11) for ATLs, and 20 μm (14–26) for DTLs (figure 2*e*). The overlap in distance to nearest cancer cell between ITLs and ATLs suggests that this measure is not the fundamental difference between the two classes. Because our kernel density measure based on which the lymphocyte classes were derived is essentially spatial smoothing, we postulated that the spatial arrangement of cancer cells surrounding lymphocytes differs between ATLs and ITLs. To measure spatial arrangement, we examined the convex hull region formed by five nearest cancer cells, which is the smallest region that covers these cells (figure 2*d* and Methods). If a lymphocyte is surrounded by cancer cells, it should fall into the convex hull region formed by nearby cancer cells and has a small distance to the centroid of this region (figure 2*d*, left). By contrast, if nearby cancer cells are situated to one side of a lymphocyte, the distance between the lymphocyte and the centroid of the cancer convex hull region is likely to be large (figure 2*d*, right). Thus, we used the distance between a lymphocyte and the centroid of the cancer convex hull region as a quantitative measure of the spatial arrangement of cancer cells surrounding a lymphocyte (*d*_centroid_). Three lymphocyte classes displayed significant differences in *d*_centroid_ with median *d*_centroid_ 3.6 μm (2.2–5.1), 7.2 μm (4.5–10.6), 17.7 μm (11.0–26.6) for ITLs, ATLs and DTLs, respectively (figure 2*e*). Therefore, *d*_min_ and *d*_centroid_ together better define and aid our interpretation of the lymphocyte classes (figure 2*f*). Taken together, our data demonstrated that the proposed kernel-based measure of spatial proximity to cancer can effectively account for spatial proximity and surroundings, and that the three lymphocyte classes differ not only in the distance to the nearest cancer cell, but also in the ways nearby cancer cells are arranged. A representative case showing spatial distribution of lymphocytes in these three classes is illustrated (figure 3*a,b*). For instance, the ITL locations can be observed to be within regions densely populated with cancer cells (figure 3*c*).

**Figure 4.**
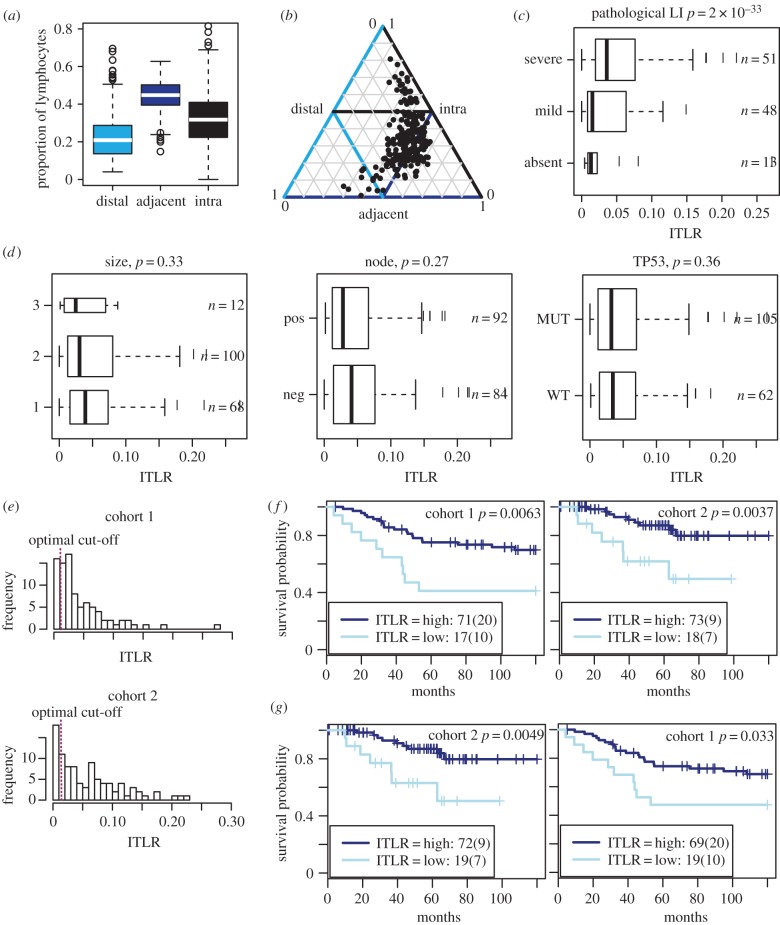
Association between ITLR and clinical parameters of TNBC. (*a*) Proportions of three classes of lymphocytes in 181 TNBCs. (*b*) Triangle plot to show the lymphocyte composition for each tumour (each black dot represents a tumour; thin lines mark the 50% of corresponding axis). (*c*) Boxplot to show correlation between pathological scores and ITLR; *p*-value from JT-test; *n* = patient number is each group; whiskers extend to 1.5 interquantile range. (*d*) Association between ITLR and tumour size, node status and *TP53* mutations; whiskers extend to 1.5 interquantile range. (*e*) Distribution of ITLR in two cohorts with optimal cut-offs marked as dashed red lines. (*f*) Kaplan–Meier curves to illustrate the disease-specific survival probabilities of patient groups in two TNBC cohorts stratified by ITLR using the cut-off selected in cohort 1. Numbers in the legend show the number of patients in each group and numbers in the bracket show the number of disease-specific deaths. (*g*) Using cohort 2 as the discovery cohort and cohort 1 as the validation cohort yielded similar optimal cut-off. (Online version in colour.)

In the 181 TNBC samples, there are overall more ATLs than the other two types of lymphocytes (on average 47% ATLs, 32% ITLs and 21% DTLs; figure 4*a*). The changes in abundance of these three classes in 181 samples can be observed in a triangle plot (figure 4*b*). When the proportion of ITLs is low (0–20%), there are, in general, more DTLs (40–60%) than ATLs (30–50%). As the amount of ITLs increases (20–50%), ATLs also increase (40–60%), whereas DTLs decrease (10–40%). When there is a large amount of ITLs (more than 50%), there is still a substantial amount of ATLs (20–40%) with very few DTLs (less than 10%). To summarize the degree of lymphocytic infiltration for a given tumour, we first calculated the ratio between the number of ITLs and the number of cancer cells (ITLR; Methods). In the 181 TNBC samples, a significant association was observed between ITLR and pathological assessment of lymphocytic infiltration of the tumours in categories of absent, mild and severe (*p* = 2 × 10^−33^, figure 4*c*). In terms of other clinical parameters, there was no correlation between ITLR and tumour size, node status and *TP53* mutation status (figure 4*d*). Tumour grade was not considered, because 87% of the TNBC samples are grade 3 tumours. Taken together, these data support ITLR's validity as a measurement of lymphocytic infiltration and its potential value in addition to known clinical parameters for TNBC.

### 2.2. ITLR is a statistical measure of lymphocytic infiltration and an independent predictor of disease-specific survival in two triple-negative breast cancer cohorts

To investigate the clinical significance of ITLR, we analysed disease-specific survival as a function of ITLR. The TNBC samples can be divided into two independent cohorts based on contributing hospitals (Methods, *n* = 89 and *n* = 92). To dichotomize the continuous ITLR, the optimal cut-off was selected to have the best prognostic value in cohort 1 as the discovery cohort (Methods). The best cut-off was selected to be 0.011, and 20% of the patients have ITLR lower than this cut-off (figure 4*e*). These patients have significantly worse disease-specific survival compared with patients with higher ITLR in cohort 1 (log-rank test *p* = 0.0063, hazard ratio HR = 0.36, 95% confidence interval, CI = 0.17–0.77; table 1 and figure 4*f*). This observation was verified in the validation cohort 2 (*p* = 0.0037, HR = 0.25, CI = 0.09–0.69; figure 4*f*). Good patient stratification was observed upon repeated analysis with cohort 2 as the discovery and cohort 1 as the validation cohort (figure 4*g*). The same tests were performed for the ratio of ATLs and DTLs to cancer cells (ATLR and DTLR), but neither showed a significant correlation with disease-specific survival (discovery and validation cohort: ATLR *p* = 0.064 and 0.75; DTLR *p* = 0.43 and 0.25; electronic supplementary material, figures S1–S2). We subsequently focused on ITLR. ITLR-high TNBC patients have a survival probability of 80% 5 years from diagnosis versus 49% for ITLR-low patients (Kaplan–Meier survival estimates, two cohorts combined).

**Table 1.** Univariate and multivariate Cox regression results for ITLR and other signatures in two TNBC cohorts. Uni, univariate Cox regression; HR, hazard ratio; CI, lower and higher 95% confidence interval; Conc, concordance; 0(0-Inf): where the Cox model failed to converge. *p*-values that pass the significant threshold of 0.05 are shown in italics.

		cohort 1			cohort 2		
		HR (CI)	*p*	conc.	HR (CI)	*p*	conc.
*ITLR*	uni.	0.36(0.17–0.77)	*0.0063*	0.601	0.25(0.09–0.69)	*0.0037*	0.659
	ITLR	0.32(0.15–0.7)	*0.0042*	0.668	0.15(0.05–0.43)	*0.00051*	0.76
	node	0.63(0.29–1.4)	0.26		4.93(1.61–15.08)	*0.0052*	
	size	2.62(1.27–5.41)	*0.0092*		2.07(0.9–4.74)	0.087	
*Lym*	uni.	0.47(0.21–1.02)	0.051	0.574	0.41(0.12–1.43)	0.15	0.575
	lym	0.48(0.22–1.05)	0.066	0.656	0.23(0.05–1.02)	0.053	0.735
	node	0.69(0.32–1.5)	0.35		4.65(1.46–14.81)	*0.0092*	
	size	2.35(1.16–4.77)	*0.018*		1.66(0.65–4.25)	0.29	
*Calabro*	uni.	0.25(0.12–0.52)	*5.2 × 10^−5^*	0.66	0.5(0.18–1.39)	0.18	0.587
	calabro	0.27(0.13–0.56)	*3.8 × 10^−4^*	0.703	0.41(0.14–1.19)	0.1	0.744
	node	0.75(0.35–1.6)	0.45		4.57(1.45–14.37)	*0.0093*	
	size	2.26(1.07–4.76)	*0.032*		1.91(0.82–4.46)	0.13	
*Ascierto*	uni.	0.34(0.15–0.77)	*0.0066*	0.621	1.23(0.4–3.83)	0.72	0.51
	ascierto	0.39(0.17–0.88)	*0.024*	0.671	1.18(0.37–3.72)	0.78	0.735
	node	0.85(0.39–1.84)	0.68		3.6(1.21–10.7)	*0.021*	
	size	2.06(1.02–4.16)	*0.044*		2.16(0.86–5.45)	0.1	
*IL8*	uni.	3.09(1.46–6.51)	*0.0018*	0.615	0(0-Inf)	*0.0099*	0.645
	*IL8*	2.79(1.32–5.92)	*0.0073*	0.679	0(0-Inf)	1	0.808
	node	0.81(0.37–1.75)	0.59		3.14(1.06–9.34)	*0.039*	
	size	2.23(1.08–4.63)	*0.031*		1.75(0.71–4.28)	0.22	
*CXCL13*	uni.	0.21(0.1–0.46)	*1.5 × 10^−5^*	0.69	0.76(0.28–2.1)	0.6	0.545
	*CXCL13*	0.24(0.11–0.54)	*4.5 × 10^−4^*	0.721	0.83(0.29–2.37)	0.73	0.739
	node	0.69(0.32–1.49)	0.35		3.61(1.22–10.71)	*0.021*	
	size	1.71(0.83–3.55)	0.15		2.12(0.86–5.22)	0.1	

**Figure 5.**
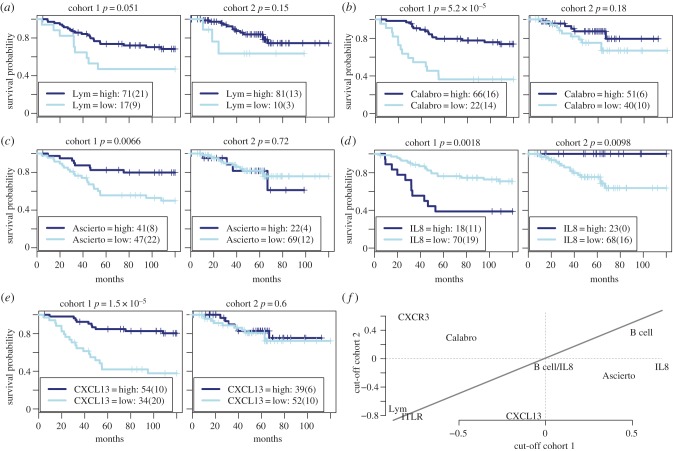
Comparison of ITLR with other immune signatures. The optimal cut-off was selected in cohort 1 and tested in cohort 2 for (*a*) image-based lymphocyte abundance (Lym); (*b*) gene expression immune signature by Calabro *et al.* [19]; (*c*) Ascierto *et al*. [20]; (*d*) *IL8* signature [21]; (*e*) *CXCL13* expression. (*f*) Comparing optimal cut-offs selected in two cohorts. Data were centred at 0 and scaled to have standard deviation 1 and cut-offs were mapped to the centred, scaled data. Signatures close to the diagonal line have similar cut-offs in two cohorts. (Online version in colour.)

We compared ITLR with eight other immune signatures. These include our previously published image-based signature, lymphocyte abundance (Lym), defined as the ratio between the number of lymphocytes and the number of cancer cells (Methods) [13]. The difference between ITLR and Lym is that Lym does not account for different classes of lymphocytes, whereas ITLR considers infiltrating lymphocytes. The remainder of signatures are published gene expression-based signatures from Calabro *et al.* [19] that are predictive of ER-negative breast cancer prognosis, a five-gene signature from Ascierto *et al*. [20] that predicts recurrence-free survival across breast cancer subtypes, and the B-cell, *IL8* and combined signatures to predict prognosis of TNBC [21]. *CXCR3* and *CXCL13* expression were also included because they have been shown to correlate with breast cancer prognosis [22,23]. We applied the same cut-off selection approach to test the association between these signatures and disease-specific survival (electronic supplementary material, table S1). The signatures that showed the best prognostic values are shown in figure 5*a–e* (all are provided in the electronic supplementary material, figure S3) and table 1. None of these signatures correlated with prognosis in both cohorts. This analysis was repeated using cohort 2 as the discovery cohort for selecting the optimal cut-offs and cohort 1 for validation (electronic supplementary material, figure S4 and table S2). In both experiments, only ITLR consistently stratified patients into two groups of different outcome among the nine signatures (electronic supplementary material, figures S3–S4). Furthermore, we compared the best cut-offs selected in two cohorts for all nine signatures (Methods; figure 5*f*). ITLR was among the most consistent signatures in terms of optimal cut-offs in two cohorts, supporting the consistency and the potential use of ITLR as an objective measure for identifying patients with low lymphocytic infiltration.

Compared with published immune signatures, ITLR was also the only signature to show significant correlation with disease-specific survival in multivariate Cox proportional hazards model together with standard clinical parameters of nodal status and tumour size in both cohorts, whichever cohort was used as the discovery cohort (table 1 and electronic supplementary material, tables S1 and S2). Using samples from both cohorts, ITLR has a log-rank *p*-value of 2.1 × 10^−4^ and HR 0.32 (0.17–0.58). To test the robustness of the Cox model in determining the prognostic value of ITLR, we used bootstrap analysis in randomly perturbed data and repeated the univariate and multivariate regression analysis 1000 times. In 95.6% and 94.7% of the time, ITLR remained significantly associated with prognosis in univariate and multivariate analysis, respectively. Taken together, our data support the stability and robustness of ITLR as an independent prognostic biomarker in TNBC.

### 2.3. ITLR heterogeneity is reflected on the transcriptional level by *CTLA*4 and *APOBEC3G* expression

To identify molecular associations of immune infiltration and to test the biological relevance of ITLR, we integrated image-based ITLR with microarray gene expression data profiled for the same set of 181 TNBC tumours. The analysis identified 307 genes positively correlated and 105 genes negatively correlated with ITLR (false discovery rate (FDR) multiple testing correction, *q*-value < 0.05; Methods). Genes with the most significant correlations with our immune signature ITLR include kinases (*SH3KBP1*, *LCK*, *MAP4K1*) and receptors (*FCRL3*, *GPR18*, *TNFRSF13B*, *SEMA4D, CXCR3, IL2RG*), as well as the known immunotherapy target *CTLA*4 (electronic supplementary material, table S3). Thus, significant correlations between ITLR- and immune-related genes support the biological relevance of the proposed ITLR signature.

Subsequently, enrichment analysis was performed on the positively and negatively correlated genes, respectively, against MSigDB gene set categories [24], including KEGG pathways [25], canonical pathways curated by domain experts and immunological signatures (Methods and electronic supplementary material, figure S5). Genes positively correlated with ITLR are enriched with natural killer cell-mediated cytotoxicity, T cell receptor, antigen processing and presentation KEGG pathways, *CD8* T cell, *CD4* T cell and B cell upregulated immunogenic signatures, as well as *IL12* and *CD8 TCR* canonical pathways (electronic supplementary material, tables S5–S9). Conversely, genes negatively correlated with ITLR were enriched with ECM receptor interaction and focal adhesion KEGG pathways, regulatory T cell and *TGFβ*-related immunological signatures as well as integrin-related pathways (electronic supplementary material, table S10–S12). The molecular analysis on the pathway level suggests ITLR is positively associated with anti-tumour immune activities in TNBC.

**Figure 6.**
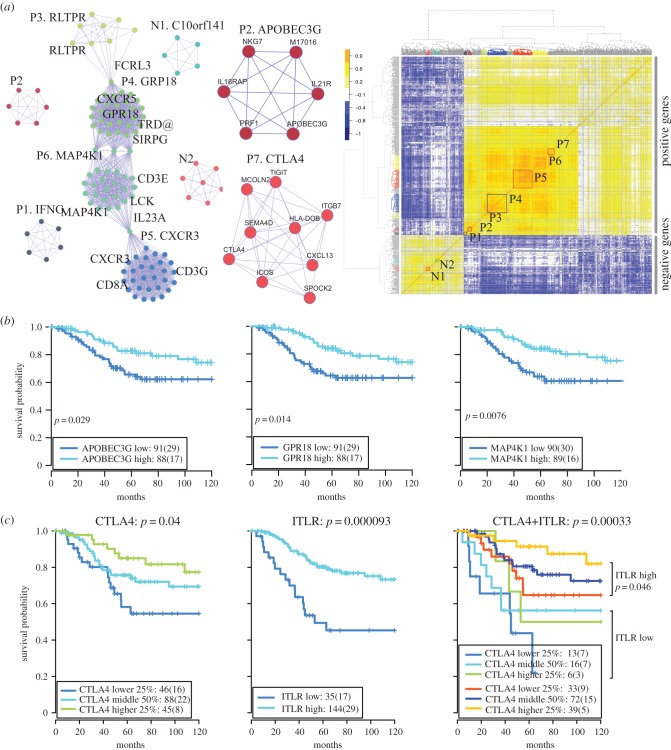
ITLR-associated gene modules. (*a*) Networks illustrating ITLR-associated gene modules inferred from correlation matrix. Nine tightly connected gene modules were identified using hierarchical clustering of the correlation matrix. This correlation matrix was computed with expression of the genes positively or negatively associated with ITLR, shown as a heatmap (left bottom). These modules were colour-coded to be differentiated from each other. *CTLA4* and *APOBEC3G* modules were illustrated. (*b*) Kaplan–Meier curves to illustrate differences in disease-specific survival of patient groups of equal sizes stratified based on the expression of key genes in three modules. (*c*) Kaplan–Meier curves to illustrate differences in disease-specific survival of patient groups stratified with *CTLA4* expression by the lower 25, middle 50 and higher 25 percentiles, ITLR, and *CTLA4* and ITLR combined. Survival difference between *CTLA* low and high stratification within the ITLR-high group is given as a *p*-value.

To further dissect their interconnected relationships and discover de novo molecular modules, tightly connected gene modules were identified within ITLR-associated genes (figure 6*a*; electronic supplementary material, figure S6; Methods). As such, seven modules of positively correlated genes (P1–P7), and two modules of genes negatively correlated with ITLR (N1 and N2) were identified. Known immune-related genes in the modules include *IFNG* (P1), *RLPTR* (P3), *GPR18* (P4), *CXCR3* (P5), *MAP4K1* (P6), *CTLA4* (P7), *ANXA2* (N1) and *FAP* (N2). Notably, two of the modules contain *APOBEC3G* (P2) and *CTLA4* (P7), which may suggest co-regulation among *APOBEC3G*, *NKG7* and interleukins, including *IL21R* and *IL18RAP,* as well as high correlations among *CTLA4*, chemoattractant for B lymphocytes *CXCL13* [2] and *TIGIT* T cell immunoreceptor with *Ig* and *ITIM* domains (electronic supplementary material, table S13). Furthermore, expression profiles of these genes were significantly associated with disease-specific survival in TNBC, including *APOBEC3G* as well as *GPR18* (P4) and *MAP4K1* (P6) ranked as the top ITLR-associated genes (figure 6*b* and electronic supplementary material, figure S7). *CTLA4* expression was able to stratify patients into groups with significantly different prognosis, and could further stratify the ITLR-high group into two subgroups with significantly different outcomes (*p* = 0.046, figure 6*c* and electronic supplementary material, figure S7). Comparing ITLR with ITLR-associated genes in terms of prognostic value, multivariate analysis showed that ITLR stratification has additional, and in many cases superior, value to ITLR-associated genes (electronic supplementary material, figure S8 and Methods).

## 3. Discussion

This paper describes, to the best of our knowledge, the first study to identify categories of lymphocytes based on statistical analysis of tumour spatial heterogeneity and demonstrate their clinical implications using samples from a large number of patients. A standardized scheme is urgently needed for measuring heterogeneity of lymphocytic infiltration in tumours. The ability to generate reproducible, quantitative scores will provide new opportunities for incorporating immune infiltration into staging of cancer, for example the use of immunoscore for colorectal cancer [9]. We have developed a fully automated computational approach based on image analysis and statistical modelling to dissect the cellular and spatial heterogeneity of TNBC. Using H&E-stained whole-tumour slides, cancer cells and lymphocytes were identified and their spatial relationships quantified based on a kernel density method. Using unsupervised learning, three categories of lymphocytes were identified based on their spatial proximities to cancer.

These lymphocyte categories are consistent with a pathological quantification scheme that considers intratumoral, adjacent stroma and distant stroma compartments [26]. Statistically, these clusters are stable, reported as the optimal clustering solution 97% of the time upon repeated sampling. Furthermore, we demonstrated their significant differences both in spatial distance to the nearest cancer cell and spatial positioning of surrounding cancer cells, supporting their biological relevance. For instance, an intratumour lymphocyte defined in this study is, on average, 7 μm away from a cancer cell and 3 μm from the centroid of a convex hull region formed by nearby cancer cells. An adjacent-tumour lymphocyte may be also close to the nearest cancer cells, but would be further away from the centroid of the convex hull region because it is not surrounded by cancer cells. Thus, the new classification approach is based on spatial measures that account for spatial positioning of cancer cells while being computationally efficient enough to analyse whole-tumour sections. Compared with our previously reported measure of lymphocyte abundance as a direct output from image analysis [13], the new approach offers a step forward by accounting for the spatial heterogeneity of immune infiltration recognized as an important property of immune infiltration [1] but rarely quantitatively analysed.

As a result, we propose a new spatial and quantitative measure of intratumour lymphocytes (ITLR). Furthermore, we demonstrate that this measure is a consistent, stable and independent predictor of disease-specific survival across two independent cohorts of 181 TNBC patients in total. Our measurement uses an optimal cut-off of 0.011 (1.1% of infiltrating lymphocytes to cancer cells) that dichotomizes the ITLR score. Indeed, the approximately 20% of TNBC patients with ITLR scores lower than this cut-off have significantly worse disease-specific survival than patients with higher scores, and this association is independent of standard clinical parameters. Taken together, our data support the potential of ITLR as a prognostic biomarker for TNBC, and we anticipate further validation of its prognostic value in larger patient cohorts. Further, its predictive value for treatment response like other measures of immune infiltration [2,3] remains to be investigated. Nevertheless, the highlight of this study is an objective, fully automated scoring system as a step towards standardized assessment of immune infiltration that can potentially be used in the context of clinical trials and subsequently aid the treatment decision-making process.

ITLR measures the ratio of intratumour lymphocytes to cancer cells, thus is different to the pathological assessment approach described in references [2,3,12], where the proportion of tumour nests that were infiltrated by lymphocytes were reported. Nevertheless, in agreement with our findings, tumour-infiltrating lymphocytes were found to be significantly correlated with favourable outcome in TNBC. All these approaches are based entirely on H&E-stained pathological samples and, although their different strengths remain to be compared, together these support that measures of lymphocytic infiltration can be useful tools to aid clinical decisions in TNBC. We showed that the image-based ITLR outperforms some of the gene-expression-based signatures using the optimal cut-off selection method, but a more comprehensive analysis is needed to compare it with all prognostic gene expression signatures reported in the literature. However, considering the cost of microarray data acquisition, we reason that our approach opens a new avenue for large-scale analysis on readily available pathological samples.

Furthermore, ITLR as an unbiased assessment of immune infiltration facilitates the discovery of molecular correlates with this clinically important phenomenon. While the expression of many immune-related genes was significantly associated with ITLR, it is unclear whether these genes are expressed on cancer cells or lymphocytes. This is because the microarray data were obtained using whole-tumour materials without microdissection. Our data indicate that the RNA expression of cytotoxic T-lymphocyte-associated protein 4 (*CTLA4*), a receptor of the immunoglobulin family and the target of ipilimumab, was significantly associated with ITLR as well as longer disease-free survival in TNBC. This is consistent with the recent observation in none-small cell lung cancers that overexpression of *CTLA4* is associated with reduced death rate [27]. *CTLA4* is expressed in tumour cells in different cancer types [28]. In breast cancer, it has been reported to be expressed in both tumour cells and T cells, and an inverse correlation between *CTLA4* expression and clinical outcome has been previously reported in 60 patients with different breast cancer subtypes [29]. This is in contrast to our observation in TNBC, which highlights the need to investigate immune infiltration by cancer subtypes. A recent study showed that *in situ* mRNA expression of another receptor of the immunoglobulin superfamily, *PDL1*, is associated with increased immune infiltration and favourable recurrence-free survival across different breast cancer subtypes [30]. Such an assay could be useful for further investigation of expression of *CTLA4* as well as other ITLR-associated genes in TNBC. Taken together, our data support further evaluation of *CTLA4* and the potential of *CTLA4*-targeted therapies in TNBC.

In addition, our gene module analysis revealed several tightly connected, functionally related modules with several key genes that warrant further investigation. For example, one module contains *APOBEC3G* (apolipoprotein B MRNA editing enzyme, catalytic polypeptide-like 3G), which is known to play important roles in adaptive and innate immunity and has been investigated extensively in viral infection [31] but its role in breast cancer remains unclear. It is a member of the apolipoprotein B mRNA-editing enzyme, catalytic polypeptide-like editing complex family together with *APOBEC3B*, which was found to be a source of mutagenesis in many major cancer types, including breast cancer [32]. In our TNBC samples, *APOBEC3G* expression is significantly correlated with favourable prognosis (log-rank *p* = 0.02), but not other *APOBEC* members, including *APOBEC3B* (*p* = 0.29). *APOBEC3G* is primarily expressed in *CD4*^+^ T lymphocytes, macrophages and dendritic cells [33]. Our data revealed strong association between *APOBEC3G* and natural killer cell gene *NKG7* and interleukins in this module and support the importance of *APOBEC3G* in TNBC. Therefore, the associations between ITLR and immune-relevant genes, pathways and modules support the validity of ITLR as a measure of lymphocytic infiltration and revealed potential co-regulations of key immune genes, which warrant further investigation to help elucidate the biological processes underlying lymphocytic infiltration with potentially important clinical implications.

Lymphocytes in tumours are known to encompass diverse subclasses, including helper T cells, regulatory T cells, natural killer cells and B cells with sophisticated implications for treatment response [10,23,34]. Admittedly, our current approach is not discriminative for different subclasses of lymphocytes. This could be improved by performing immunohistochemistry analysis with immune cell markers, for which automated immunohistochemistry image analysis and statistical modelling methods could be developed to discern interactions between cancer and anti-/pro-tumoural immune response. Another limitation in this study is the number of samples. Once its mature survival information becomes available, The Cancer Genome Atlas breast cancer dataset [35] with its H&E and matched molecular profiling data will be the ideal cohort to validate the prognostic value and molecular correlates of ITLR. Finally, it is worth noting that our computational approach can be adapted for studying other cancer types that will benefit from objective approaches for scoring immune infiltration, such as melanoma and non-small cell lung cancer.

## 4. Methods

### 4.1. Clinical samples

The complete set of METABRIC [16] samples contains 1980 primary frozen breast tumours from five contributing hospitals. Among these, 1026 of the 1047 tumours from three hospitals have H&E sections without severe artefacts, whereas all H&E samples from the other two hospitals are highly fragmented owing to long-term frozen storage. Therefore, we considered only the 1026 tumours for this study (long-term follow-up median 68.3 months). On average, three tumour sections were obtained from different locations of each primary tumour and placed onto the same slide [13]. Tumour materials sandwiched between these sections were sectioned, mixed and used for molecular profiling, thereby maximizing the biological relevance of multiple data types being generated. Further details on experimental procedure, staining and molecular profiling protocols can be found in reference [13]. Gene expression data were profiled using the Illumina HT-12 platform. *ER* status was determined based on the bimodal distribution of *ESR1* expression microarray data, and *HER2* amplification status based on microarray SNP6 data from the same tumours. In total, there are 181 *ER*-negative, *HER2*-negative samples, and we conventionally defined them triple-negative/TNBC. Samples from two of the three hospitals were merged to form cohort 1 (89 samples), and samples from the other hospital form cohort 2 (92 samples), in order to obtain a similar population size in each cohort. Immune infiltration was scored for 112 of the 181 samples by the pathologists in the METABRIC consortium into three categories: absent, mild and severe: absent if there were no lymphocytes, mild if there was a light scattering of lymphocytes and severe if there was a prominent lymphocytic infiltrate. The pathological scores of immune infiltration were not significantly correlated with prognosis (electronic supplementary material, figure S9).

### 4.2. Haematoxylin and eosin image analysis

We have previously validated the accuracy of our automated image analysis tool for H&E breast tumour frozen section images based on pathological tumour scores and cell-by-cell evaluation [13]. For METABRIC samples, this tool achieved 90% cross-validation accuracy for cell classification and high correlation with pathological scores of cell proportions (cor. = 0.98) [13]. This tool was used to classify all cell nuclei in 181 TNBC whole-tumour sections, resulting in an average of 81 810 (s.d. 80 330) cancer cells, 15 500 (25 133) lymphocytes and 14 090 (14 180) stromal cells for each image. Lymphocytes have a typical morphology of small, round and homogeneously basophilic nuclei, thus can be reliably differentiated from other cell types in breast cancer. Because this analysis is based on nuclear morphology only in the H&Es, the identified lymphocytes are likely to be a mixture of immune cell types, including T- and B-lymphocytes.

### 4.3. Modelling the spatial heterogeneity of cancer–immune interaction

Let *x* = *x*_1_, *x*_2_, … , *x_n_* be the spatial locations of *n* cancer cells, and *y* = *y*_1_, *y*_2_, … , *y_m_* be the spatial locations of *m* immune cells in an H&E tumour section image. Using a quartic kernel function ***K***, we can establish a kernel density estimate over the whole-tumour slide: 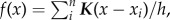 where *h* is the bandwidth parameter for ***K***. *h* was optimized using the minimum square error criteria [36] in 10 randomly sampled images as described in the electronic supplementary material, Sweave file. Thus, the spatial proximity to cancer for an immune cell *i* is *s_i_* = *f*(*y_i_*). We can then identify lymphocyte classes based on ***s***, ***s*** = *s*_1_, *s*_2_, … , *s_m_* using unsupervised Gaussian mixture clustering [37]. This method aims to identify multiple components/clusters within the data with probabilities that quantify the uncertainty of observations belonging to the clusters.
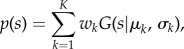
where *K* is the number of clusters, *μ*_*k*_ and *σ*_*k*_ are the mean and variance that define the probabilistic density function *G* for the *k*th component, and *w*_*k*_ is the weight of a component *k*. These parameters were estimated by expectation–maximization [38]. Selection of models with different numbers of clusters can be done using statistical criteria, one of the most common being the BIC [18]. It can be used in conjunction with mixture model clustering to select the best number of clusters *K*
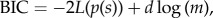
where *L*() is the maximum log likelihood function and *d* is the number of free parameters to be estimated. Effectively, the BIC aims to evaluate modelling error as well as model complexity. The higher the value of BIC, the better the solution is considered to be. To perform clustering, 100 000 immune cells were randomly sampled. Their spatial proximity to cancer data ***s*** was used for clustering with a range of different *K*, *K* = 1–5. This was repeated 200 times, in 97% of which the solution with three clusters was considered the optimal by BIC. Mean *μ*_*k*_ of the clusters are consistent (median: 0.011, 0.06, 0.13; s.d.: 0.002, 0.0047, 0.0045). Subsequently, we classified all lymphocytes in all tumour samples based on these clusters. We used the ratio of the number of intratumour lymphocytes and the number of cancer cells as the final measurement of intratumour immune infiltration
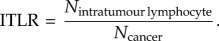


### 4.4. Measuring cell distances and spatial arrangement

To identify physical properties of ITLs, ATLs and DTLs that differentiate them, in 10 000 lymphocytes randomly sampled from 20 tumours, we identified the five nearest cancer cells and the centroid of the convex hull region formed by these cancer cells. The distance from a lymphocyte to the nearest cancer cell (*d*_min_) and to the centroid of the convex hull (*d*_centroid_) were computed. Centroid of a convex hull region was calculated as the mean positions of the subset of points that define the convex hull. Differences among lymphocyte classes in terms of *d*_min_ and *d*_centroid_ were tested with Student's *t*-test.

### 4.5. Other immune signatures in comparison

Lymphocyte abundance based on our image analysis result was calculated as
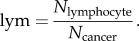


The gene expression signatures were calculated as described in the referred papers.

### 4.6. ITLR gene modules

Hierarchical clustering was used to identify highly correlated gene modules by clustering the correlation matrix of all ITLR-associated genes into 100 clusters. Modules were selected from these clusters based on average absolute Pearson correlation exceeding 0.75 and cluster size exceeding five.

### 4.7. Comparing ITLR and ITLR-associated genes

To test whether ITLR has additional value to ITLR-associated genes, we performed multivariate Cox regression analysis with ITLR paired with the expression profile of an ITLR gene. This was performed for all of the top 100 ITLR-associated genes ranked by correlation. ITLR was dichotomized using the threshold reported in the paper, and gene expression was dichotomized into two equal-size groups or three groups (25 lower, 50 middle and 25 upper percentiles). Tables with hazard ratio, log-rank *p*-value and 95% confidence interval were produced. In both analysis with two and three patient groups according to gene expression data, *p*-values of ITLR were consistently higher than the *p*-values of gene expression profiles, as well as being higher than significance level of 0.05 (−log(*p*) 2.99).

### 4.8. Other statistical methods

Monotone trend between ITLR and clinical parameters was tested using the Jonckheere–Terpstra trend test [39]. Survival analysis was performed with breast-cancer-specific 10-year survival data. The Kaplan–Meier estimator was used for patient stratification and log-rank test was used for testing difference among groups. Cox proportional hazards regression model was fitted to the survival data and hazard ratios and 95% CIs were computed to determine the correlation with disease-specific survival, where the log-rank test with *p* < 0.05 was considered significant. Correlation between ITLR and gene expression was computed with Pearson correlation and *q*-values computed from FDR correction using 25% of the data for fitting the null model. Cut-offs for dichotomizing all immune signatures were optimized stepwise from 20 to 80 percentiles at an interval of 1.5. The cut-offs that displayed the highest prognostic significance with log-rank test were selected. For a consistency test in figure 5*f*, each signature was centred at 0 and scaled to standard deviation 1. Optimal cut-offs were also mapped to the new data before comparison. MSigDB gene set v. 4.0 [24] was used in conjunction with a hypergeometric test for enrichment analysis.

## Supplementary Material

Supplementary materials

## Supplementary Material

Sweave file

## Supplementary Material

Supplementary Tables

## Data accessibility

For full reproducibility, R code and full datasets for the proposed methodology and reproducing all reported results are provided as Sweave files as part of the electronic supplementary material. Cell types and spatial location data for the entire set of 181 TNBC samples are also provided as part of the data in the electronic supplementary material. Raw pathological images are deposited in the European Genome–Phenome Archive (http://www.ebi.ac.uk/ega/) under accession number EGAS00000000098. Gene expression data are available upon application to the METABRIC consortium.
