# Supplementary material for: Modelling the spatial heterogeneity and molecular correlates of lymphocytic infiltration in triple-negative breast cancer
Source: J R Soc Interface. 2015 Feb 6;12(103):20141153. doi: 10.1098/rsif.2014.1153 (PMC4305416; doi:10.1098/rsif.2014.1153)
Supplement: Supplementary materials [file rsif20141153supp1.pdf]

Supplementary Materials for

Modelling the Spatial Heterogeneity and Molecular Correlates of Lymphocytic Infiltration in Triple-Negative Breast Cancer

Yinyin Yuan

Division of Molecular Pathology and the Centre for Evolution and Cancer,

The Institute of Cancer Research,

London, UK

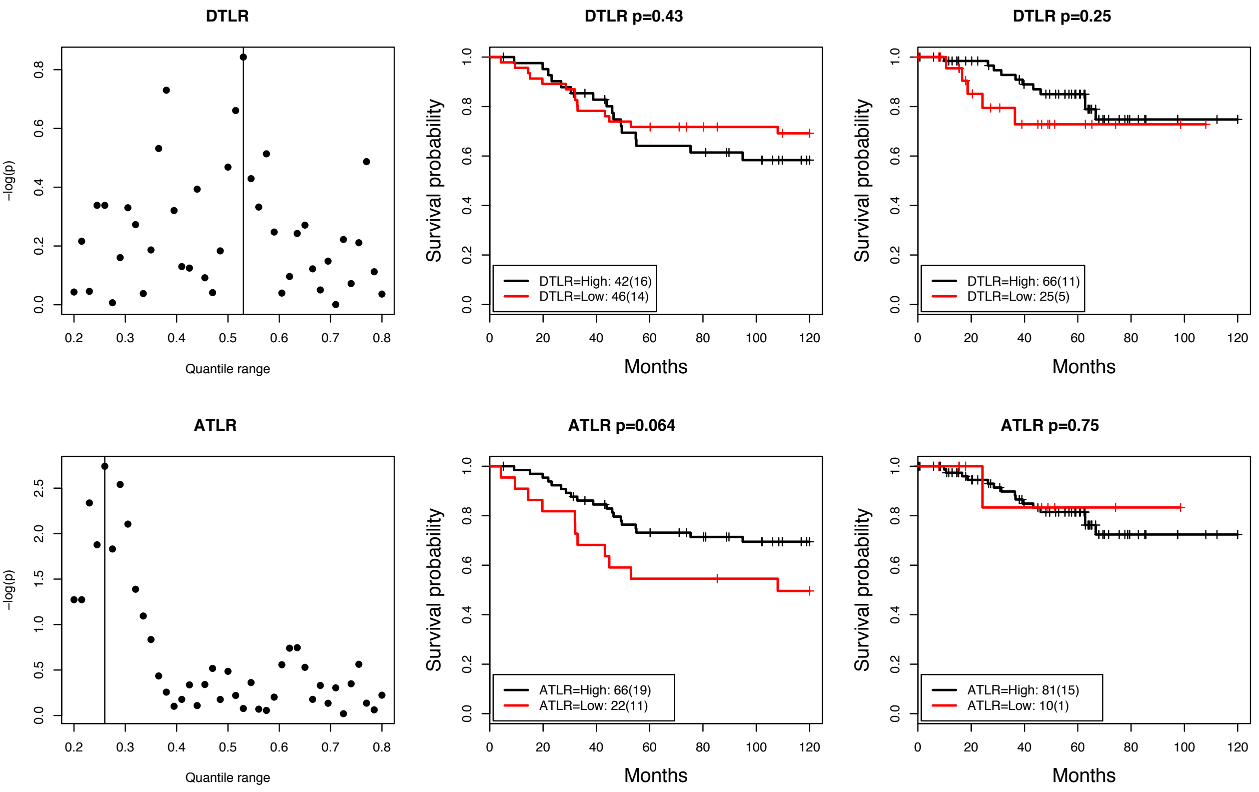

Figure S1. Kaplan-Meier curves to illustrate the disease-specific survival probabilities of patient groups in in two TNBC cohorts stratified by ATLR (Adjacent) and DTLR (Distal). The signatures were dichotomised using a cutoff selected over a range of percentiles based on Cohort 1 (the left and middle columns) and tested in Cohort 2 (the right column). Dashed lines in the plots on the left marks the significance threshold of p=0.05, and solid vertical lines show the best cut-offs. For the Kaplan-Meier curves, the numbers in the legend show the number of patients in each group and numbers in the bracket show the number of disease-specific deaths.

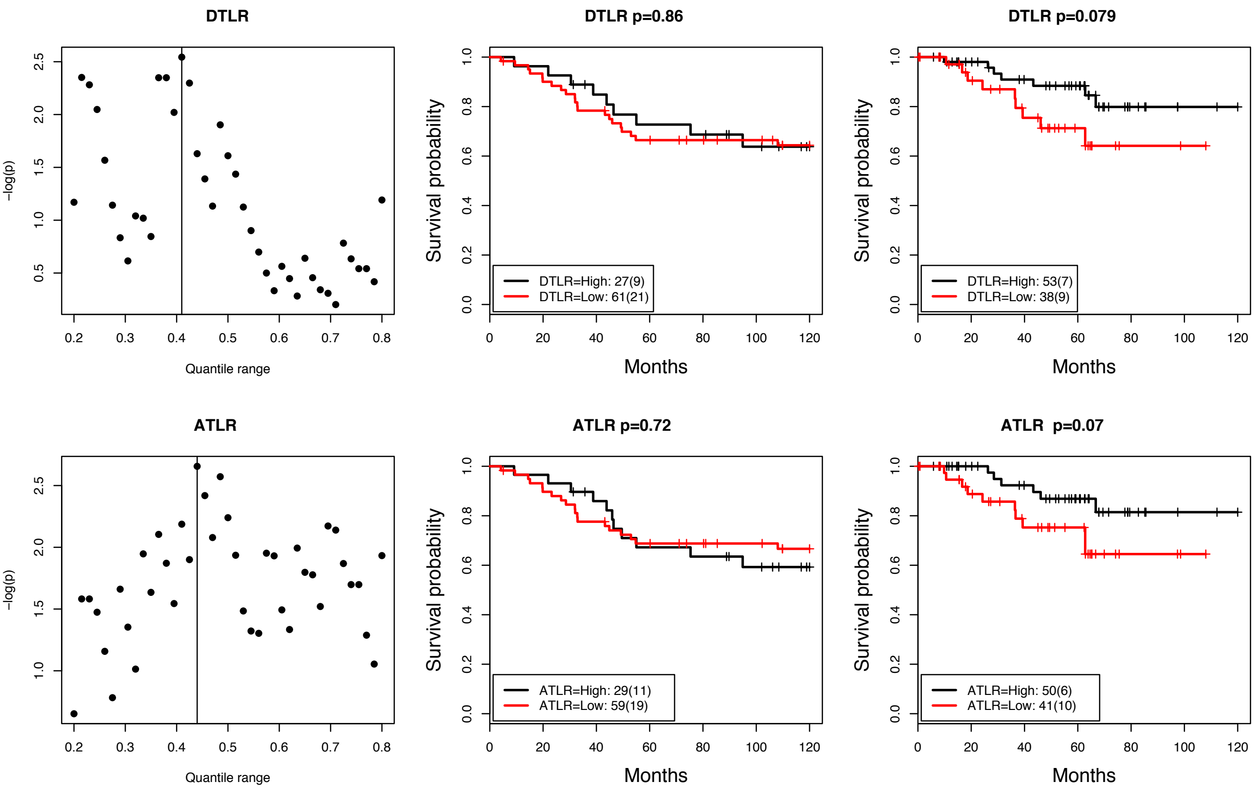

Figure S2. Kaplan-Meier curves to illustrate the disease-specific survival probabilities of patient groups in in two TNBC cohorts stratified by ATLR (Adjacent) and DTLR (Distal). The signatures were dichotomised using a cutoff selected over a range of percentiles based on Cohort 2 (the left and right columns) and tested in Cohort 1 (the middle column).

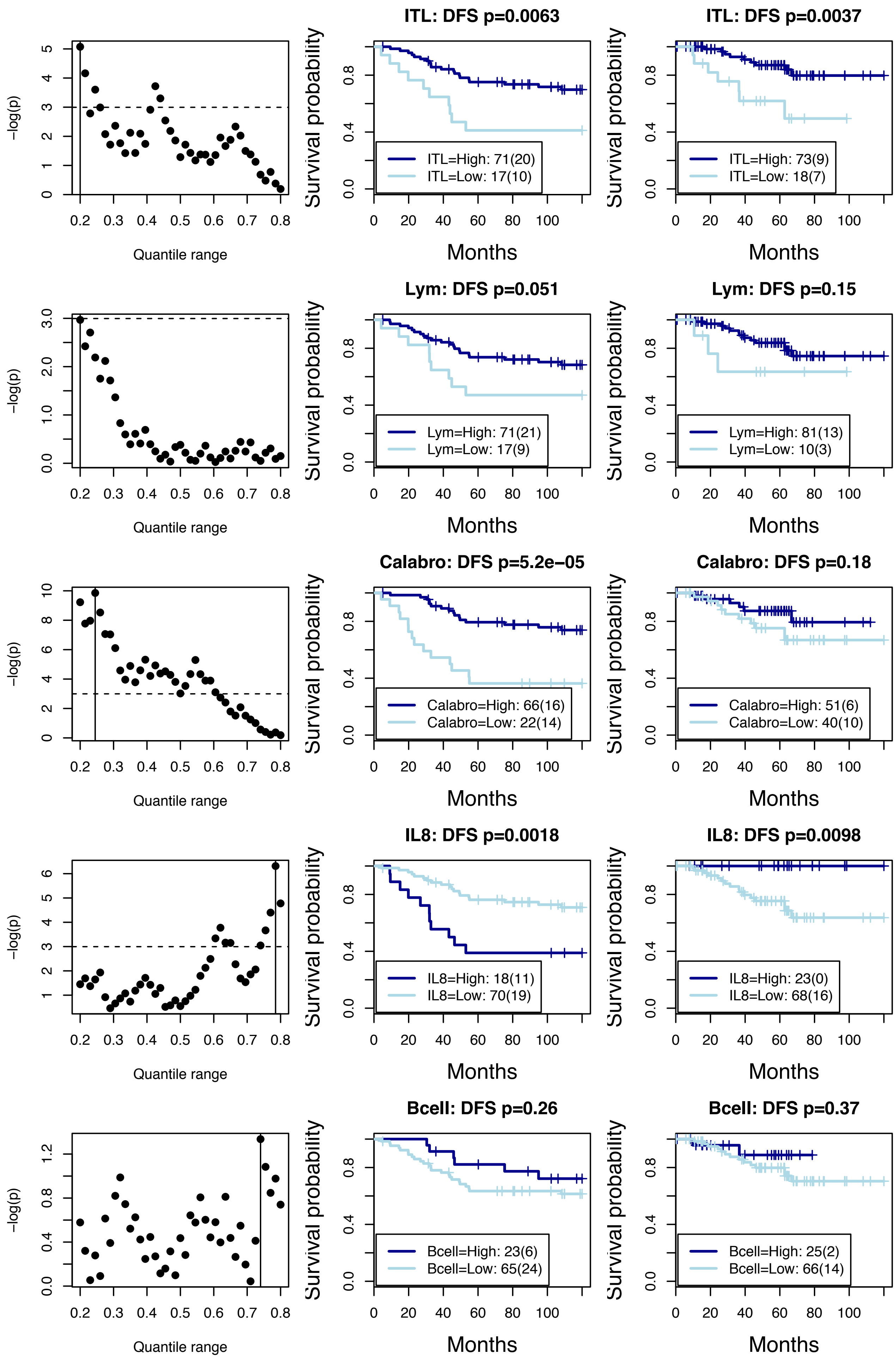

ITL: DFS p=0.0063
ITL: DFS p=0.0037
ITL=High: 71(20)
ITL=Low: 17(10)
ITL=High: 73(9)
ITL=Low: 18(7)
Lym: DFS p=0.051
Lym: DFS p=0.15
Lym=High: 71(21)
Lym=Low: 17(9)
Lym=High: 81(13)
Lym=Low: 10(3)
Calabro: DFS p=5.2e−05
Calabro: DFS p=0.18
Calabro=High: 66(16)
Calabro=Low: 22(14)
Calabro=High: 51(6)
Calabro=Low: 40(10)
IL8: DFS p=0.0018
IL8: DFS p=0.0098
IL8=High: 18(11)
IL8=Low: 70(19)
IL8=High: 23(0)
IL8=Low: 68(16)
Bcell: DFS p=0.26
Bcell: DFS p=0.37
Bcell=High: 23(6)
Bcell=Low: 65(24)
Bcell=High: 25(2)
Bcell=Low: 66(14)
−log(p)
Quantile range
Survival probability
Months

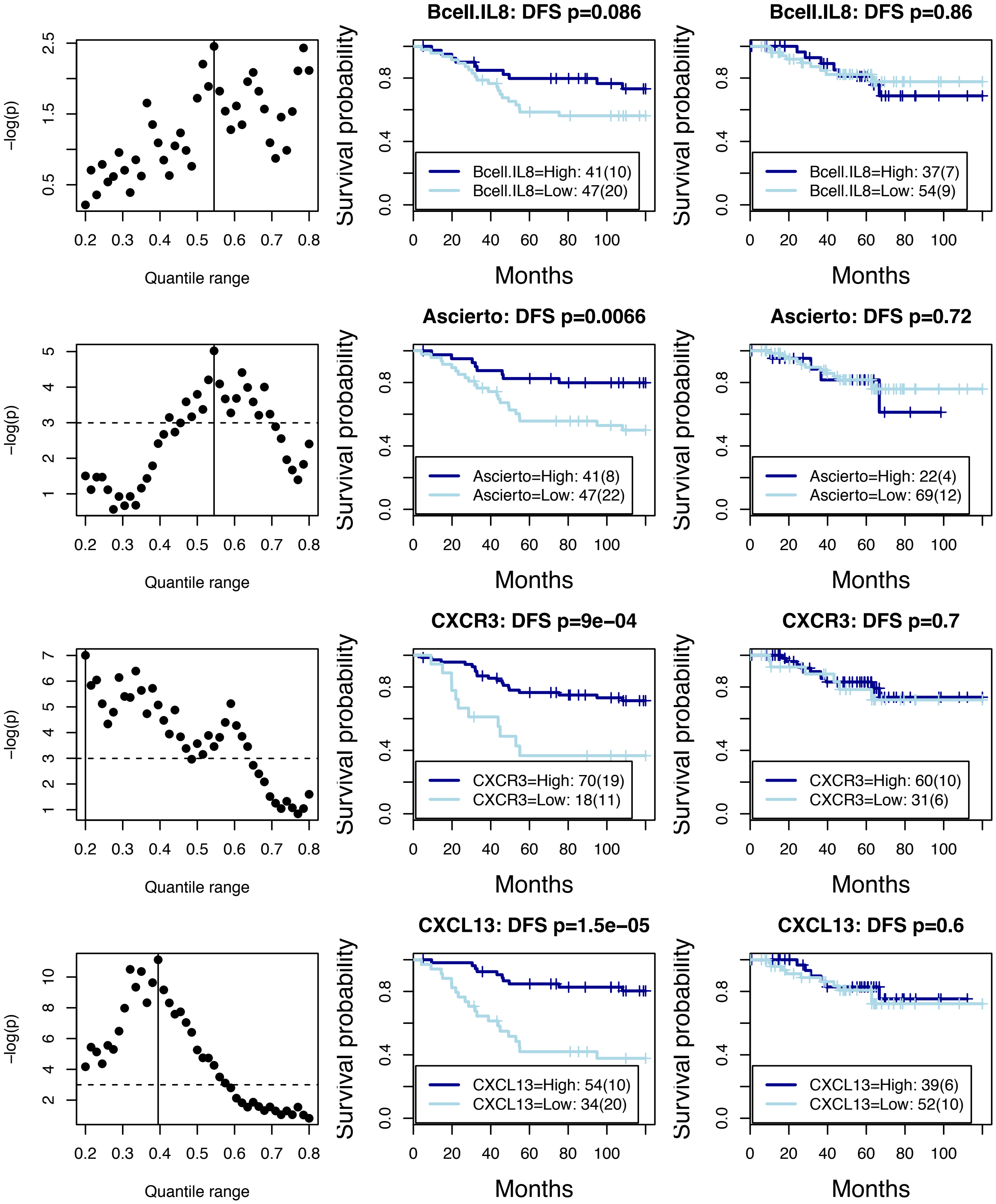

Figure S3. Kaplan-Meier curves to illustrate the disease-specific survival probabilities of patient groups in in two TNBC cohorts stratified by nine immune signatures. The signatures were dichotomised using a cutoff selected over a range of percentiles based on Cohort 1 (the left and middle columns) and tested in Cohort 2 (the right column). Dashed lines in the plots on the left marks the significance threshold of p=0.05, and solid vertical lines show the best cut-offs. For the Kaplan-Meier curves, the numbers in the legend show the number of patients in each group and numbers in the bracket show the number of disease-specific deaths.

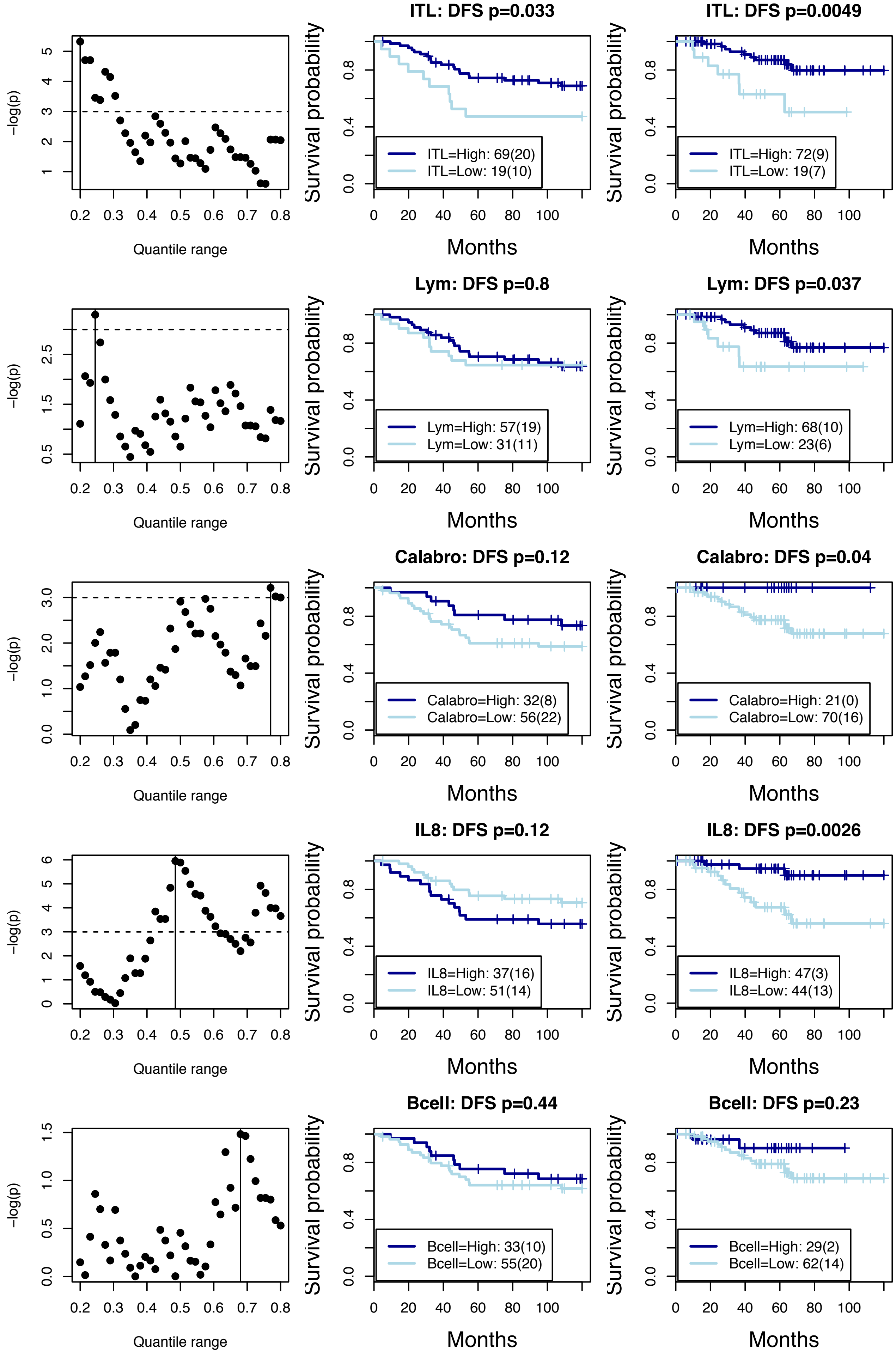
ITL: DFS p=0.033
ITL: DFS p=0.0049
ITL=High: 69(20)
ITL=Low: 19(10)
ITL=High: 72(9)
ITL=Low: 19(7)
Lym: DFS p=0.8
Lym: DFS p=0.037
Lym=High: 57(19)
Lym=Low: 31(11)
Lym=High: 68(10)
Lym=Low: 23(6)
Calabro: DFS p=0.12
Calabro: DFS p=0.04
Calabro=High: 32(8)
Calabro=Low: 56(22)
Calabro=High: 21(0)
Calabro=Low: 70(16)
IL8: DFS p=0.12
IL8: DFS p=0.0026
IL8=High: 37(16)
IL8=Low: 51(14)
IL8=High: 47(3)
IL8=Low: 44(13)
Bcell: DFS p=0.44
Bcell: DFS p=0.23
Bcell=High: 33(10)
Bcell=Low: 55(20)
Bcell=High: 29(2)
Bcell=Low: 62(14)
−log(p)
Quantile range
Survival probability
Months

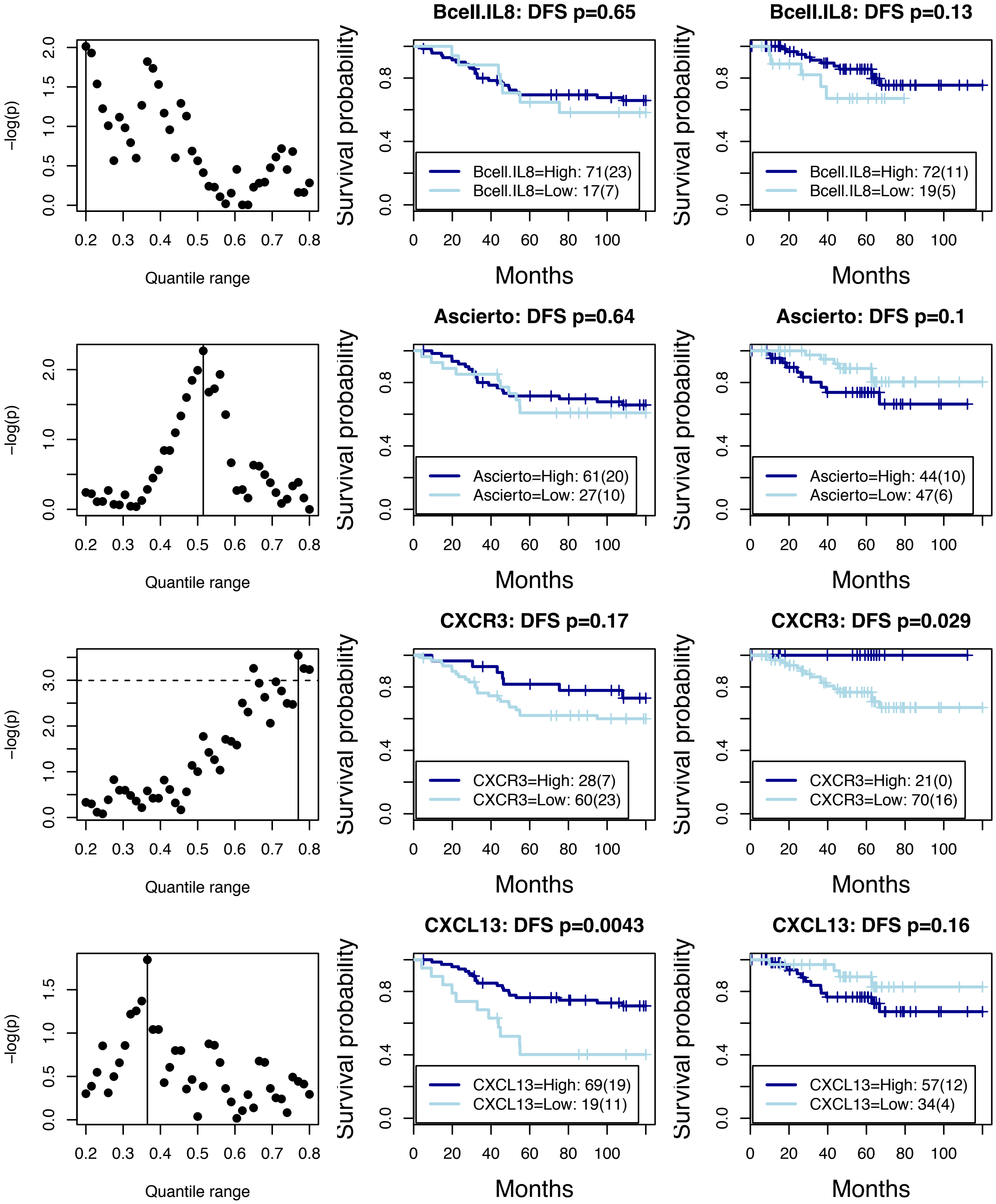

Figure S4. Kaplan-Meier curves to illustrate the disease-specific survival probabilities of patient groups in in two TNBC cohorts stratified by nine immune signatures. The signatures were dichotomised using a cutoff selected over a range of percentiles based on Cohort 2 (the left and right columns) and tested in Cohort 1 (the middle column). Dashed lines in the plots on the left marks the significance threshold of p=0.05, and solid vertical lines show the best cut-offs. For the Kaplan-Meier curves, the numbers in the legend show the number of patients in each group and numbers in the bracket show the number of disease-specific deaths.

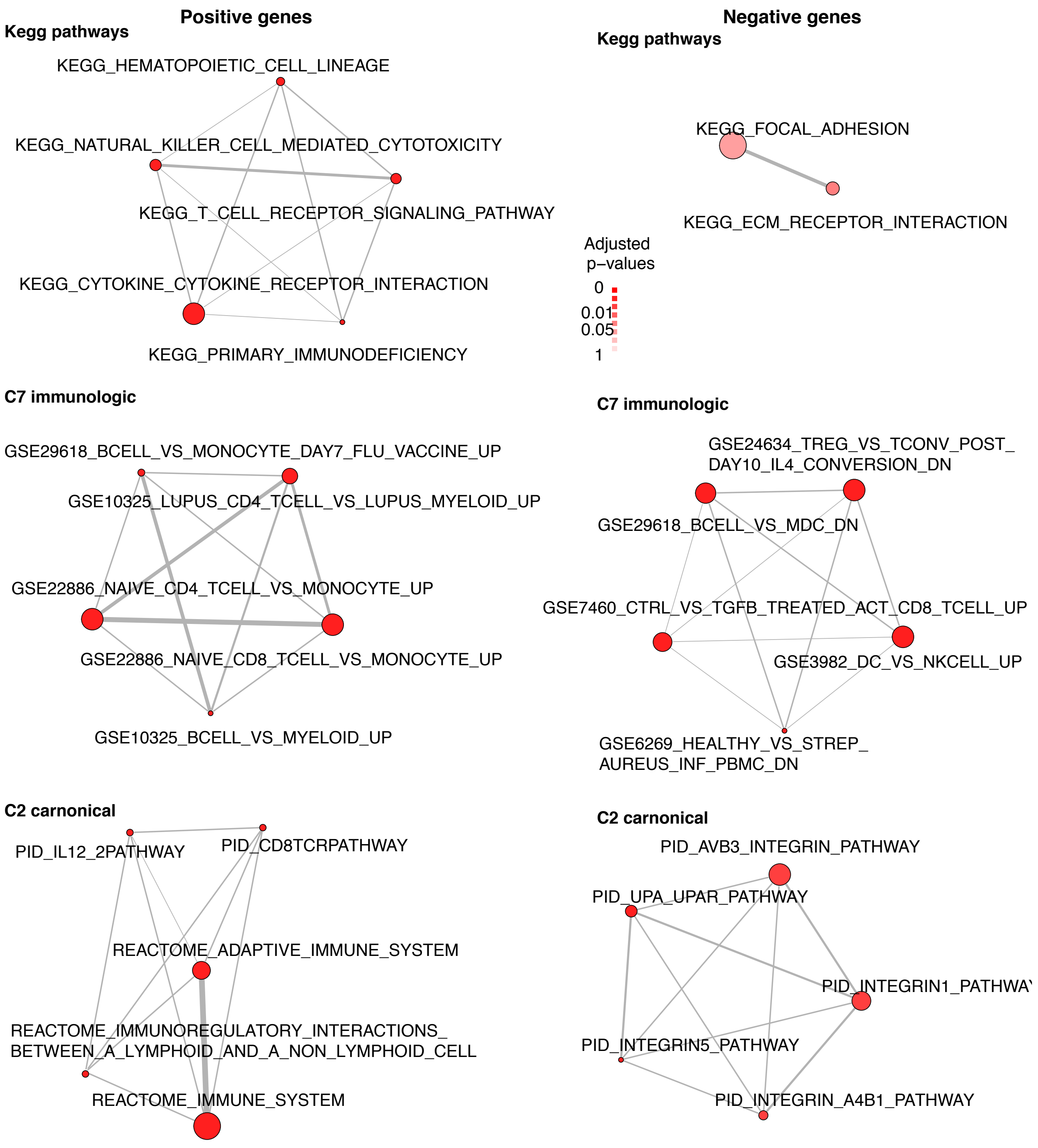

Figure S5. MSigDB enrichment of ITL-associated genes. Enrichment maps to show the KEGG pathways, immunologic signatures and canonical pathways enriched in the genes positively (left) or negatively (right) correlated with ITL. Node size is proportional to the size of the gene set and line thickness is proportional to the overlap between gene sets.

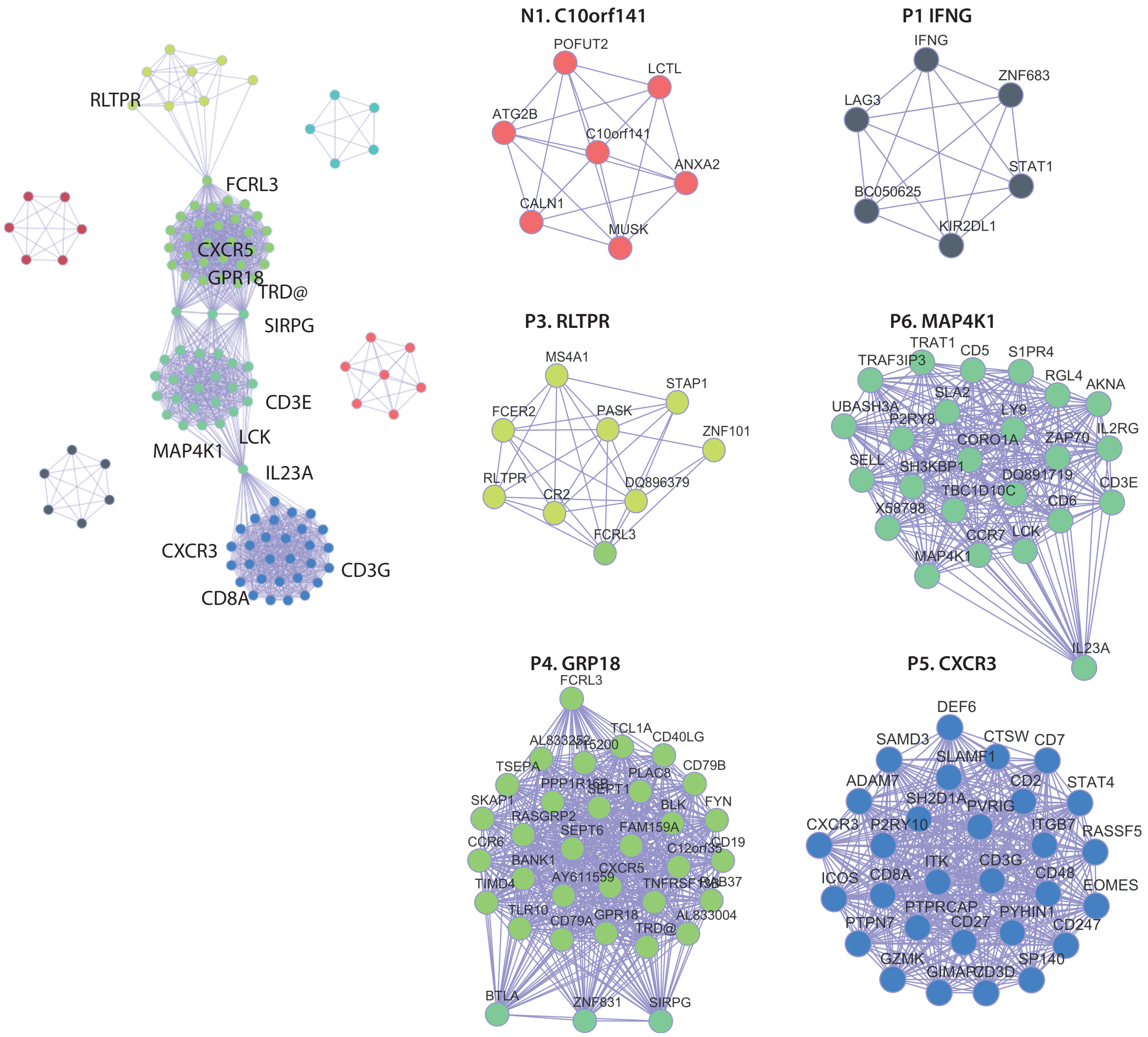

Figure S6. ITL-associated gene modules (continued).

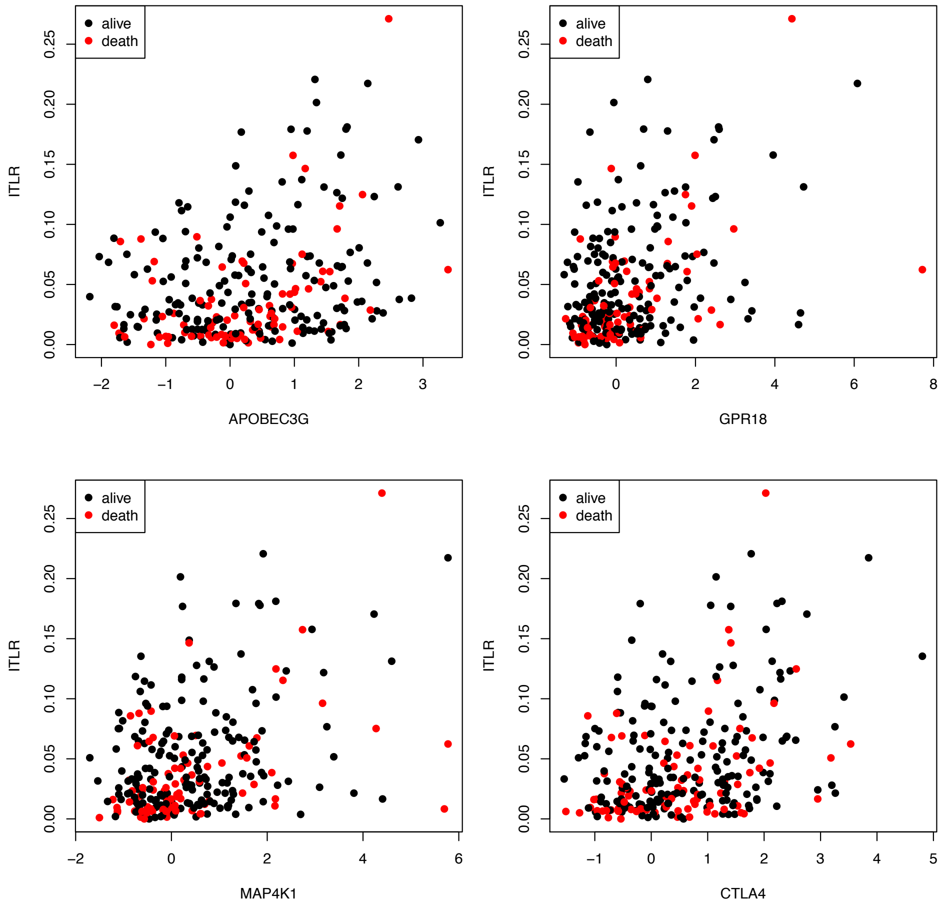

Figure S7. Scatter plots to show correlation between ITLR and expression of ITLR-associated genes in TNBC.

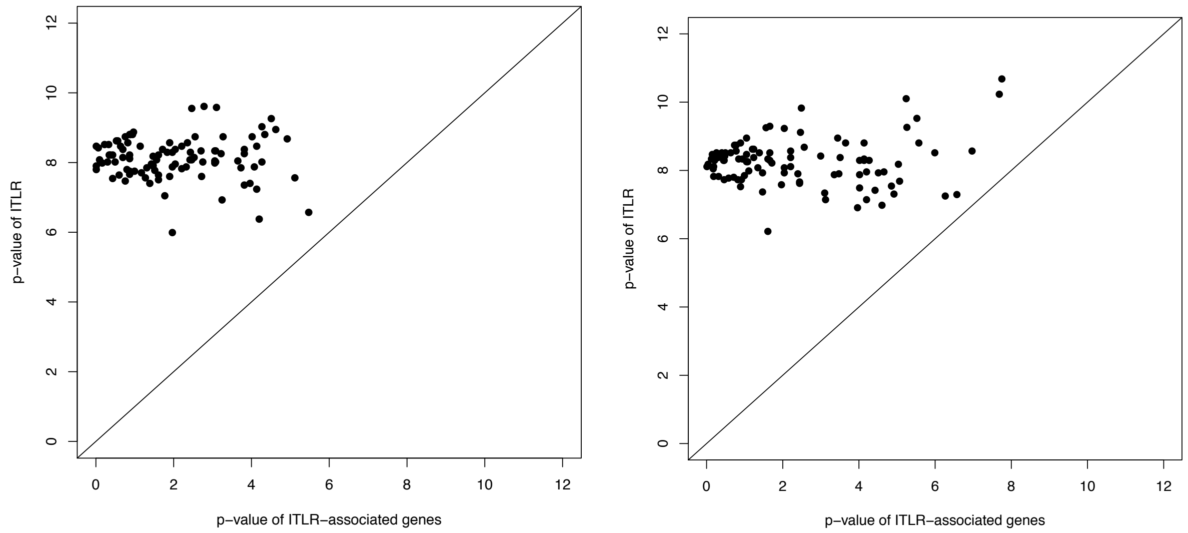

Figure S8. Compare the prognostic value of top 100 ITLR-associated genes and ITLR by including both in multivariate Cox analysis model, one gene at a time. Each point denotes analysis for one gene, plotted values are –log(log rank *p*-value) for the analysis.

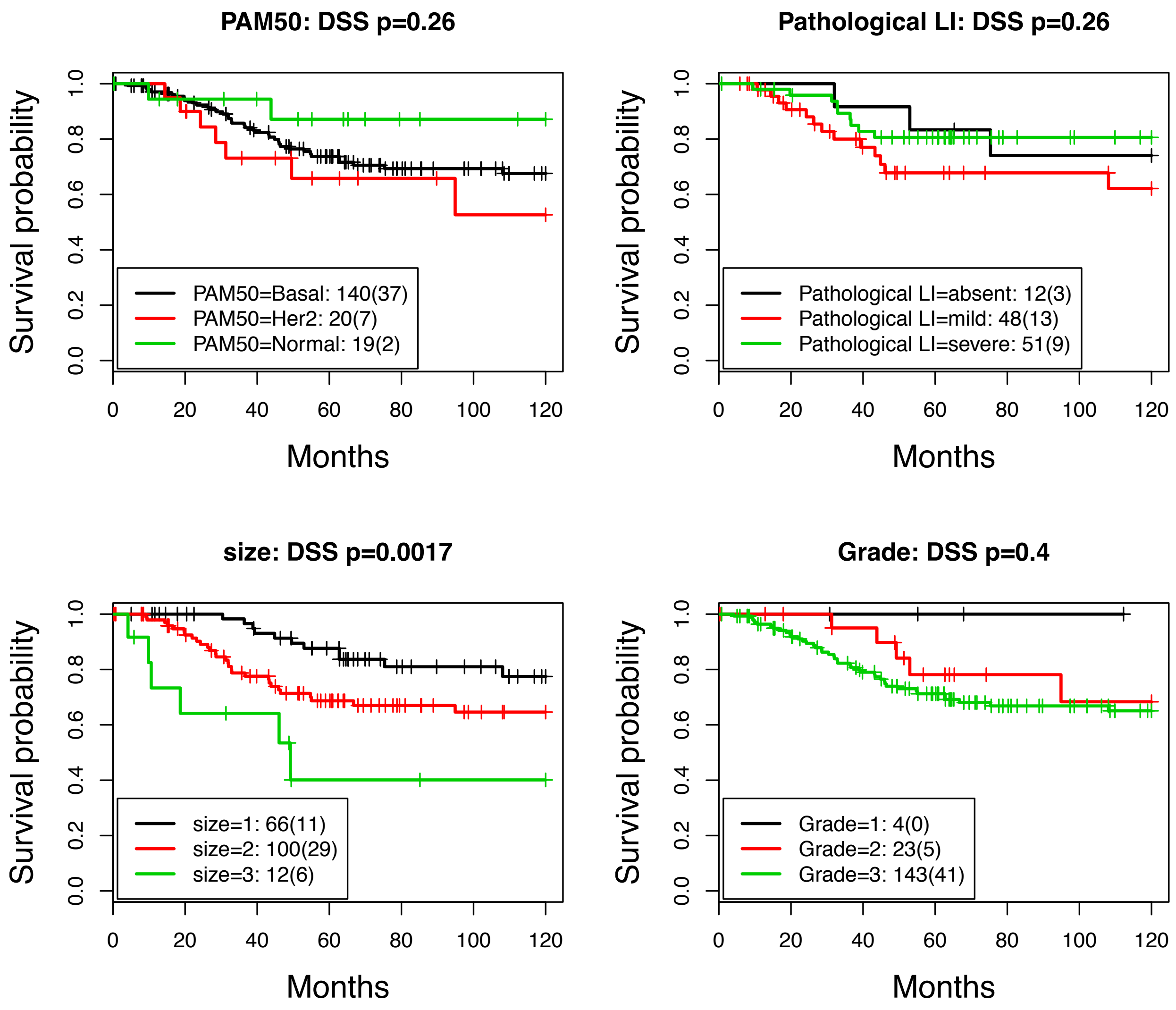

Figure S9. Kaplan-Meier curves illustrating differences in disease-specific survival of TNBC patients stratified with other known parameters including PAM50 (27), pathological assessment of LI, tumour size, and grade.

Supplementary Tables

Table S1. Univariate and multivariate Cox regression results for ITL and other eight signatures using the optimal cut-offs selected in Cohort 1 and validated in Cohort 2. Uni-: Univariate Cox regression; Multi-: Multivariate Cox regression; HR: Hazard Ratio; CI: lower and higher 95% Confidence Interval; Conc: Concordance; Inf: Cox model failed to converge.

	**Cohort1**			**Cohort2**		
	HR(CI)	p-value	conc	HR(CI)	p-value	conc
Uni-ITL	0.36(0.17-0.77)	0.0063	0.601	0.25(0.09-0.69)	0.0036	0.659
Multi-ITL	0.32(0.15-0.7)	0.0042	0.668	0.15(0.05-0.43)	0.00051	0.76
Multi-node	0.63(0.29-1.4)	0.26		4.93(1.61-15.08)	0.0052	
Multi-size	2.62(1.27-5.41)	0.0092		2.07(0.9-4.74)	0.087	
	HR(CI)	p-value	conc	HR(CI)	p-value	conc
Uni-Lym	0.47(0.21-1.02)	0.051	0.574	0.41(0.12-1.43)	0.15	0.575
Multi-Lym	0.48(0.22-1.05)	0.066	0.656	0.23(0.05-1.02)	0.053	0.735
Multi-node	0.69(0.32-1.5)	0.35		4.65(1.46-14.81)	0.0092	
Multi-size	2.35(1.16-4.77)	0.018		1.66(0.65-4.25)	0.29	
	HR(CI)	p-value	conc	HR(CI)	p-value	conc
Uni-Calabro	0.25(0.12-0.52)	5.20E-05	0.66	0.5(0.18-1.39)	0.18	0.587
Multi-Calabro	0.27(0.13-0.56)	0.00038	0.703	0.41(0.14-1.19)	0.1	0.744
Multi-node	0.75(0.35-1.6)	0.45		4.57(1.45-14.37)	0.0093	
Multi-size	2.26(1.07-4.76)	0.032		1.91(0.82-4.46)	0.13	
	HR(CI)	p-value	conc	HR(CI)	p-value	conc
Uni-IL8	3.09(1.46-6.51)	0.0018	0.615	0(0-Inf)	0.0099	0.645
Multi-IL8	2.79(1.32-5.92)	0.0073	0.679	0(0-Inf)	1	0.808
Multi-node	0.81(0.37-1.75)	0.59		3.14(1.06-9.34)	0.039	
Multi-size	2.23(1.08-4.63)	0.031		1.75(0.71-4.28)	0.22	
	HR(CI)	p-value	conc	HR(CI)	p-value	conc
Uni-Bcell	0.6(0.25-1.48)	0.26	0.557	0.51(0.12-2.27)	0.37	0.539
Multi-Bcell	0.57(0.23-1.4)	0.22	0.655	0.48(0.11-2.17)	0.34	0.747
Multi-node	0.7(0.32-1.5)	0.35		3.77(1.27-11.2)	0.017	
Multi-size	2.38(1.19-4.76)	0.014		2.07(0.86-5)	0.11	
	HR(CI)	p-value	conc	HR(CI)	p-value	conc
Uni-Bcell.IL8	0.52(0.24-1.11)	0.086	0.581	1.1(0.41-2.95)	0.86	0.482
Multi-Bcell.IL8	0.53(0.25-1.12)	0.097	0.648	1.22(0.42-3.53)	0.71	0.743
Multi-node	0.74(0.34-1.6)	0.44		3.76(1.24-11.41)	0.02	
Multi-size	2.36(1.17-4.76)	0.016		2.21(0.88-5.54)	0.091	
	HR(CI)	p-value	conc	HR(CI)	p-value	conc
Uni-Ascierto	0.34(0.15-0.77)	0.0066	0.621	1.23(0.4-3.83)	0.72	0.51
Multi-Ascierto	0.39(0.17-0.88)	0.024	0.671	1.18(0.37-3.72)	0.78	0.735
Multi-node	0.85(0.39-1.84)	0.68		3.6(1.21-10.7)	0.021	
Multi-size	2.06(1.02-4.16)	0.044		2.16(0.86-5.45)	0.1	
	HR(CI)	p-value	conc	HR(CI)	p-value	conc
Uni-CXCR3	0.3(0.14-0.64)	9.00E-04	0.618	0.82(0.3-2.25)	0.7	0.535
Multi-CXCR3	0.31(0.15-0.66)	0.0026	0.683	0.79(0.25-2.45)	0.68	0.73
Multi-node	0.86(0.39-1.87)	0.7		3.81(1.24-11.72)	0.02	
Multi-size	2.24(1.13-4.44)	0.02		2.07(0.82-5.18)	0.12	

	HR(CI)	p-value	conc	HR(CI)	p-value	conc
Uni-CXCL13	0.21(0.1-0.46)	1.50E-05	0.69	0.76(0.28-2.1)	0.6	0.545
Multi-CXCL13	0.24(0.11-0.54)	0.00045	0.721	0.83(0.29-2.37)	0.73	0.739
Multi-node	0.69(0.32-1.49)	0.35		3.61(1.22-10.71)	0.021	
Multi-size	1.71(0.83-3.55)	0.15		2.12(0.86-5.22)	0.1	

Table S2. Univariate and multivariate Cox regression results for ITL and other eight signatures using the optimal cut-offs selected in Cohort 2 and validated in Cohort 1. Uni-: Univariate Cox regression; Multi-: Multivariate Cox regression; HR: Hazard Ratio; CI: lower and higher 95% Confidence Interval; Conc: Concordance.

	**Cohort1**			**Cohort2**		
	HR(CI)	p-value	conc	HR(CI)	p-value	conc
Uni-ITL	0.45(0.21-0.96)	0.033	0.587	0.26(0.1-0.71)	0.0048	0.656
Multi-ITL	0.38(0.17-0.84)	0.016	0.654	0.16(0.05-0.48)	0.001	0.76
Multi-node	0.62(0.28-1.37)	0.23		4.64(1.52-14.15)	0.007	
Multi-size	2.62(1.27-5.39)	0.0088		2.07(0.89-4.83)	0.091	
	HR(CI)	p-value	conc	HR(CI)	p-value	conc
Uni-Lym	0.91(0.43-1.91)	0.8	0.524	0.35(0.13-0.98)	0.038	0.63
Multi-Lym	0.92(0.43-1.95)	0.82	0.627	0.29(0.1-0.85)	0.024	0.778
Multi-node	0.72(0.33-1.58)	0.41		3.82(1.29-11.38)	0.016	
Multi-size	2.33(1.18-4.64)	0.015		1.85(0.73-4.73)	0.2	
	HR(CI)	p-value	conc	HR(CI)	p-value	conc
Uni-Calabro	0.53(0.24-1.2)	0.12	0.578	0(0-Inf)	0.04	0.608
Multi-Calabro	0.56(0.24-1.28)	0.17	0.667	0(0-Inf)	1	0.799
Multi-node	0.67(0.31-1.46)	0.31		4.12(1.39-12.23)	0.011	
Multi-size	2.17(1.11-4.24)	0.023		1.91(0.8-4.55)	0.14	
	HR(CI)	p-value	conc	HR(CI)	p-value	conc
Uni-IL8	1.76(0.86-3.6)	0.12	0.575	0.18(0.05-0.63)	0.0026	0.692
Multi-IL8	1.74(0.84-3.59)	0.14	0.65	0.21(0.06-0.77)	0.018	0.795
Multi-node	0.8(0.37-1.73)	0.57		3.76(1.25-11.34)	0.019	
Multi-size	2.29(1.17-4.51)	0.016		1.87(0.68-5.17)	0.23	
	HR(CI)	p-value	conc	HR(CI)	p-value	conc
Uni-Bcell	0.74(0.35-1.59)	0.44	0.541	0.41(0.09-1.82)	0.23	0.557
Multi-Bcell	0.75(0.35-1.6)	0.45	0.63	0.33(0.07-1.5)	0.15	0.763
Multi-node	0.72(0.33-1.55)	0.39		4.24(1.41-12.76)	0.01	
Multi-size	2.33(1.17-4.61)	0.016		1.94(0.81-4.67)	0.14	
	HR(CI)	p-value	conc	HR(CI)	p-value	conc
Uni-Bcell.IL8	0.82(0.35-1.92)	0.65	0.511	0.45(0.16-1.31)	0.13	0.599
Multi-Bcell.IL8	0.75(0.32-1.76)	0.51	0.629	0.59(0.2-1.76)	0.34	0.753
Multi-node	0.73(0.34-1.59)	0.43		3.28(1.08-9.94)	0.036	
Multi-size	2.4(1.2-4.83)	0.014		2.18(0.85-5.6)	0.11	
	HR(CI)	p-value	conc	HR(CI)	p-value	conc
Uni-Ascierto	0.83(0.39-1.78)	0.64	0.52	2.26(0.82-6.23)	0.11	0.63
Multi-Ascierto	1(0.46-2.16)	0.99	0.619	2.6(0.87-7.82)	0.089	0.773
Multi-node	0.73(0.34-1.59)	0.43		3.22(1.08-9.61)	0.036	
Multi-size	2.33(1.16-4.66)	0.017		2.46(0.91-6.62)	0.075	
	HR(CI)	p-value	conc	HR(CI)	p-value	conc
Uni-CXCR3	0.56(0.24-1.29)	0.17	0.569	0(0-Inf)	0.029	0.618

Multi-CXCR3	0.62(0.26-1.48)	0.28	0.658	0(0-Inf)	1	0.805
Multi-node	0.68(0.32-1.49)	0.34		4.17(1.41-12.38)	0.01	
Multi-size	2.16(1.09-4.28)	0.028		1.89(0.8-4.46)	0.15	
	HR(CI)	p-value	conc	HR(CI)	p-value	conc
Uni-CXCL13	0.35(0.17-0.75)	0.0043	0.605	2.21(0.71-6.86)	0.16	0.595
Multi-CXCL13	0.38(0.18-0.79)	0.01	0.663	3.38(0.92-12.45)	0.067	0.773
Multi-node	0.69(0.32-1.49)	0.35		3.71(1.24-11.1)	0.019	
Multi-size	2.29(1.11-4.72)	0.026		2.71(0.94-7.8)	0.064	

Table S3 Top 20 genes positively correlated with ITL and top 10 genes negatively correlated with ITL (grey).

Symbol	Cytoband	Description	cor	q
SH3KBP1	Xp22.12b	SH3-domain kinase binding protein 1	0.4	0.0011
FCRL3	1q23.1d	Fc receptor-like 3	0.4	0.0011
LCK	1p35.1b	lymphocyte-specific protein tyrosine kinase	0.4	0.0011
GPR18	13q32.3a	G protein-coupled receptor 18	0.39	0.0011
TNFRSF13 B	17p11.2h	tumour necrosis factor receptor superfamily, member 13B	0.39	0.0011
SEMA4D/ CD100	9q22.2a	sema domain, immunoglobulin domain (Ig), transmembrane domain (TM) and short cytoplasmic domain, (semaphorin) 4D	0.39	0.0012
MAP4K1	19q13.2a	mitogen-activated protein kinase kinase kinase kinase 1	0.39	0.0012
RLTPR	16q22.1b	RGD motif, leucine rich repeats, tropomodulin domain and proline-rich containing	0.38	0.0012
UBASH3A	21q22.3b	ubiquitin associated and SH3 domain containing A	0.38	0.0012
IKZF3	17q12c	IKAROS family zinc finger 3 (Aiolos)	0.38	0.0012
CYFIP2	5q33.3a-q33.3b	cytoplasmic FMR1 interacting protein 2	0.38	0.0012
CXCR3	Xq13.1d	chemokine (C-X-C motif) receptor 3	0.38	0.0012
CD3E	11q23.3d	CD3e molecule, epsilon (CD3-TCR complex)	0.38	0.0012
IL2RG	Xq13.1c	interleukin 2 receptor, gamma	0.38	0.0012
CXCR5	11q23.3e	chemokine (C-X-C motif) receptor 5	0.38	0.0014
CTSW	11q13.1d	cathepsin W	0.37	0.0018
SH2D1A	Xq25c	SH2 domain containing 1A	0.37	0.0018
SEPT6	Xq24c	septin 6	0.37	0.0018
CTLA4	2q33.2a	cytotoxic T-lymphocyte-associated protein 4	0.37	0.0019
SIRPG	20p13e	signal-regulatory protein gamma	0.37	0.0019
C10orf141	10q26.2b		-0.4	0.0011
CD151	11p15.5c	CD151 molecule (Raph blood group)	-0.39	0.0011
SPP1	4q22.1b	secreted phosphoprotein 1	-0.39	0.0012
ANXA2	15q22.2a	annexin A2	-0.39	0.0012
P4HA2	5q31.1b	prolyl 4-hydroxylase, alpha polypeptide II	-0.36	0.0022
MUSK	9q31.3b	muscle, skeletal, receptor tyrosine kinase	-0.36	0.0023
POFUT2	21q22.3e	protein O-fucosyltransferase 2	-0.36	0.0025
ITGB5	3q21.2a	integrin, beta 5	-0.35	0.004
MXRA7	17q25.1d-q25.2a	matrix-remodelling associated 7	-0.34	0.0046
CALN1	7q11.22c	calneuron 1	-0.34	0.0046

Table S4. Full correlation table of ITL-association with gene expression (attached file).

Table S5. Top five MSigDB immunologic signatures enriched in genes positively/negatively (grey) correlated with ITL with the adjusted p value, gene set names, and gene symbols.

p	Gene Set Term	Genes
$3x10^{-46}$	GSE22886_NAIVE_CD8_TCELL_VS_MONOCYTE_UP	*GPR18,LCK,UBASH3A,CXCR3,CD3E,SH2D1A,SIRPG,LIME1,BACH2,CD27,PVRIG,CD3G,PTPRCAP,CD7,ZAP70,CD247,PASK,IL18R1,CD5,CD8A,ITK,CD2,NKG7,P2RY10,CD6,CD3D,SPOCK2,MAL,NELL2,PTPN4,ETS1,SKAP1,LEF1,GZMK,LY9,IL18RAP,RAPGEF6,CD160,GNLY,SP140,KLRC3,PRF1,ZBTB25,IL21R,FAIM3,BCL11B,DENND2D,CD8B,TBC1D4*

8x10^{-40}	GSE10325_LUPUS_CD4_TCELL_VS_LUPUS_MYELOID_UP	*LCK,UBASH3A,IL2RG,SEPT6,BACH2,CD27,CD3G,PTPRCAP,ZAP70,CD247,PASK,ZCCHC11,TNFRSF25,SLAMF1,ITK,CD2,CD6,PRKD2,CD3D,SPOCK2,RHOF,TXK,MAL,ICOS,NELL2,PTPN4,PPP1R16B,SKAP1,LEF1,BCL2,LY9,RAPGEF6,STAT4,IL16,ZBTB25,TCF7,FAIM3,BCL11B,RASGRP1,GOLGA8A,DENND2D,ATF7IP2,TBC1D4,NCL*
3x10^{-35}	GSE29618_BCELL_VS_MONOCYTE_DAY7_FLU_VACCINE_UP	*GPR18,TNFRSF13B,MAP4K1,IKZF3,CYFIP2,IL2RG,SEPT6,BACH2,POU2AF1,PTPRCAP,CD19,BTG1,CD79A,MS4A1,TCL1A,STAP1,BANK1,BLK,P2RY10,CCR6,HLADOB,ADAM28,SPOCK2,CD79B,ETS1,PPP1R16B,SKAP1,FCER2,RAPGEF6,CD22,SP140,BCL11A,BIRC3,NSUN5,ADD3,PNOC,FAIM3,GSDMB,RASGRP1,GOLGA8A*
4x10^{-35}	GSE22886_NAIVE_CD4_TCELL_VS_MONOCYTE_UP	*GPR18,LCK,UBASH3A,CD3E,SH2D1A,SIRPG,LIME1,BACH2,CD27,PVRIG,CD3G,PTPRCAP,CD7,ZAP70,TRAT1,CD247,TRD@,PASK,CD5,ITK,P2RY10,CD6,CD3D,SPOCK2,TXK,MAL,ICOS,NELL2,CD40LG,PTPN4,ETS1,SKAP1,LEF1,BCL2,LY9,ZBTB25,FAIM3,BCL11B,GSDMB,DENND2D,TBC1D4*
2x10^{-31}	GSE10325_BCELL_VS_MYELOID_UP	*GPR18,MAP4K1,CXCR5,SEPT6,BACH2,CCR7,CD27,POU2AF1,PTPRCAP,CD19,CD79A,ZCCHC11,SLAMF1,CR2,MS4A1,TCL1A,STAP1,BANK1,CCR6,PRKD2,HLADOB,FAM117A,CD79B,PPP1R16B,SKAP1,BCL2,LY9,FCER2,CD22,SP140,IL16,BCL11A,ITGB7,BIRC3,PNOC,FAIM3,RASGRP1*
4x10^{-9}	GSE24634_TREG_VS_TCONV_POST_DAY10_IL4_CONVERSION_DN	*SPP1,ITGB5,MYOF,ASPH,PALLD,RAB31,PMP22,LMNA,PLAU,FCGR2A,SCD,BACE1,LAMP2*
5 x10^{-5}	GSE3982_DC_VS_NKCELL_UP	*P4HA2,RAB23,ASPH,RAB31,ADM,FN1,SCD,ANXA2P1,CYB5R3*
4x10^{-5}	GSE7460_CTRL_VS_TGFB_TREATED_ACT_CD8_TCELL_UP	*ANXA2,ITGB5,NUPR1,LGALS1,RAB23,PLAUR,AHNAK,IER3,FSTL3*
1 x10^{-4}	GSE6269_HEALTHY_VS_STREP_AUREUS_INF_PBMC_DN	*RAB1A,ARF4,PLAUR,PPP1R15A,ADM,DUSP3,ADAM9,LAMP2*
3 x10^{-4}	GSE29618_BCELL_VS_MDC_DN	*ANXA2,LGALS1,GBE1,MYOF,RAB31,LMNA,DUSP3,SCD*

Table S6. Top five MSigDB canonical PID pathways enriched in genes positively / negatively (grey) correlated with ITL with the adjusted p value, gene set names, and gene symbols.

2 x10^{-14}	PID_CD8TCRPATHWAY	*LCK,CD3E,CD3G,ZAP70,CD247,CD8A,FYN,RASSF5,CD3D,RASGRP2,PRF1,PRKCB,RASGRP1,CD8B,LAT*
5 x10^{-14}	PID_IL12_2PATHWAY	*LCK,CD3E,IL2RG,CD3G,CD247,IL18R1,IFNG,CD8A,CD3D,EOMES,IL18RAP,STAT4,NFKB2,STAT1,CD8B*
3 x10^{-13}	PID_TCR_PATHWAY	*LCK,MAP4K1,CD3E,SLA2,CD3G,ZAP70,CD247,ITK,FYN,RASSF5,CD3D,RASGRP2,PRKCB,RASGRP1,LAT*
4x10^{-13}	PID_CD8TCRDOWNSTREAMPATHWAY	*CD3E,IL2RG,CD3G,CD247,IFNG,CD8A,PTPN7,CD3D,EOMES,STAT4,PRF1,NFATC3,TNFRSF4,PRKCB,CD8B*
3x10^{-10}	PID_IL12_STAT4PATHWAY	*CD3E,CD3G,CD247,IL18R1,IFNG,CD3D,IL18RAP,STAT4,CREBBP,PRF1*
2x10^{-4}	PID_UPA_UPAR_PATHWAY	*ITGB5,PLAUR,BCAR1,FN1,PLAU*
2x10^{-4}	PID_INTEGRIN5_PATHWAY	*ITGB5,PLAUR,FN1,PLAU*
1x10^{-3}	PID_INTEGRIN1_PATHWAY	*SPP1,THBS2,PLAUR,FN1,PLAU*
1x10^{-3}	PID_INTEGRIN_A4B1_PATHWAY	*SPP1,THBS2,BCAR1,FN1*
2x10^{-3}	PID_AVB3_INTEGRIN_PATHWAY	*SPP1,COL8A1,COL13A1,BCAR1,FN1*

Table S7. KEGG pathways enriched in ITL-associated genes (positive correlation) (attached file).

Table S8. Canonical pathways enriched in ITL-associated genes (positive correlation) (attached file).

Table S9. Immunologic pathways enriched in ITL-associated genes (positive correlation) (attached file).

Table S10. KEGG pathways enriched in ITL-associated genes (negative correlation) (attached file).

Table S11. Canonical pathways enriched in ITL-associated genes (negative correlation) (attached file).

Table S12. Immunologic pathways enriched in ITL-associated genes (negative correlation) (attached file).

Table S13. Genes in the CTLA4 and APOBEC3G modules. G: copy-number gains, L: losses in 181 tumours, Cor: correlation, *q*: FDR-adjusted q-value.

Symbol	Cyto	Description	G%	L%	Cor	*q*

NKG7	19q13.33d	natural killer cell group 7 sequence	2.2	1.7	0.32	0.01
IL18RAP	2q12.1a	interleukin 18 receptor accessory protein	2.2	1.7	0.29	0.019
M17016	14q12a		3.9	3.9	0.29	0.023
PRF1	10q22.1b	perforin 1 (pore forming protein)	5	1.1	0.28	0.029
APOBEC 3G	22q13.1c	apolipoprotein B mRNA editing enzyme, catalytic polypeptide-like 3G	5	2.2	0.28	0.03
IL21R	16p12.1a	interleukin 21 receptor	5	0	0.27	0.036
SEMA4D	9q22.2a	sema domain, immunoglobulin domain (Ig), transmembrane domain (TM) and short cytoplasmic domain, (semaphorin) 4D	5.5	1.1	0.39	0.0012
CTLA4	2q33.2a	cytotoxic T-lymphocyte-associated protein 4	3.9	1.1	0.37	0.0019
MCOLN2	1p22.3f	mucolipin 2	5	2.8	0.36	0.0028
CXCL13	4q21.1c	chemokine (C-X-C motif) ligand 13	2.8	1.1	0.35	0.0034
HLA-DOB	6p21.32a	major histocompatibility complex, class II, DO beta	9.4	1.7	0.31	0.012
TIGIT	3q13.31a	T cell immunoreceptor with Ig and ITIM domains	5	0.6	0.31	0.013
SPOCK2	10q22.1e	sparc/osteonectin, cwcv and kazal-like domains proteoglycan (testican) 2	7.2	1.7	0.31	0.014
ICOS	2q33.2a	inducible T-cell co-stimulator	3.9	1.1	0.3	0.016
ITGB7	12q13.13e	integrin, beta 7	1.7	2.2	0.28	0.029

Reference

1. Ruffell B, Au A, Rugo HS, Esserman LJ, Hwang ES, Coussens LM. Leukocyte composition of human breast cancer. Proceedings of the National Academy of Sciences of the United States of America. 2012;109(8):2796-801.
2. Fridman WH, Pages F, Sautes-Fridman C, Galon J. The immune contexture in human tumours: impact on clinical outcome. Nature reviews Cancer. 2012;12(4):298-306.
3. Andre F, Dieci MV, Dubsky P, Sotiriou C, Curigliano G, Denkert C, et al. Molecular pathways: involvement of immune pathways in the therapeutic response and outcome in breast cancer. Clinical cancer research : an official journal of the American Association for Cancer Research. 2013;19(1):28-33.
4. Klinke DJ, 2nd. Induction of Wnt-inducible signaling protein-1 correlates with invasive breast cancer oncogenesis and reduced type 1 cell-mediated cytotoxic immunity: a retrospective study. PLoS computational biology. 2014;10(1):e1003409.
5. Deng X, Li Q, Hoff J, Novak M, Yang H, Jin H, et al. Integrin-associated CD151 drives ErbB2-evoked mammary tumor onset and metastasis. Neoplasia. 2012;14(8):678-89.
6. Brichory FM, Misek DE, Yim AM, Krause MC, Giordano TJ, Beer DG, et al. An immune response manifested by the common occurrence of annexins I and II autoantibodies and high circulating levels of IL-6 in lung cancer. Proceedings of the National Academy of Sciences of the United States of America. 2001;98(17):9824-9.
7. Mach N, Gao Y, Lemonnier G, Lecardonnel J, Oswald IP, Estelle J, et al. The peripheral blood transcriptome reflects variations in immunity traits in swine: towards the identification of biomarkers. BMC genomics. 2013;14:894.
8. Takeda Y, Li Q, Kazarov AR, Epardaud M, Elpek K, Turley SJ, et al. Diminished metastasis in tetraspanin CD151-knockout mice. Blood. 2011;118(2):464-72.
9. Kiyokawa E, Hashimoto Y, Kobayashi S, Sugimura H, Kurata T, Matsuda M. Activation of Rac1 by a Crk SH3-binding protein, DOCK180. Genes & development. 1998;12(21):3331-6.
10. Wang KX, Denhardt DT. Osteopontin: role in immune regulation and stress responses. Cytokine & growth factor reviews. 2008;19(5-6):333-45.
11. Salvi S, Fontana V, Boccardo S, Merlo DF, Margallo E, Laurent S, et al. Evaluation of CTLA-4 expression and relevance as a novel prognostic factor in patients with non-small cell lung cancer. Cancer immunology, immunotherapy : CII. 2012;61(9):1463-72.
12. Schalper KA, Velcheti V, Carvajal D, Wimberly H, Brown J, Pusztai L, et al. In Situ Tumor PD-L1 mRNA Expression Is Associated with Increased TILs and Better Outcome in Breast Carcinomas. Clinical cancer research : an official journal of the American Association for Cancer Research. 2014.
13. Kuong KJ, Loeb LA. APOBEC3B mutagenesis in cancer. Nature genetics. 2013;45(9):964-5.

14. Monajemi M, Woodworth CF, Benkaroun J, Grant M, Larijani M. Emerging complexities of APOBEC3G action on immunity and viral fitness during HIV infection and treatment. Retrovirology. 2012;9:35.
15. Castro MA, Wang X, Fletcher MN, Meyer KB, Markowetz F. RedeR: R/Bioconductor package for representing modular structures, nested networks and multiple levels of hierarchical associations. Genome biology. 2012;13(4):R29.
16. Jaatinen T, Hemmoranta H, Hautaniemi S, Niemi J, Nicorici D, Laine J, et al. Global gene expression profile of human cord blood-derived CD133+ cells. Stem Cells. 2006;24(3):631-41.
17. Grabarczyk P, Przybylski GK, Depke M, Volker U, Bahr J, Assmus K, et al. Inhibition of BCL11B expression leads to apoptosis of malignant but not normal mature T cells. Oncogene. 2007;26(26):3797-810.
18. Abbas AR, Baldwin D, Ma Y, Ouyang W, Gurney A, Martin F, et al. Immune response in silico (IRIS): immune-specific genes identified from a compendium of microarray expression data. Genes and immunity. 2005;6(4):319-31.
19. Hutcheson J, Scatizzi JC, Siddiqui AM, Haines GK, 3rd, Wu T, Li QZ, et al. Combined deficiency of proapoptotic regulators Bim and Fas results in the early onset of systemic autoimmunity. Immunity. 2008;28(2):206-17.
20. Pilon AM, Ajay SS, Kumar SA, Steiner LA, Cherukuri PF, Wincovitch S, et al. Genome-wide ChIP-Seq reveals a dramatic shift in the binding of the transcription factor erythroid Kruppel-like factor during erythrocyte differentiation. Blood. 2011;118(17):e139-48.
21. Anastassiou D, Rumjantseva V, Cheng W, Huang J, Canoll PD, Yamashiro DJ, et al. Human cancer cells express Slug-based epithelial-mesenchymal transition gene expression signature obtained in vivo. BMC cancer. 2011;11:529.
22. Bronger H, Kraeft S, Schwarz-Boeger U, Cerny C, Stockel A, Avril S, et al. Modulation of CXCR3 ligand secretion by prostaglandin E2 and cyclooxygenase inhibitors in human breast cancer. Breast cancer research : BCR. 2012;14(1):R30.
23. Liu M, Guo S, Stiles JK. The emerging role of CXCL10 in cancer (Review). Oncology letters. 2011;2(4):583-9.
24. Mold JE, Venkatasubrahmanyam S, Burt TD, Michaelsson J, Rivera JM, Galkina SA, et al. Fetal and adult hematopoietic stem cells give rise to distinct T cell lineages in humans. Science. 2010;330(6011):1695-9.
25. Duraiswamy J, Ibegbu CC, Masopust D, Miller JD, Araki K, Doho GH, et al. Phenotype, function, and gene expression profiles of programmed death-1(hi) CD8 T cells in healthy human adults. J Immunol. 2011;186(7):4200-12.
26. Swainson LA, Mold JE, Bajpai UD, McCune JM. Expression of the autoimmune susceptibility gene FcRL3 on human regulatory T cells is associated with dysfunction and high levels of programmed cell death-1. J Immunol. 2010;184(7):3639-47.
27. Perou C, Sorlie T, Eisen M, van de Rijn M, Jeffrey S, Rees C, et al. Molecular portraits of human breast tumours. Nature. 2000;406:747-52.
