# Supplementary material for: Modelling the spatial heterogeneity and molecular correlates of lymphocytic infiltration in triple-negative breast cancer
Source: J R Soc Interface. 2015 Feb 6;12(103):20141153. doi: 10.1098/rsif.2014.1153 (PMC4305416; doi:10.1098/rsif.2014.1153)
Supplement: Sweave file [file rsif20141153supp2.pdf]

Supplementary materials, R code and data for – Modelling the Spatial Heterogeneity and Molecular Correlates of Lymphocytic Infiltration in Triple-Negative Breast Cancer–

Contents

1 Preparation **2**
- 1.1 Load data and functions 2
- 1.2 Clinical parameters of the TNBC samples 2
- 1.3 Clinical parameters and ITLR 3
- 1.4 ITLR and other immune signatures 6

2 Association between ITLR and TNBC prognosis **7**
- 2.1 Prognostic value of ITLR 7
- 2.2 Additional value to standard clinical parameters 10
- 2.3 Comparisons of immune signatures 12
- 2.4 Robustness of Cox model 16

3 Generation of ITLR data step-by-step **18**
- 3.1 Image data 18
- 3.2 Identify the optimal bandwidth for computing cancer density 18
- 3.3 Generate spatial proximity to cancer for each lymphocyte 19
- 3.4 Identify sub-populations of lymphocyte by unsupervised learning 20
- 3.5 Clustering stability 21

4 Differences of the three lymphocyte clusters **23**
- 4.1 Distance to nearest cancer cell 23
- 4.2 Distance to cancer convex hull 24
- 4.3 Differences in 10,000 randomly sampled lymphocytes 28

5 Generating ITLR in TNBCs **31**
- 5.1 Illustration of ITLs with a sample slide image 31
- 5.2 Generating ITLR, ATLR, and DTLR 33

6 Compare ITLR, ATLR, and DTLR **35**

7 Association with molecular profiling **38**

8 Session Info **39**

1 Preparation

1.1 Load data and functions

To reproduce our result, first source the R functions needed for analysis and plotting and load the data for the 181 triple negative breast cancer samples (TNBC), which is available as part of the paper supplement.

```
library(survival)
load("./data/ProcessedDataAndTrait.rdata")
source("functions.R")
```

1.2 Clinical parameters of the TNBC samples

Each row in this clinical file is a sample/patient from the METABRIC database. Columns indicate: public METABRIC ID (public), 10-year disease-specific survival (S_10year), Pam50 intrinsic subtype classification (Pam50Subtype), sample subsets (Site), proportions of lymphocytes, cancer, and stromal cells, and immune expression signature from microarray (LIscore), pathological scores of lymphocytic infiltration (LIpathological), lymph node status (node), tumour size (size), clinical grade (grade). The Site object describes the contributing hospitals and will be used to annotate samples from two cohorts, as described in the paper.

```
# Show clinical information data
head(trait)

##              public  file S_10year Pam50Subtype Site     lym
## MB-GU-1154 MB-7154 12975  108.47+        Basal    4 0.29168
## MB-GU-1155 MB-7155 12976  120.00+        Basal    4 0.33086
## MB-GU-1158 MB-7158 12979   46.43         Basal    4 0.28464
## MB-GU-1159 MB-7159 12983   21.93         Basal    4 0.24819
## MB-GU-1165 MB-7165 12986  120.00+        Basal    4 0.09422
## MB-GU-1114 MB-7114 13104  120.00+        Basal    4 0.07907
##            nTumour  LIscore LIpathological size node grade
## MB-GU-1154  102970  0.77004           <NA>    2    0     3
## MB-GU-1155  163827  0.60681           <NA>    1    1     3
## MB-GU-1158   68691  1.90112           <NA>    2    1     3
## MB-GU-1159   40968 -0.98905           <NA>    2    0     3
## MB-GU-1165  216611 -0.01292           <NA>    3    1     3
## MB-GU-1114   73500  0.65069           <NA>    1    0     3
##            TP53 Coding.description Codon.change
## MB-GU-1154    1           c.818G>A      CGT>CAT
## MB-GU-1155    1          c.723del1       TCC>NA
## MB-GU-1158    0
## MB-GU-1159    1      c.305_306del2       ACC>NA
## MB-GU-1165    1           c.524G>A      CGC>CAC
## MB-GU-1114    1          c.723del1       TCC>NA
##            Protein.change TP53IHC
## MB-GU-1154        p.R273H      NA
## MB-GU-1155            p.?      NA
## MB-GU-1158                     NA
## MB-GU-1159            p.?      NA
## MB-GU-1165        p.R175H      NA
## MB-GU-1114            p.?      NA

Site <- list(Site1=trait$Site==2 | trait$Site==4, Site2=trait$Site==1)
```

The summary statistics of breast tumours in our cohort are given below. We focus on disease-specific survival (DSS) within 10 years from diagnosis.

```
summary(trait$S_10year)

##       time             status     
##  Min.   :  0.27   Min.   :0.000  
##  1st Qu.: 32.01   1st Qu.:0.000  
##  Median : 62.73   Median :0.000  
##  Mean   : 66.32   Mean   :0.256  
##  3rd Qu.:108.79   3rd Qu.:1.000  
##  Max.   :120.00   Max.   :1.000  
##  NA's   :1        NA's   :1
```

Now the estimated median follow-up time can be calculated by the reverse Kaplan-Meier method. We invert the censoring index for death to estimate time to loss of follow up.

```
survfit(Surv(trait$S_10year[,1], trait$S_10year[,2]==0) ~ 1)

## Call: survfit(formula = Surv(trait$S_10year[, 1], trait$S_10year[, 
##     2] == 0) ~ 1)
## 
##    2 observations deleted due to missingness 
## records   n.max n.start  events  median 0.95LCL 0.95UCL 
##   179.0   179.0   179.0   133.0    79.5    69.9   102.0
```

There are 2 censoring events for DSS, and median DSS (shown earlier) will closely approximate median follow up.

1.3 Clinical parameters and ITLR

Distribution of ITLR scores in the two cohorts are visualised.

```
par(mfrow=c(1,2))
hist(mat[Site[[1]],1], br=30, xlim=c(0,0.3), main='Cohort 1', xlab='ITLR')
hist(mat[Site[[2]],1], br=30, xlim=c(0,0.3), main='Cohort 2', xlab='ITLR')
```

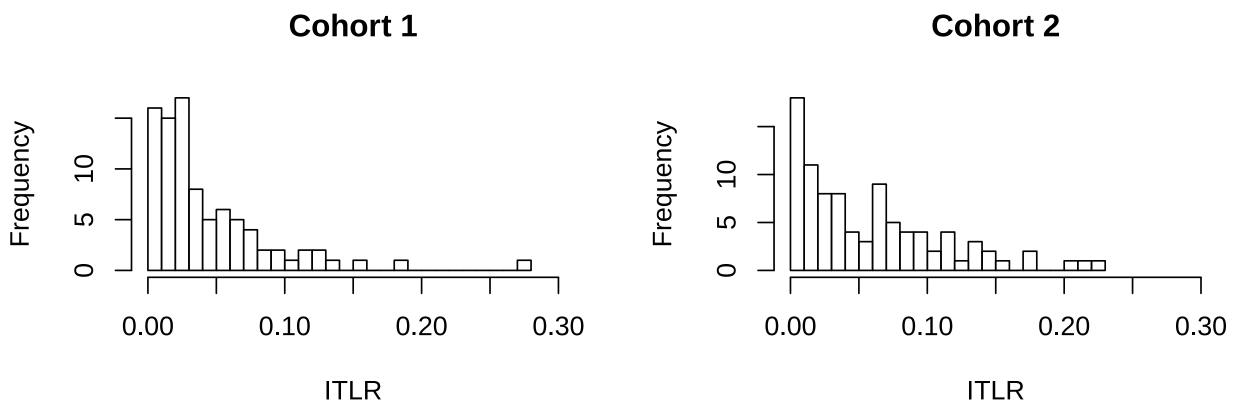

Prognostic values of other known clinical parameters in our TNBC cohorts can be visualised with Kaplan-Meier curves. Intrinsic subtype make up for our TNBC samples are 142 Basal, 20 Her2 and 19 Normal. And there is no significant differences in prognosis among these subtypes.

```
par(mfrow=c(2,3))
plotSurv(trait$S_10year, trait$Pam50Subtype, name='PAM50')
plotSurv(trait$S_10year, trait$LIpathological, name='Pathological LI')
plotSurv(trait$S_10year, trait$size, name='size')
plotSurv(trait$S_10year, trait$node, name='node')
plotSurv(trait$S_10year, trait$grade, name='Grade')
plotSurv(trait$S_10year, trait$TP53, name='TP53')
```

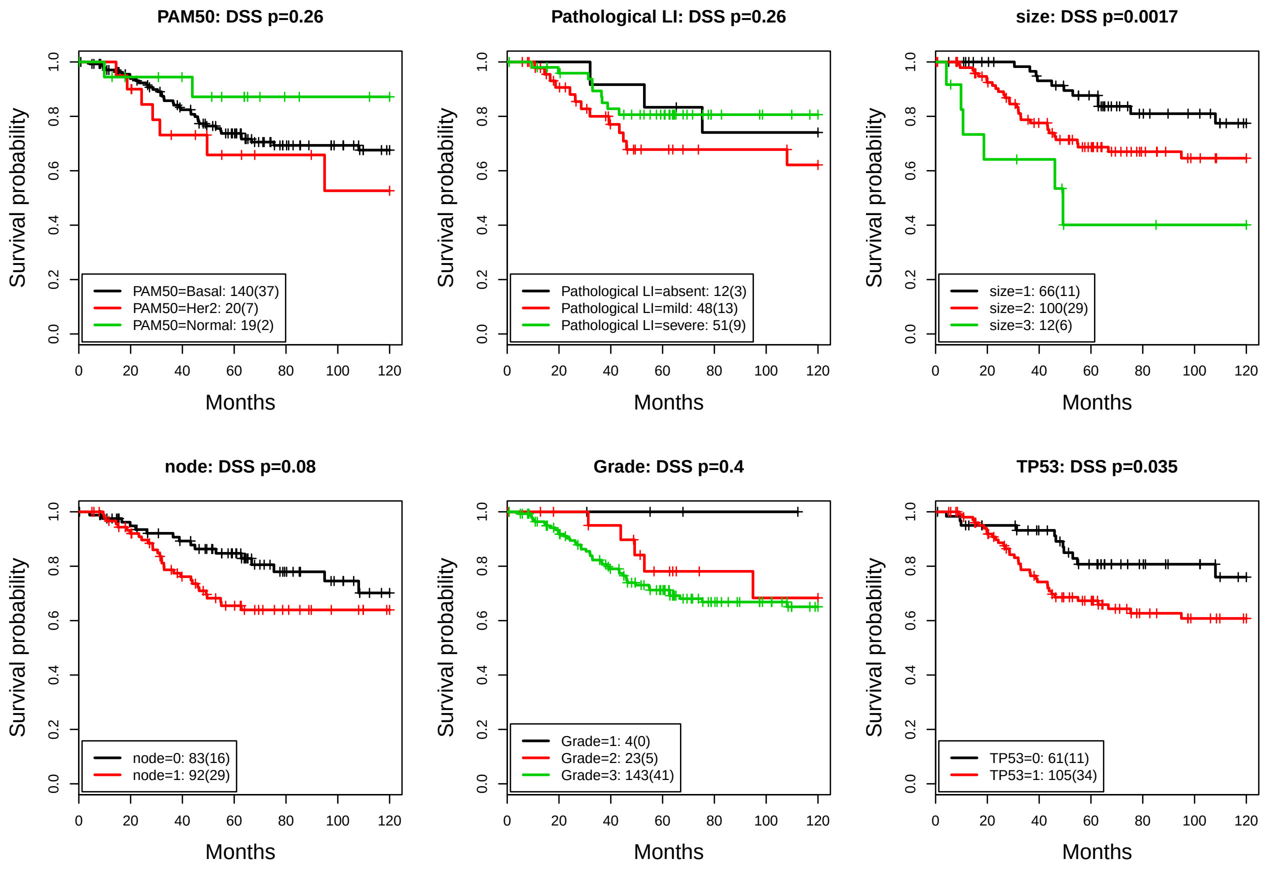

And from this box plot we can see, correlation between ITLR and pathological scores is significant using the JT trend test.

```
library(SAGx)
JT.test(data=mat[,1],class=trait$LIpathological)$p.value

## class was not an ordered factor.  Redefined to be one.
## [1] 2.394e-33

bpC<- boxplot(split(mat[,1],trait$LIpathological),horizontal=TRUE,las=1,notch=F,varwidth=TRUE,

## class was not an ordered factor.  Redefined to be one.

text(x=.25, y=1:3, paste('n =',bpC$n))
```

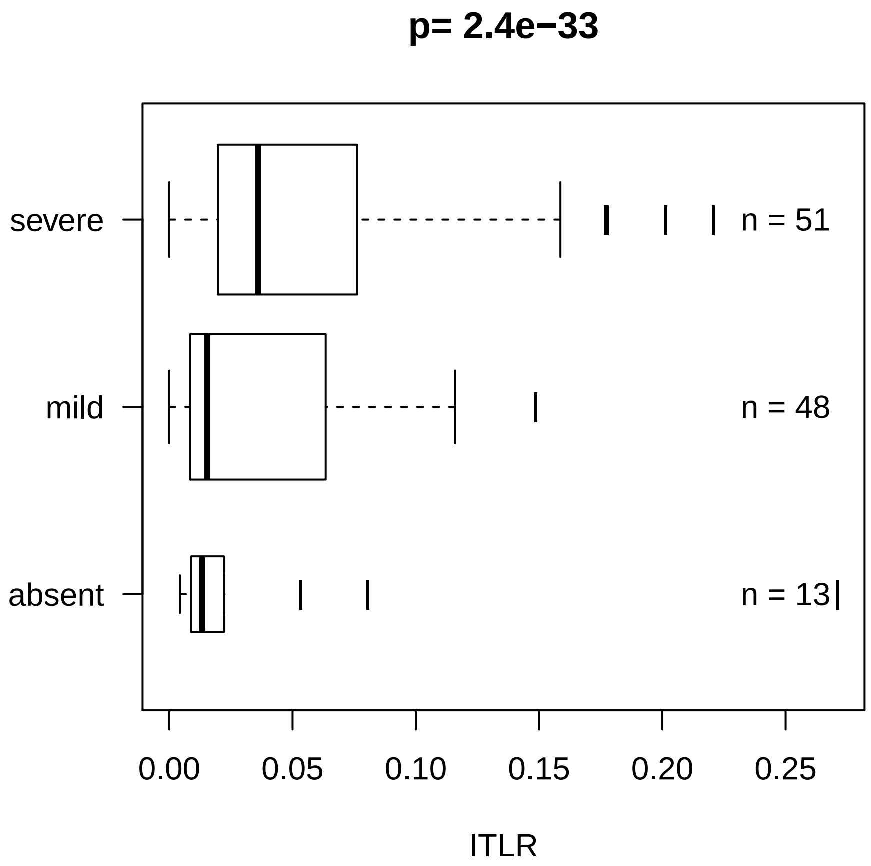

Correlation between ITL and the main clinical parameters can be shown in box plots:

```
par(mfrow=c(2,2), mar=c(4,4,3,1))
p1 <- JT.test(data=mat[,1],class=trait$size)$p.value

## class was not an ordered factor.  Redefined to be one.

bpC<- boxplot(split(mat[,1],trait$size),horizontal=TRUE,las=1,notch=F,varwidth=TRUE,xlab="ITLR
text(x=.25, y=1:3, paste('n =',bpC$n))
p1 <- JT.test(data=mat[,1],class=trait$node)$p.value

## class was not an ordered factor.  Redefined to be one.

bpC<- boxplot(split(mat[,1],trait$node),horizontal=TRUE,las=1,notch=F,varwidth=TRUE,xlab="ITLR
text(x=.25, y=1:3, paste('n =',bpC$n))
p1 <- JT.test(data=mat[,1],class=trait$grade)$p.value

## class was not an ordered factor.  Redefined to be one.

bpC<- boxplot(split(mat[,1],trait$grade),horizontal=TRUE,las=1,notch=F,varwidth=TRUE,xlab="ITL
text(x=.25, y=1:3, paste('n =',bpC$n))
p1 <- JT.test(data=mat[,1],class=trait$TP53)$p.value

## class was not an ordered factor.  Redefined to be one.

bpC<- boxplot(split(mat[,1],trait$TP53),horizontal=TRUE,las=1,notch=F,varwidth=TRUE,xlab="ITLR
text(x=.25, y=1:3, paste('n =',bpC$n))
```

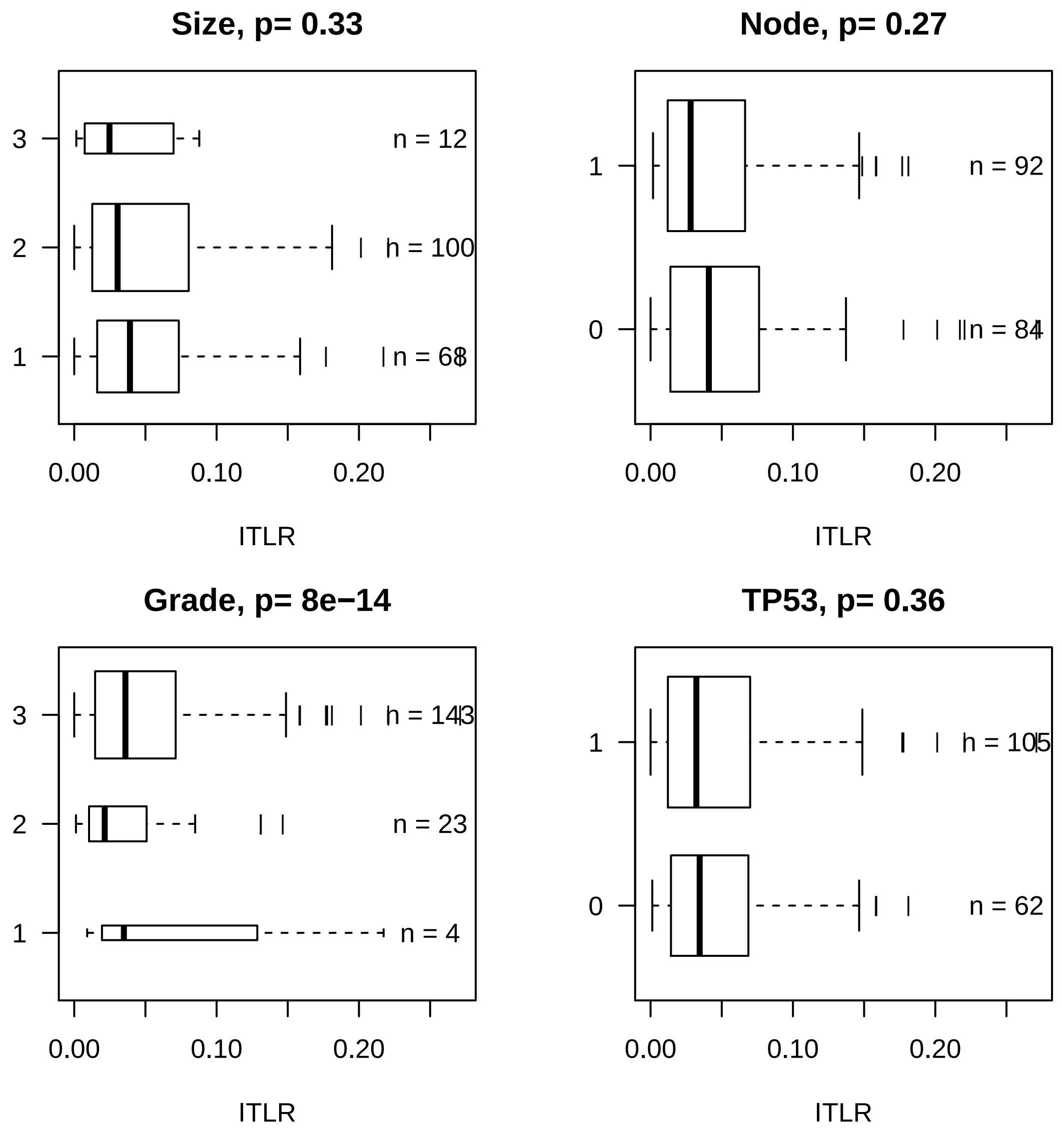

1.4 ITLR and other immune signatures

The "mat" object is a matrix with nine columns corresponding to nine immune signatures.

```
head(mat)

##           ITL     Lym  Calabro      IL8   Bcell Bcell.IL8
## 12975 0.10805 0.48207  0.77004 -0.20771  0.2179   -1.0493
## 12976 0.12844 0.51753  0.60681 -0.51593 -0.1224    0.2372
## 12979 0.15826 0.43428  1.90112  2.61573  2.1184    0.8099
## 12983 0.08604 0.35906 -0.98905 -0.21884 -0.7329    3.3492
## 12986 0.06867 0.11111 -0.01292  0.37603  0.6541    1.7396
## 13104 0.05041 0.09743  0.65069  0.04772  1.6124   33.7862
##       Ascierto   CXCR3  CXCL13
## 12975   2.1131  1.8384  1.8678
## 12976   0.7602  1.0008  0.7566
## 12979   0.8635  1.8529  2.0297
## 12983  -0.7160 -0.9659 -0.8178
## 12986   1.6863  0.2646  0.8920
## 13104   1.2826  0.7351  0.8042
```

2 Association between ITLR and TNBC prognosis

2.1 Prognostic value of ITLR

Using Site 1 as the discovery cohort and Site 2 as the validation cohort, we will test the association between prognosis and ITLR. i is set to 1 to refer to the ITLR score in the matrix mat. We search a range of quantiles from 20% to 80% for the optimal cut-off in the discovery cohort.

```
s <- 1
i <- 1
testrange=seq(0.20, 0.80,len=41)
library(survival)
p <- sapply(testrange, function(q){
  dat <- data.frame(x=mat[Site[[s]],i]>quantile(mat[Site[[s]],i], q), S=trait$S_10year[Site[[s
  fit <- survfit(S ~ x,data=dat)
  test <- survdiff(S ~ x, data=dat, rho=0)
  p.val <- 1 - pchisq(test$chisq, length(test$n) - 1)
  p.val})
```

Now plot the p-values from the log-rank test across different quantiles.

```
plot(testrange, -log(p), pch=19, xlab='Quantile range')
abline(h=-log(0.05), lty=2)
q <- testrange[which.min(p)]
th <- quantile(mat[Site[[s]],i], q)
th_ITL <- th
abline(v=q)
```

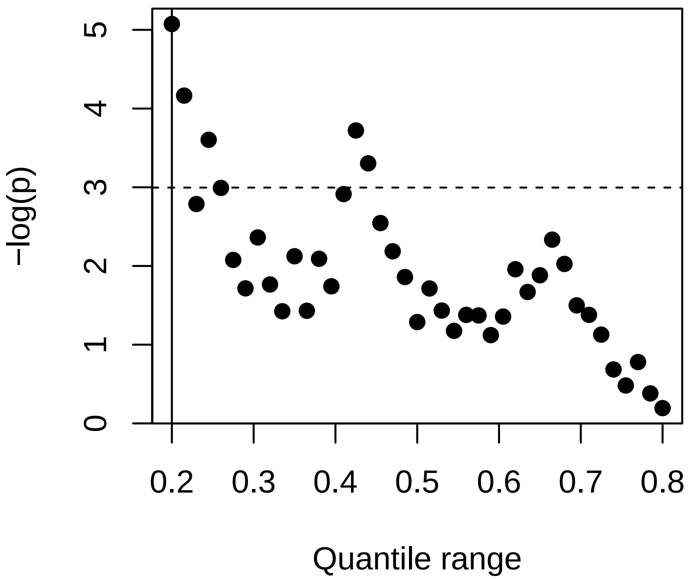

A cut-off of 0.011 at 20% quantile is identified as the optimal. Now we test this cut-off in both cohorts using KM curves to illustrate the result.

```
par(mfrow=c(1,2))
for (j in 1:2){
```

```
  tmp <- replace.vector(mat[Site[[j]],i]>th, c(TRUE, FALSE), c('High', 'Low'))
  try( plotSurv(trait$S_10year[Site[[j]],], tmp, fileType='', name=colnames(mat)[i]))
}
```

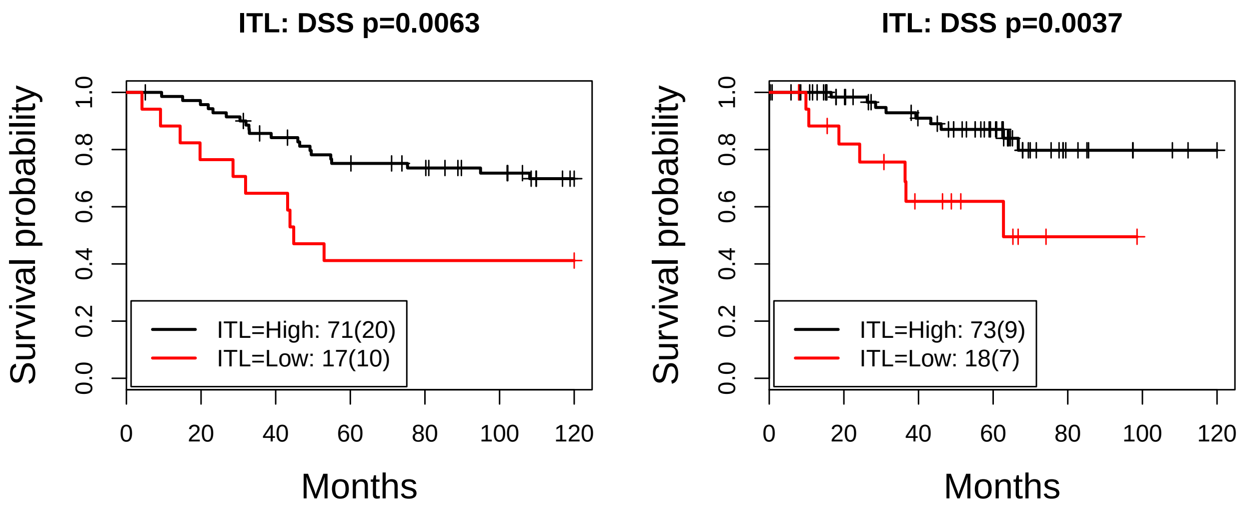

Overall, the survival probability for ITLR high and ITLR low groups are

```
summary(survfit(trait$S_10year[mat[,1]>th_ITL]~1))

## Call: survfit(formula = trait$S_10year[mat[, 1] > th_ITL] ~ 1)
##
## 1 observation deleted due to missingness
##     time n.risk n.event survival std.err lower 95% CI
##     9.43    138       1    0.993 0.00722        0.979
##    15.07    133       1    0.985 0.01033        0.965
##    16.60    129       1    0.978 0.01276        0.953
##    19.83    126       1    0.970 0.01483        0.941
##    21.93    123       1    0.962 0.01668        0.930
##    23.20    121       1    0.954 0.01834        0.919
##    26.27    120       1    0.946 0.01983        0.908
##    26.77    118       1    0.938 0.02122        0.897
##    28.50    116       1    0.930 0.02253        0.887
##    30.43    115       1    0.922 0.02374        0.877
##    31.30    114       1    0.914 0.02487        0.866
##    32.03    112       1    0.906 0.02595        0.856
##    32.83    111       1    0.898 0.02697        0.846
##    32.93    110       1    0.889 0.02793        0.836
##    38.80    107       1    0.881 0.02888        0.826
##    39.30    106       1    0.873 0.02978        0.816
##    43.30    103       1    0.864 0.03067        0.806
##    45.93    101       1    0.856 0.03154        0.796
##    46.07    100       1    0.847 0.03237        0.786
##    46.43     99       1    0.839 0.03315        0.776
##    49.20     97       1    0.830 0.03392        0.766
##    49.53     94       1    0.821 0.03469        0.756
##    54.77     91       1    0.812 0.03546        0.745
##    55.00     90       1    0.803 0.03620        0.735
##    62.77     77       1    0.793 0.03720        0.723
##    66.73     69       1    0.781 0.03839        0.709
```

```
##   75.33     61       1    0.768 0.03984        0.694
##   94.90     47       1    0.752 0.04221        0.674
##  108.07     40       1    0.733 0.04515        0.650
##  upper 95% CI
##         1.000
##         1.000
##         1.000
##         0.999
##         0.995
##         0.991
##         0.986
##         0.981
##         0.975
##         0.970
##         0.964
##         0.958
##         0.952
##         0.946
##         0.940
##         0.933
##         0.927
##         0.920
##         0.913
##         0.906
##         0.899
##         0.892
##         0.885
##         0.877
##         0.869
##         0.860
##         0.851
##         0.839
##         0.827
```

```
summary(survfit(trait$S_10year[mat[,1]<=th_ITL]~1))
```

```
## Call: survfit(formula = trait$S_10year[mat[, 1] <= th_ITL] ~ 1)
##
## 1 observation deleted due to missingness
##   time n.risk n.event survival std.err lower 95% CI
##   4.17     35       1    0.971  0.0282        0.918
##   9.13     33       1    0.942  0.0398        0.867
##   9.83     32       1    0.913  0.0482        0.823
##  10.60     31       1    0.883  0.0549        0.782
##  14.40     30       1    0.854  0.0605        0.743
##  18.67     28       1    0.823  0.0656        0.704
##  19.73     27       1    0.793  0.0699        0.667
##  24.23     26       1    0.762  0.0735        0.631
##  28.57     25       1    0.732  0.0766        0.596
##  31.93     23       1    0.700  0.0796        0.560
##  36.40     22       1    0.668  0.0821        0.525
##  36.63     21       1    0.636  0.0842        0.491
##  43.20     19       1    0.603  0.0861        0.456
##  43.83     18       1    0.569  0.0876        0.421
```

```
##   44.83      17        1     0.536  0.0886         0.387
##   52.97      13        1     0.495  0.0909         0.345
##   62.77      12        1     0.453  0.0922         0.304
##   upper 95% CI
##          1.000
##          1.000
##          1.000
##          0.998
##          0.981
##          0.962
##          0.942
##          0.921
##          0.898
##          0.875
##          0.850
##          0.825
##          0.798
##          0.770
##          0.741
##          0.709
##          0.675
```

2.2 Additional value to standard clinical parameters

Additional value of ITLR to node and size in grade 3 samples is demonstrated here using univariate and multivariate Cox regression analysis in Cohort 1. We used the log-rank test result from univariate analysis and Wald test results from multivariate analysis because multivariate analysis doesn't report log-rank p-value for individual variables.

```
x<- mat[,1]>th_ITL
print("Cohort 1")

## [1] "Cohort 1"

set2 <- Site[[1]]
summary(coxph(trait$S_10year[set2,]~x[set2]))

## Call:
## coxph(formula = trait$S_10year[set2, ] ~ x[set2])
##
##   n= 88, number of events= 30
##    (1 observation deleted due to missingness)
##
##                coef exp(coef) se(coef)     z Pr(>|z|)
## x[set2]TRUE -1.018     0.361    0.388 -2.62   0.0088 **
## ---
## Signif. codes:
## 0 '***' 0.001 '**' 0.01 '*' 0.05 '.' 0.1 ' ' 1
##
##             exp(coef) exp(-coef) lower .95 upper .95
## x[set2]TRUE     0.361       2.77     0.169     0.774
##
## Concordance= 0.601  (se = 0.034 )
```

```
## Rsquare= 0.066   (max possible= 0.944 )
## Likelihood ratio test= 5.97  on 1 df,   p=0.0146
## Wald test            = 6.87  on 1 df,   p=0.00878
## Score (logrank) test = 7.48  on 1 df,   p=0.00625
```

```
summary(coxph(trait$S_10year[set2,]~x[set2]+trait$node[set2]+trait$size[set2]))
```

```
## Call:
## coxph(formula = trait$S_10year[set2, ] ~ x[set2] + trait$node[set2] +
##     trait$size[set2])
##
##   n= 88, number of events= 30
##    (1 observation deleted due to missingness)
##
##                     coef exp(coef) se(coef)     z Pr(>|z|)
## x[set2]TRUE      -1.139     0.320    0.398 -2.86   0.0042
## trait$node[set2] -0.458     0.632    0.404 -1.13   0.2565
## trait$size[set2]  0.964     2.621    0.370  2.60   0.0092
##
## x[set2]TRUE      **
## trait$node[set2]
## trait$size[set2] **
## ---
## Signif. codes:
## 0 '***' 0.001 '**' 0.01 '*' 0.05 '.' 0.1 ' ' 1
##
##                  exp(coef) exp(-coef) lower .95 upper .95
## x[set2]TRUE          0.320      3.123     0.147     0.699
## trait$node[set2]     0.632      1.581     0.287     1.396
## trait$size[set2]     2.621      0.381     1.269     5.413
##
## Concordance= 0.668  (se = 0.053 )
## Rsquare= 0.139   (max possible= 0.944 )
## Likelihood ratio test= 13.2  on 3 df,   p=0.00428
## Wald test            = 12.9  on 3 df,   p=0.00489
## Score (logrank) test = 14  on 3 df,   p=0.00289
```

Similarly for the other cohort, ITLR independently predicts DSS in addition to node and size.

```
x<- mat[,1]>th_ITL
set2 <- Site[[2]]
print("Cohort 2")
```

```
## [1] "Cohort 2"
```

```
summary(coxph(trait$S_10year[set2,]~x[set2]))
```

```
## Call:
## coxph(formula = trait$S_10year[set2, ] ~ x[set2])
##
##   n= 91, number of events= 16
##    (1 observation deleted due to missingness)
##
##                coef exp(coef) se(coef)    z Pr(>|z|)
```

```
## x[set2]TRUE -1.367     0.255    0.507 -2.7    0.007 **
## ---
## Signif. codes:
## 0 '***' 0.001 '**' 0.01 '*' 0.05 '.' 0.1 ' ' 1
##
##              exp(coef) exp(-coef) lower .95 upper .95
## x[set2]TRUE     0.255       3.92    0.0944     0.688
##
## Concordance= 0.659  (se = 0.051 )
## Rsquare= 0.069   (max possible= 0.757 )
## Likelihood ratio test= 6.46  on 1 df,   p=0.011
## Wald test            = 7.27  on 1 df,   p=0.00701
## Score (logrank) test = 8.45  on 1 df,   p=0.00365
```

```
summary(coxph(trait$S_10year[set2,]~x[set2]+trait$node[set2]+trait$size[set2]))
```

```
## Call:
## coxph(formula = trait$S_10year[set2, ] ~ x[set2] + trait$node[set2] +
##     trait$size[set2])
##
##   n= 86, number of events= 15
##    (6 observations deleted due to missingness)
##
##                     coef exp(coef) se(coef)     z Pr(>|z|)
## x[set2]TRUE      -1.917     0.147    0.552 -3.48  0.00051
## trait$node[set2]  1.595     4.927    0.571  2.79  0.00521
## trait$size[set2]  0.726     2.066    0.424  1.71  0.08706
##
## x[set2]TRUE      ***
## trait$node[set2] **
## trait$size[set2] .
## ---
## Signif. codes:
## 0 '***' 0.001 '**' 0.01 '*' 0.05 '.' 0.1 ' ' 1
##
##                  exp(coef) exp(-coef) lower .95 upper .95
## x[set2]TRUE          0.147      6.799    0.0499     0.434
## trait$node[set2]     4.927      0.203    1.6096    15.085
## trait$size[set2]     2.066      0.484    0.8998     4.744
##
## Concordance= 0.76  (se = 0.077 )
## Rsquare= 0.21   (max possible= 0.752 )
## Likelihood ratio test= 20.2  on 3 df,   p=0.000151
## Wald test            = 19.1  on 3 df,   p=0.000259
## Score (logrank) test = 23.4  on 3 df,   p=3.29e-05
```

2.3 Comparisons of immune signatures

To compare ITLR with the other immune signatures, we perform the same tests. First, optimise the cut-offs in the discovery cohorts and record them in the Th object. Then these cut-offs are used in producing KM plots in both cohorts.

```
Th <- NULL
par(mfrow=c(2,3))
for (i in 2:3){
p <- sapply(testrange, function(q){
  dat <- data.frame(x=mat[Site[[s]],i]>quantile(mat[Site[[s]],i], q), S=trait$S_10year[Site[[s
  fit <- survfit(S ~ x,data=dat)
  test <- survdiff(S ~ x, data=dat, rho=0)
  p.val <- 1 - pchisq(test$chisq, length(test$n) - 1)
  p.val})

plot(testrange, -log(p), pch=19, xlab='Quantile range', main=colnames(mat)[i])
abline(h=-log(0.05), lty=2)
q <- testrange[which.min(p)]
th <- quantile(mat[Site[[s]],i], q)
abline(v=q)
Th <- c(Th, th)

for (j in 1:2){
  tmp <- replace.vector(mat[Site[[j]],i]>th, c(TRUE, FALSE), c('High', 'Low'))
  try( plotSurv(trait$S_10year[Site[[j]],], tmp, fileType='', name=colnames(mat)[i]))
}
}
```

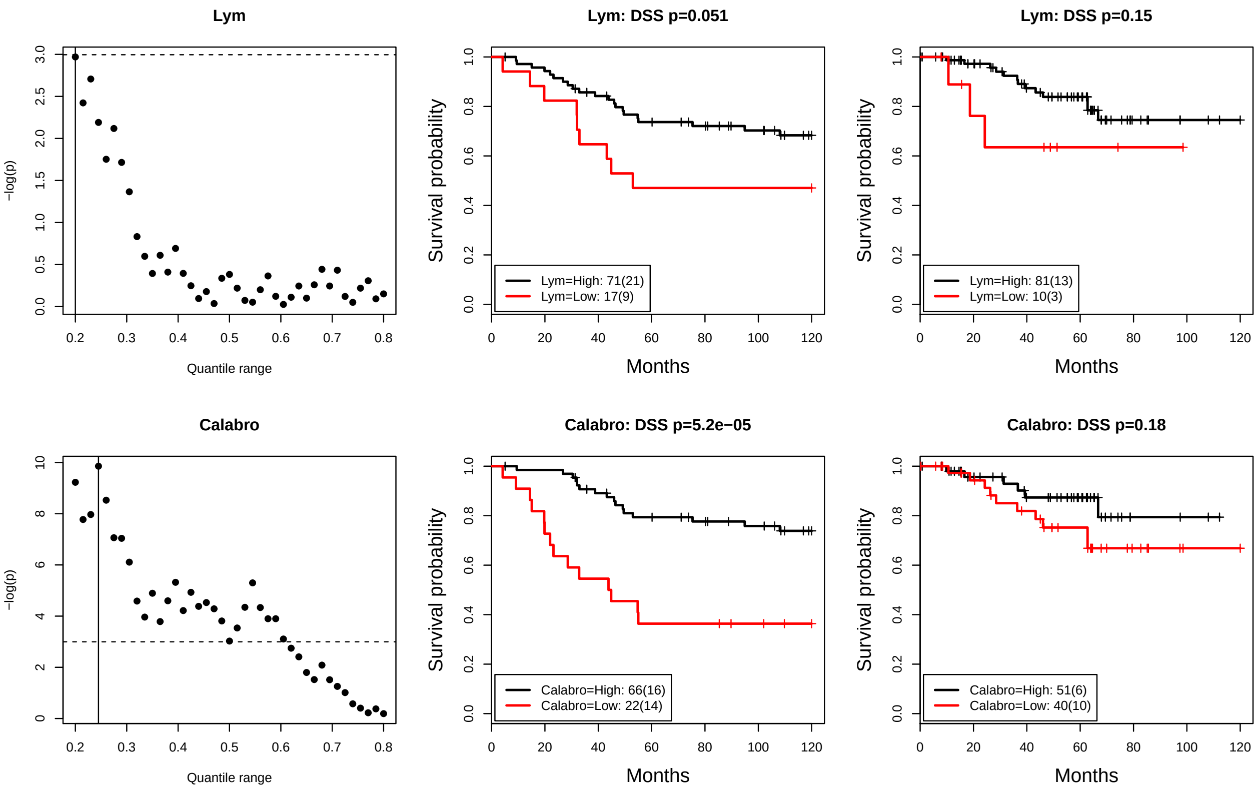

Also the same univariate and multivariate analysis for the other signatures.

```
i=2
print('Lym')

## [1] "Lym"

x<- mat[,i]>Th[i-1]
```

```r
set2 <- Site[[1]]
summary(coxph(trait$S_10year[set2,]~x[set2]))

## Call:
## coxph(formula = trait$S_10year[set2, ] ~ x[set2])
##
##   n= 88, number of events= 30
##    (1 observation deleted due to missingness)
##
##                coef exp(coef) se(coef)    z Pr(>|z|)
## x[set2]TRUE -0.759     0.468    0.399 -1.9    0.057 .
## ---
## Signif. codes:
## 0 '***' 0.001 '**' 0.01 '*' 0.05 '.' 0.1 ' ' 1
##
##             exp(coef) exp(-coef) lower .95 upper .95
## x[set2]TRUE     0.468       2.14     0.214      1.02
##
## Concordance= 0.574  (se = 0.035 )
## Rsquare= 0.036   (max possible= 0.944 )
## Likelihood ratio test= 3.23  on 1 df,   p=0.0724
## Wald test            = 3.62  on 1 df,   p=0.0571
## Score (logrank) test = 3.8  on 1 df,   p=0.0513

summary(coxph(trait$S_10year[set2,]~x[set2]+trait$node[set2]+trait$size[set2]))

## Call:
## coxph(formula = trait$S_10year[set2, ] ~ x[set2] + trait$node[set2] +
##     trait$size[set2])
##
##   n= 88, number of events= 30
##    (1 observation deleted due to missingness)
##
##                     coef exp(coef) se(coef)     z Pr(>|z|)
## x[set2]TRUE      -0.736     0.479    0.401 -1.84    0.066 .
## trait$node[set2] -0.367     0.693    0.394 -0.93    0.351
## trait$size[set2]  0.853     2.347    0.362  2.36    0.018 *
## ---
## Signif. codes:
## 0 '***' 0.001 '**' 0.01 '*' 0.05 '.' 0.1 ' ' 1
##
##                  exp(coef) exp(-coef) lower .95 upper .95
## x[set2]TRUE          0.479      2.088     0.218      1.05
## trait$node[set2]     0.693      1.444     0.320      1.50
## trait$size[set2]     2.347      0.426     1.155      4.77
##
## Concordance= 0.656  (se = 0.053 )
## Rsquare= 0.098   (max possible= 0.944 )
## Likelihood ratio test= 9.09  on 3 df,   p=0.0282
## Wald test            = 8.85  on 3 df,   p=0.0313
## Score (logrank) test = 9.33  on 3 df,   p=0.0252

i=3
print('Calabro')
```

```
## [1] "Calabro"
```

```
x<- mat[,i]>Th[i-1]
set2 <- Site[[1]]
summary(coxph(trait$S_10year[set2,]~x[set2]))
```

```
## Call:
## coxph(formula = trait$S_10year[set2, ] ~ x[set2])
##
##   n= 88, number of events= 30
##    (1 observation deleted due to missingness)
##
##                 coef exp(coef) se(coef)     z Pr(>|z|)
## x[set2]TRUE -1.380     0.252    0.368 -3.75  0.00018 ***
## ---
## Signif. codes:
## 0 '***' 0.001 '**' 0.01 '*' 0.05 '.' 0.1 ' ' 1
##
##             exp(coef) exp(-coef) lower .95 upper .95
## x[set2]TRUE     0.252       3.97     0.122     0.518
##
## Concordance= 0.66  (se = 0.037 )
## Rsquare= 0.135   (max possible= 0.944 )
## Likelihood ratio test= 12.7  on 1 df,   p=0.000358
## Wald test            = 14.1  on 1 df,   p=0.000178
## Score (logrank) test = 16.4  on 1 df,   p=5.23e-05
```

```
summary(coxph(trait$S_10year[set2,]~x[set2]+trait$node[set2]+trait$size[set2]))
```

```
## Call:
## coxph(formula = trait$S_10year[set2, ] ~ x[set2] + trait$node[set2] +
##     trait$size[set2])
##
##   n= 88, number of events= 30
##    (1 observation deleted due to missingness)
##
##                      coef exp(coef) se(coef)     z Pr(>|z|)
## x[set2]TRUE      -1.309     0.270    0.368 -3.55  0.00038
## trait$node[set2] -0.294     0.745    0.389 -0.76  0.44934
## trait$size[set2]  0.816     2.261    0.380  2.14  0.03201
##
## x[set2]TRUE      ***
## trait$node[set2]
## trait$size[set2] *
## ---
## Signif. codes:
## 0 '***' 0.001 '**' 0.01 '*' 0.05 '.' 0.1 ' ' 1
##
##                  exp(coef) exp(-coef) lower .95 upper .95
## x[set2]TRUE          0.270      3.702     0.131     0.556
## trait$node[set2]     0.745      1.342     0.348     1.597
## trait$size[set2]     2.261      0.442     1.073     4.764
##
## Concordance= 0.703  (se = 0.053 )
```

```
## Rsquare= 0.181   (max possible= 0.944 )
## Likelihood ratio test= 17.6  on 3 df,   p=0.00053
## Wald test            = 17.8  on 3 df,   p=0.000483
## Score (logrank) test = 20.7  on 3 df,   p=0.000123
```

By modifying the code above, one can swap the discovery with the validation cohort and reproduce our results in the paper.

2.4 Robustness of Cox model

We estimate the univariate hazard ratio for ITLR on DSS using a Cox proportional hazards model using all samples.

```
ITL<-mat[,1]>th_ITL
summary(coxph(trait$S_10year ~ ITL))

## Call:
## coxph(formula = trait$S_10year ~ ITL)
##
##   n= 179, number of events= 46
##    (2 observations deleted due to missingness)
##
##            coef exp(coef) se(coef)     z Pr(>|z|)
## ITLTRUE -1.137     0.321    0.307 -3.71  0.00021 ***
## ---
## Signif. codes:
## 0 '***' 0.001 '**' 0.01 '*' 0.05 '.' 0.1 ' ' 1
##
##         exp(coef) exp(-coef) lower .95 upper .95
## ITLTRUE     0.321       3.12     0.176     0.585
##
## Concordance= 0.621  (se = 0.028 )
## Rsquare= 0.065   (max possible= 0.916 )
## Likelihood ratio test= 12  on 1 df,   p=0.000532
## Wald test            = 13.8  on 1 df,   p=0.000209
## Score (logrank) test = 15.3  on 1 df,   p=9.28e-05
```

The hazard ratio for ITLR is 0.25 (lower 95% 0.13, higher lower 95% 0.49). We also check the robustness of our results using bootstrap analysis. We sampled the data with replacement 1,000 times and repeated the log-rank survival analysis:

```
set.seed(45)  ## seed set to make the result reproducible
n <- length(ITL)
resB1 <- replicate(1000,1-pchisq(
         survdiff(trait$S_10year ~ ITL,
         subset=sample(1:sum(n), replace=TRUE)
         )$chisq,1))
mean(resB1 < 0.05)

## [1] 0.932
```

This means in 93% our results of univariate analysis stay significant in the perturbed data.

```r
set.seed(45)
resB2 <- replicate(1000, summary(coxph(trait$S_10year ~ ITL+trait$node+trait$size,
    subset=sample(1:sum(n), replace=TRUE)))$coef[1,5])
mean(resB2 < 0.05)

## [1] 0.947
```

This means in 95% our results of multivariate analysis stay significant. These demonstrated the stability of ITLR as a prognostic marker and prognostic factor in TN breast cancers.

3 Generation of ITLR data step-by-step

We now go back to the process in which the ITL measure was generated.

3.1 Image data

CRImage processes a H&E slide by first dividing it into 2,000 pixels by 2,000 pixels sub-images and identifying cells in these sub-images. Therefore the cell locations for these sub-images need to be combined. We provide combined cell identifies and spatial locations for all 181 TNBC whole-section H&E sections as R data files in the 'CellPosAndMask' folder. These files are named by their image ID. Each file contain the x, y and class columns storing x y coordinates as well as the class of each cell in the large H&E slide. There is also a 'mask' binary matrix to denote the tissue area. The resolution of this image is 5um per pixel. Note that some sub images are missing in the output data if the processing failed due to large amount of artefacts or few tissue.

3.2 Identify the optimal bandwidth for computing cancer density

By sampling 10 random samples, we compute the Mean Square Error over a range of different bandwidths h for computing cancer density.

```
library(splancs)
MSE <- NULL
set.seed(10)
ffs <- sample(dir('./data/CellPosAndMask/'), 10)
for (ff in ffs){
  res <- try(load(paste('./data/CellPosAndMask/', ff,  sep='')))
  CellPos[,1] <- as.character(CellPos[,1])
  CellPos[,2] <- as.numeric(CellPos[,2])
  CellPos[,3] <- as.numeric(CellPos[,3])
  CellPos <- CellPos[rowSums(is.na(CellPos))==0, ]
  CellPos[,3] <- ncol(Mask) - CellPos[,3] +1
  CellPos[,3][ CellPos[,3] > ncol(Mask)] <- ncol(Mask)
  CellPos <- CellPos[CellPos[,1]!='a',]
  cell.c <- data.frame(x=as.numeric(CellPos[CellPos[,1]=='c',2]), y=as.numeric(CellPos[CellPos
  cv <- mse2d(as.points(cell.c), poly=cbind(c(0, 0, nrow(Mask), nrow(Mask)), c(0, ncol(Mask),
  MSE <- rbind(MSE, cv$mse)
}
save(cv, MSE, file='./data/BandwidthSelection.rdata')
```

```
load(file='./data/BandwidthSelection.rdata')
x <- cv$h
y <- apply(MSE, 2, mean)
z <- apply(MSE, 2, sd)
par(mfrow=c(2,1), mar=c(3,3,1,1))
plot(x, y, pch=19, ylab='MSE',  xlab='Bandwidth')
abline(v=3)
plot(x[x>3], z[x>3], pch=19, ylab='Standard Deviation', xlab='Bandwidth')
abline(v=5)
```

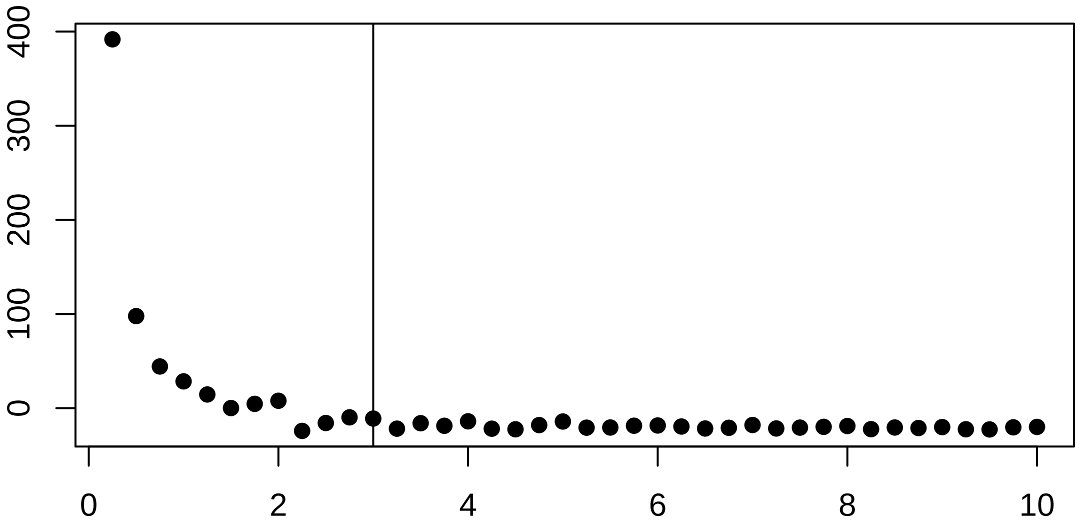

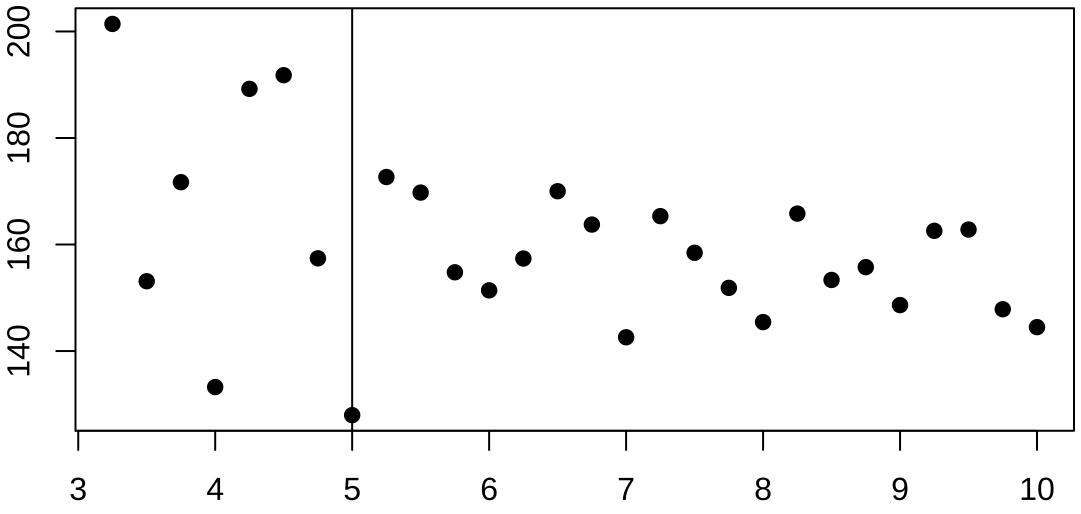

From the top plot we can see that median MSE of 10 samples is small for h>3 (vertical line). And variability of MSE is at the minimum at h=5. Thus we choose h=5 as the optimal bandwidth (vertical line).

3.3 Generate spatial proximity to cancer for each lymphocyte

Now, we can generate spatial scores given the cell position data using the following getITL function. getITL function uses the cell position files to infer a cancer density map using the bandwidth selected above.

```
getITL <- function(ff, h=5, ...){
  require(EBImage)
  require(splancs)

  res <- try(load(paste('./data/CellPosAndMask/', ff, '.rdata', sep='')))
  if (class(res)!='try-error'){
    CellPos[,1] <- as.character(CellPos[,1])
    CellPos[,2] <- as.numeric(CellPos[,2])
    CellPos[,3] <- as.numeric(CellPos[,3])
    CellPos <- CellPos[rowSums(is.na(CellPos))==0, ]
    CellPos[,3] <- ncol(Mask) - CellPos[,3] +1
    CellPos[,3][ CellPos[,3] > ncol(Mask)] <- ncol(Mask)
```

```
    cell.c <- data.frame(x=as.numeric(CellPos[CellPos[,1]=='c',2]), y=as.numeric(CellPos[CellP
    res <- kernel2d(as.points(cell.c), poly=cbind(c(0, 0, nrow(Mask), nrow(Mask)), c(0, ncol(M
    cell.l <- data.frame(x=as.numeric(CellPos[CellPos[,1]=='l',2]), y=as.numeric(CellPos[CellP
    z.l <- unlist(sapply(1:length(cell.l$x), function(x)  res$z[cell.l$x[x], cell.l$y[x]]))
  }
  z.l
}
```

Using this function, we can then generate measurements for each lymphocyte for each tumour.

```
itl <- list()
files <- trait$file
for (ff in files)
  itl <- c(itl, list(try(getITL(ff, h=5, w=3, cex=.5, ifPlot=F))))
names(itl) <- files
save(itl, file='./data/ITL.rdata')
```

By default getITL function uses the cut-offs of 0.03662728 and 0.10507473 to determine intra-tumour (ITL), adjacent to tumour (ATL), and distal-to-tumour lymphocytes (DTL). We will now describe how these cut-offs were selected.

3.4 Identify sub-populations of lymphocyte by unsupervised learning

We use the Gaussian mixture clustering and BIC implemented in the R package mclust for the discovery of lymphocyte sub-populations. 100,000 lymphocytes were randomly sampled from the itl object and then clustered.

```
library(mclust)

## Package 'mclust' version 4.4
## Type 'citation("mclust")' for citing this R package in publications.

load(file='./data/ITL.rdata')
set.seed(11)
x <- sample(as.numeric(unlist(itl)),100000)
res <- Mclust(x, G=1:5)
print(res)

## 'Mclust' model object:
##  best model: univariate, unequal variance (V) with 3 components
```

```
plot(hist(x,breaks=500, plot=FALSE),col="grey",border="grey",freq=FALSE,
     xlab="Lymphocyte proximity to cancer",main="")
lines(density(x),lty=2)
plot.normal.components.mclust(x, res)
```

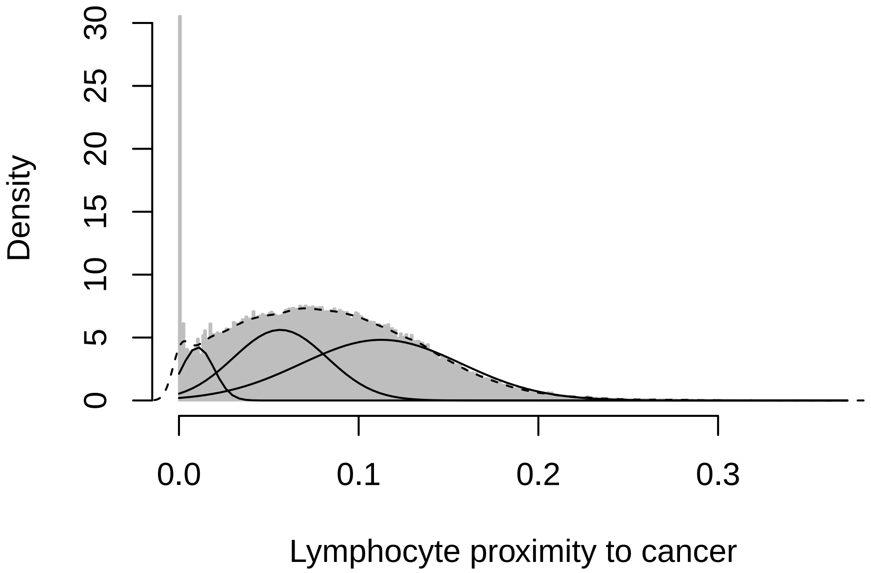

3.5 Clustering stability

We repeated the sampling process to generate clusters 200 times, and evaluated output from Mclust and mclustBIC.

```
BIC <- list()
TH <- list()
G <- NULL
Means <- NULL
SDs <- NULL
for (i in 1:200){
        set.seed(i)
        x <- sample(as.numeric(unlist(itl)),100000)
        res <- Mclust(x, G=1:5)
        th <- sapply(1:2, function(y) max(x[res$classification==y]))
        BIC <- c(BIC, list(res$BIC))
        Means <- rbind(Means, sapply(1:3, function(y) mean(x[res$classification==y], na.rm=T))
        SDs <- rbind(SDs, sapply(1:3, function(y) sd(x[res$classification==y], na.rm=T)))
        TH <- c(TH, th)
        G <- c(G, res$G)
}
TH <- matrix(unlist(TH), ncol=2, byrow=T)
save(TH, BIC, G, Means, file="./data/ITLclusterstability.rdata")
```

BIC values for these 200 runs are plotted.

```
load("./data/ITLclusterstability.rdata")
plot(y=pmax(BIC[[1]][,1], BIC[[1]][,2], na.rm=T), x=1:5, ylim=range(unlist(BIC), na.rm=T), yla
for (i in 1:200)
lines(y=pmax(BIC[[i]][,1], BIC[[i]][,2], na.rm=T), x=1:5, col="grey")
```

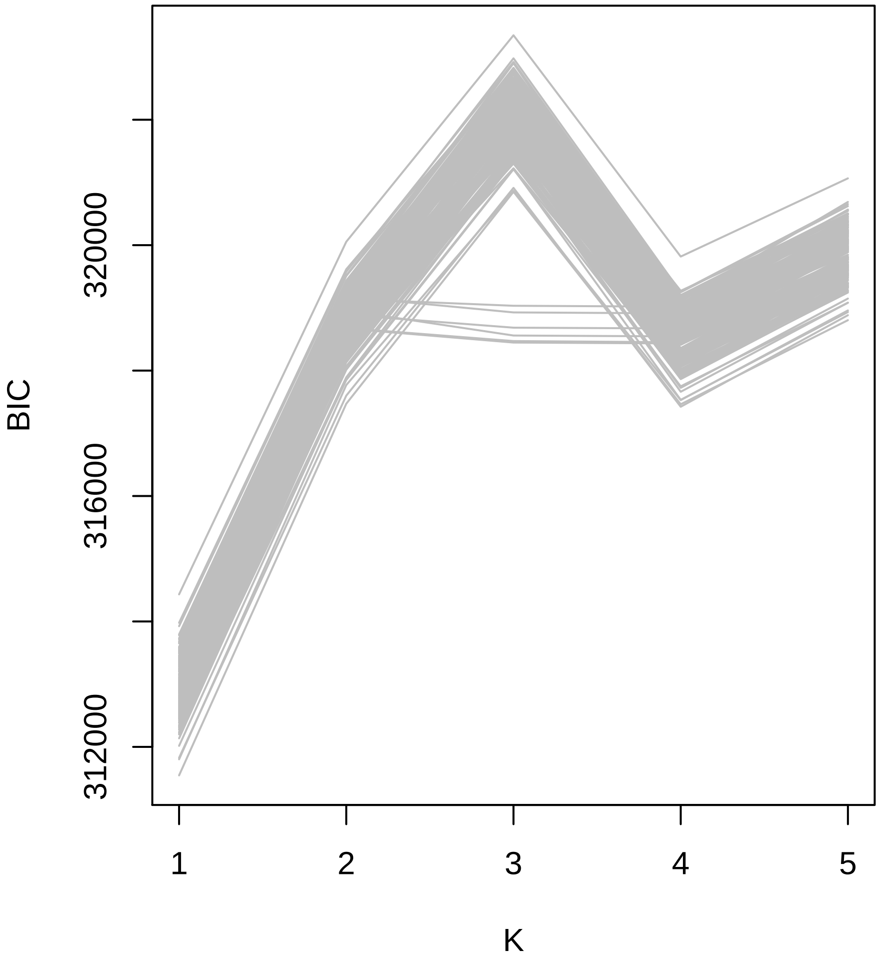


```
table(G)/200
```

The three-cluster solution k=3 remains optimal in 97% of the time, and k=5 was chosen 3% of the times. The median of cluster means when there are three clusters are 0.0114, 0.0603 and 0.1322 with standard deviation 0.002 and 0.0047 and 0.0045 , respectively.

```
par(mfrow=c(1,2))
hist(G, main="Distribution", xlab="Optimal k")
boxplot(Means[G==3,], ylab="Mean", xlab="Cluster")
```

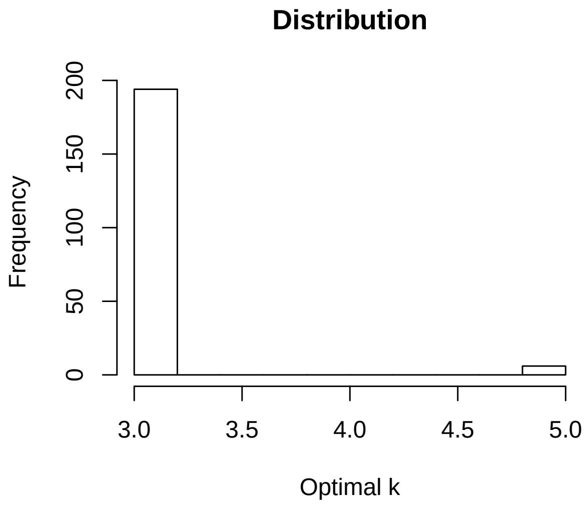

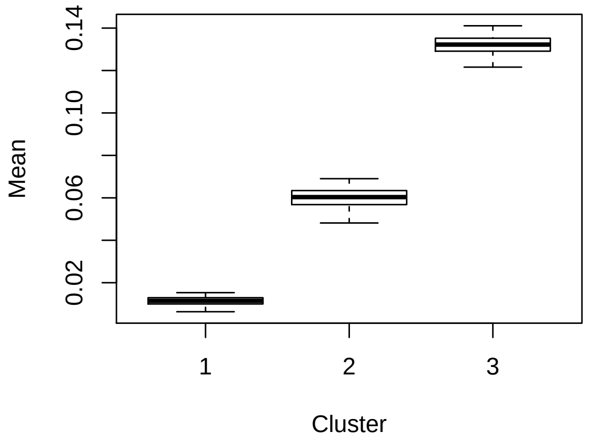

Therefore, the clustering result of lymphocytes from randomly sampled data is stable. Since the clustering is stable, we took cut-offs at the maximum value of the first and second clusters from one of the sampling runs as our cut-offs for determining lymphocyte classification for the remaining samples.

4 Differences of the three lymphocyte clusters

Here, we examine what each of the lymphocyte clusters entails.

4.1 Distance to nearest cancer cell

We first used a sample with relatively low number of cells as large distance matrix will need to be constructed. First, we calculate the distance from a lymphocyte to the nearest cancer cell. Load the cell position data for this sample. The distance matrix between lymphocytes to all cancer cells was computed using the vectorized_pdist function instead of the dist function to speed up the process. It generates squared euclidean distances.

```
ff <- '16571.rdata'
res <- try(load(paste('./data/CellPosAndMask/', ff, sep='')))
fn <- strsplit(ff, split='.', fixed=T)[[1]][1]
th <- c(0.03662728, 0.10507473)
labels <- sapply(itl[fn][[1]], function(x) if (x >=th[2]){3}else{if (x<th[1]){1}else{2}})
d <- vectorized_pdist(as.matrix(CellPos[CellPos[,1]=='l',2:3]), as.matrix(CellPos[CellPos[,1]=
d.min <- sqrt(apply(d, 1, min))

par(mfrow=c(3,1))
hist(d.min[labels==1]*5, xlim=range(d.min*5), col="#8291F7", xlab="Distance (micron)", br=100,
hist(d.min[labels==2]*5, xlim=range(d.min*5), col="blue", xlab="Distance (micron)", br=20, mai
hist(d.min[labels==3]*5, xlim=range(d.min*5), col="black", xlab="Distance (micron)", br=10, ma
```

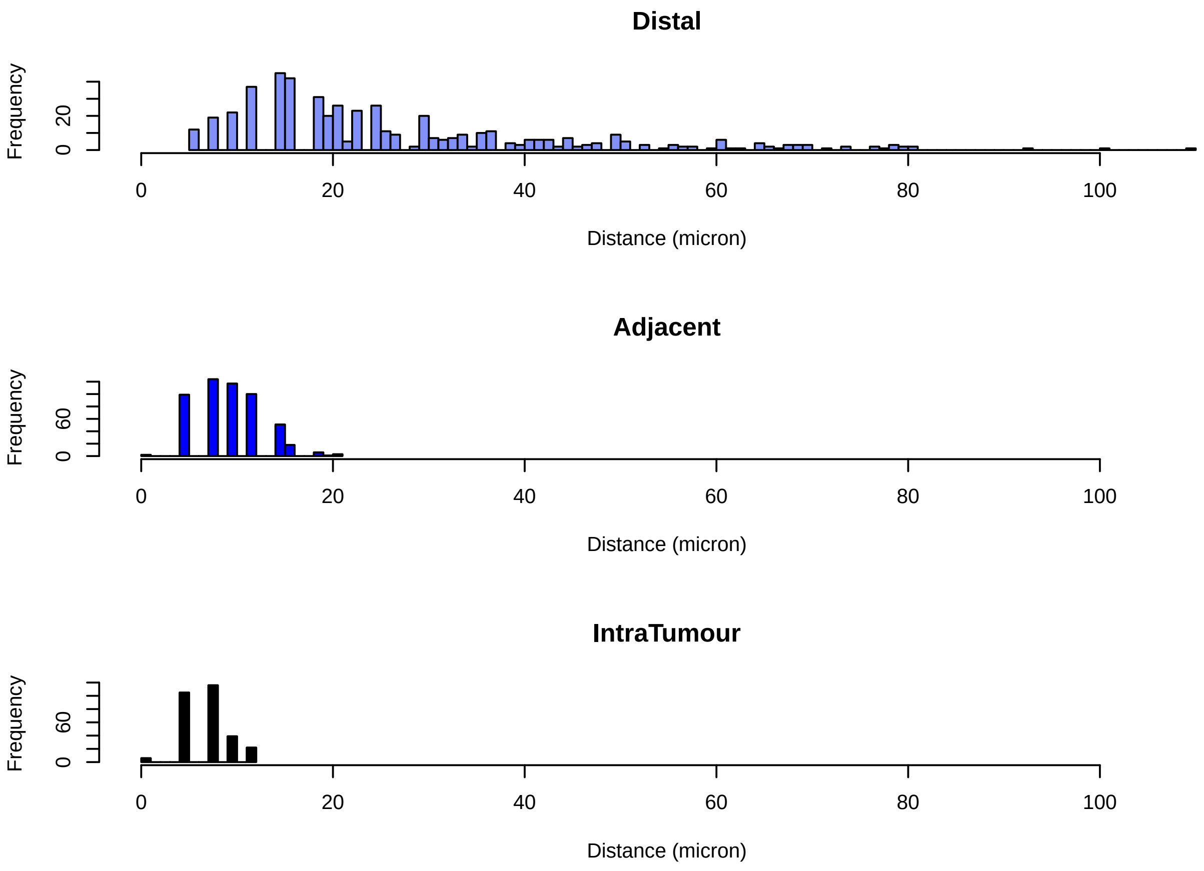


```r
mean(d.min[labels==3])

## [1] 1.376

sd(d.min[labels==3])

## [1] 0.4489

mean(d.min[labels==2])

## [1] 1.869

sd(d.min[labels==2])

## [1] 0.6839
```

Here, d.min is the minimal distances to cancer cell for all ITLs in this sample. The distribution of d.min show that the mean distance is 1.37 (sd 0.44) units, each unit being 5um. The length of a breast cancer nuclear is in the order of 5-10um, so an ITL would be no more than one cancer nuclear away from cancer cells.

We observed an overlap between classes in terms of their minimal distances to cancer cells. For example, some Adjacent-tumour lymphocytes are also positioned very close to cancer cells.

4.2 Distance to cancer convex hull

We hypothesised that the spatial arrangement of cancer cells surrounding these two classes of lymphocytes might be different. To test this hypothesis, we examined the way how cancer cells near the lymphocytes are arranged. For 9 randomly selected ITLs (red), we can plot the 5 nearest cancer cells

(black). We can measure the way a lymphocyte is surrounded by cancer cells by drawing a polygon of the cancer cells and examine how is the lymphocyte positioned from the centroid of cancer cell polygon (green cross). We used means of the cell positions to estimate centroids. If nearest cancer cells are all to one side of a lymphocyte, the lymphocyte is likely to be positioned far away from the centroid.

```r
par(mfrow=c(3,3))
for (j in 1:9){
i <- which(labels==3)[j]
y <- d[i,] <= sort(d[i,])[5]
hullpt <- chull(CellPos[CellPos[,1]=='c',][y,2:3])
centroid <- colMeans(CellPos[CellPos[,1]=='c',][y,2:3][hullpt,])
m <- rbind(CellPos[CellPos[,1]=='l',][i, 2:3], centroid)
plot(rbind(CellPos[CellPos[,1]=='c',][y,2:3], m), col=c(rep(1, 5), 2,3), pch=c(rep(19, 6), 3))
segments(m[1,1], m[1,2], m[2,1], m[2,2])
}
```

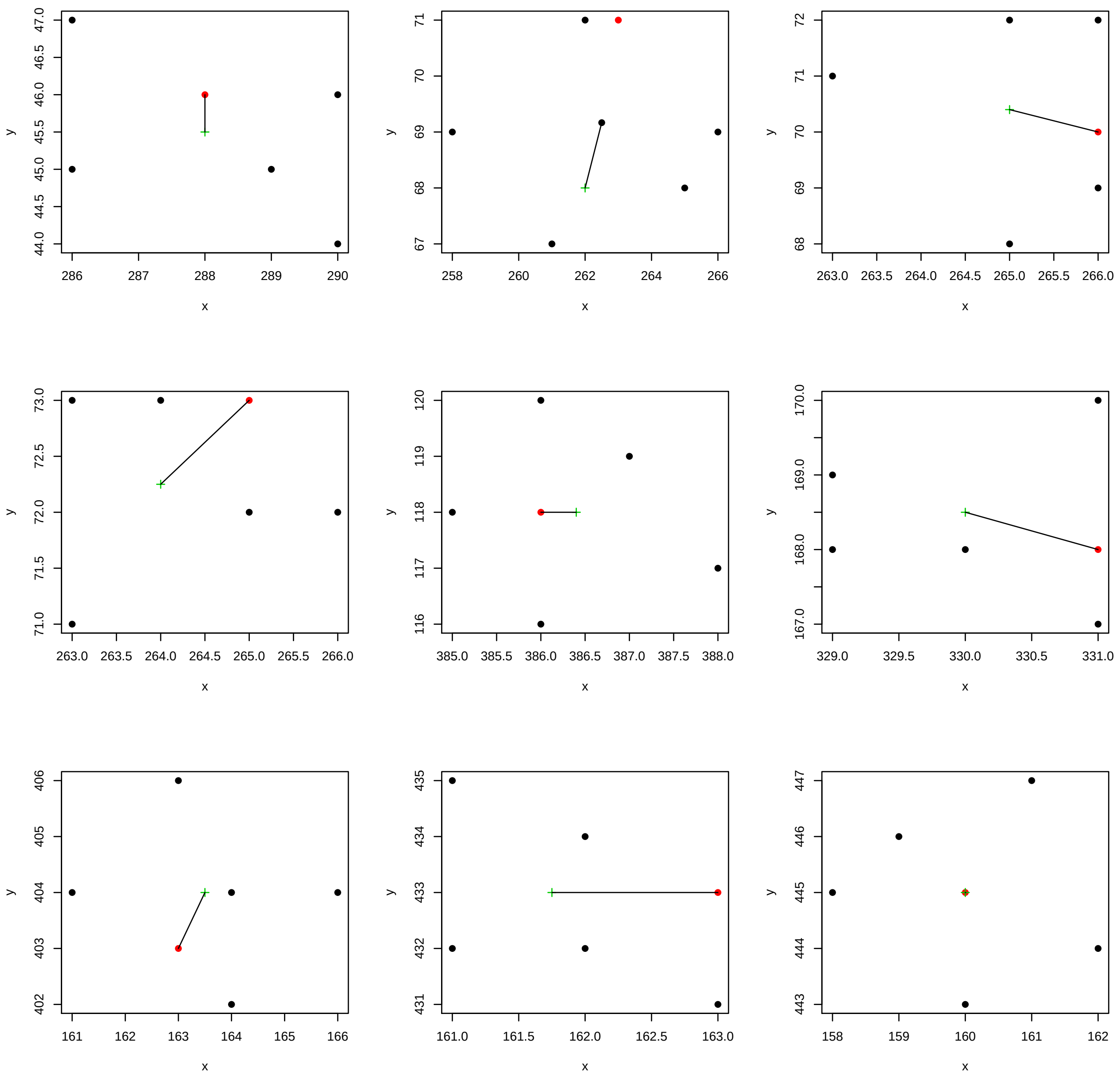

Similarly for adjacent-tumour lymphocytes:

```r
par(mfrow=c(3,3))
for (j in 1:9){
i <- which(labels==2)[j]
y <- d[i,] <= sort(d[i,])[5]
hullpt <- chull(CellPos[CellPos[,1]=='c',][y,2:3])
centroid <- colMeans(CellPos[CellPos[,1]=='c',][y,2:3][hullpt,])
m <- rbind(CellPos[CellPos[,1]=='l',][i, 2:3], centroid)
plot(rbind(CellPos[CellPos[,1]=='c',][y,2:3], m), col=c(rep(1, 5), 2,3), pch=c(rep(19, 6), 3))
segments(m[1,1], m[1,2], m[2,1], m[2,2])
}
```

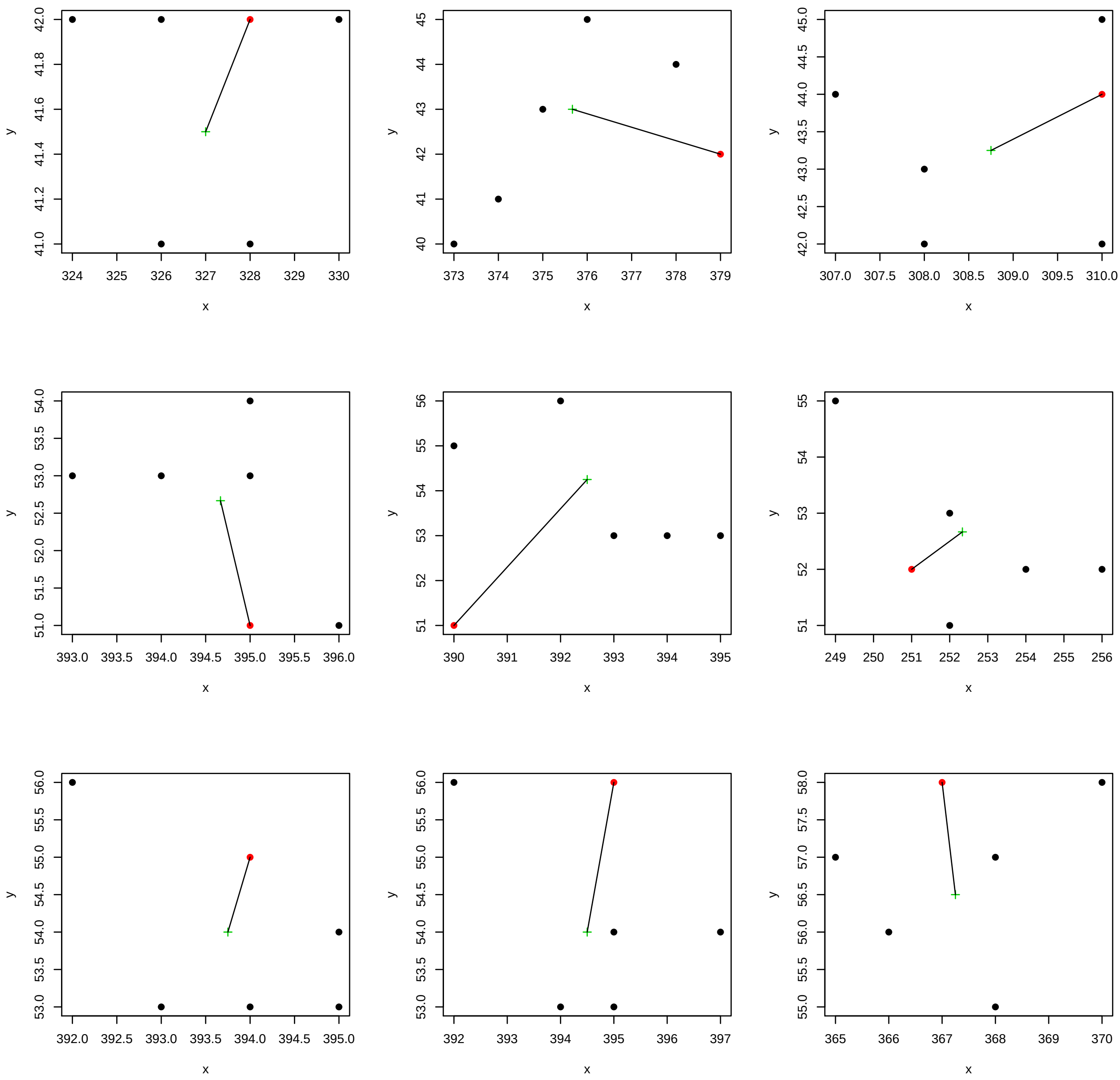

Thus for every lymphocyte, its distance to the centroid of convex hull formed by 5 nearest cancer cells is computed and plotted against minimal distances.

```r
d.centroid <- sapply(1:nrow(d), function(i) {
                y <- d[i,] <= sort(d[i,])[5]
                hullpt <- chull(CellPos[CellPos[,1]=='c',][y,2:3])
                centroid <- colMeans(CellPos[CellPos[,1]=='c',][y,2:3][hullpt,
```

```
                                m <- rbind(CellPos[CellPos[,1]=='l',][i, 2:3], centroid)
                                dist(m)[1]
                })


par(mfrow=c(2,2))
boxplot(d.min~labels, ylab='Distance to the nearest cancer cell', main=fn, pch=19, cex=.3, yli
boxplot(d.centroid~labels, ylab='Distance to centroid of nearest cancer cells', main=fn, pch=1
plot(d.min, d.centroid, pch=labels+16, col=replace.vector(labels, 1:3, c("#8291F7", "blue", "b
plot(d.min, d.centroid, pch=labels+16,  col=replace.vector(labels, 1:3, c("#8291F7", "blue", "
```

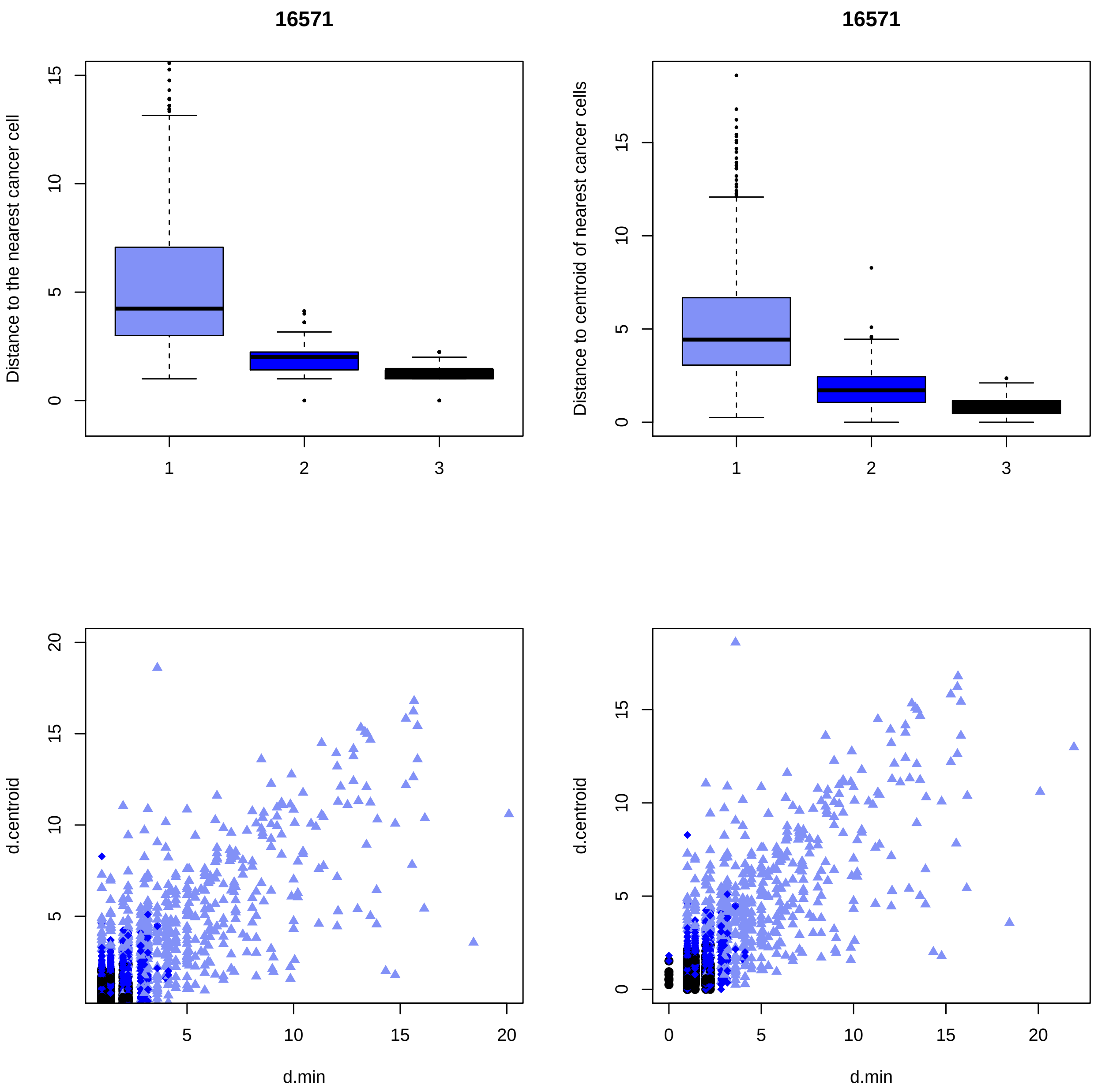

These plots illustrate the differences between ITLs and ATLs in terms of spatial arrangement of surrounding cancer cells, which now can be quantitatively measured. The differences between different classes of lymphocytes are significant, as showed by t test.

4.3 Differences in 10,000 randomly sampled lymphocytes

Now we can test this in a number of samples. 10,000 lymphocytes were sampled from 20 samples for 500 each. d.min and d.centroid were computed as described above.

```
set.seed(11)
ffs <- sample(dir('./data/CellPosAndMask/'), 35)
D.min <- NULL
D.centroid <- NULL
D.itl <- NULL
Labels <- NULL
for (ff in ffs){
        res <- try(load(paste("./data/CellPosAndMask/", ff, sep='')))
        if (sum(CellPos[,1]=='c') < 100000){
                fn <- strsplit(ff, split='.', fixed=T)[[1]][1]
                labels <- sapply(itl[fn][[1]], function(x) if (x >=th[2]){3}else{if (x<th[1]){
                idx <- sample(1:sum(CellPos[,1]=='l'), 500, replace=TRUE)
                labels <- labels[idx]
                D.itl <- c(D.itl, itl[fn][[1]][idx])

                d <- vectorized_pdist(as.matrix(CellPos[CellPos[,1]=='l', ][idx,2:3]), as.matr
                d.min <- apply(d, 1, min)
                d.min <- sqrt(d.min)

                d.centroid <- sapply(1:nrow(d), function(i) {
                                y <- d[i,] <= sort(d[i,])[5]
                                hullpt <- chull(CellPos[CellPos[,1]=='c',][y,2:3])
                                centroid <- colMeans(CellPos[CellPos[,1]=='c',][y,2:3][hullpt,
                                m <- rbind(CellPos[CellPos[,1]=='l',][idx,][i, 2:3], centroid)
                                dist(m)[1]
                })

                pdf(paste("./figure/", fn, ".pdf"), width=11, height=11)
                par(mfrow=c(2,2))
                boxplot(d.centroid~labels, ylab='Distance to centroid of nearest cancer cells'
                boxplot(d.min~labels, ylab='Distance to the nearest cancer cell', main=fn, pch
                plot(d.min, d.centroid, pch=labels+16, cex=.43, col=replace.vector(labels, 1:3
                plot(d.min, d.centroid, pch=labels+16, cex=.43, col=replace.vector(labels, 1:3
                dev.off()

                D.min <- c(D.min, d.min)
                D.centroid <- c(D.centroid, d.centroid)
                Labels <- c(Labels, labels)
}}
D.centroid <- D.centroid*5
D.min <- D.min*5
save(D.min, D.centroid, Labels, D.itl, file="./data/RandomSampleSpatialCancerArrangement.rdata
```

The measures are then mapped to micron as unit.

```
load("./data/RandomSampleSpatialCancerArrangement.rdata")

idx <- sample(1:length(D.min), 1000, replace=FALSE)
idx<- idx[D.min[idx]< 50 & D.centroid[idx] <50]
```

```
par(mfrow=c(3,2))
boxplot(D.min~Labels, ylab='Distance to the nearest cancer cell', ylim=c(-1, 60), pch=19, cex=
boxplot(D.centroid~Labels, ylab='Distance to centroid of nearest cancer cells', ylim=c(-1, 60)
plot(D.min[idx], D.centroid[idx], pch=Labels[idx]+16,  col=replace.vector(Labels[idx], 1:3, c(
plot(D.min[idx], D.centroid[idx], pch=Labels[idx]+16,col=replace.vector(Labels[idx], 1:3, c("#
plot(D.itl[idx], D.centroid[idx], pch=Labels[idx]+16,col=replace.vector(Labels[idx], 1:3, c("#
plot(D.itl[idx], D.min[idx], pch=Labels[idx]+16,col=replace.vector(Labels[idx], 1:3, c("#8291F
```

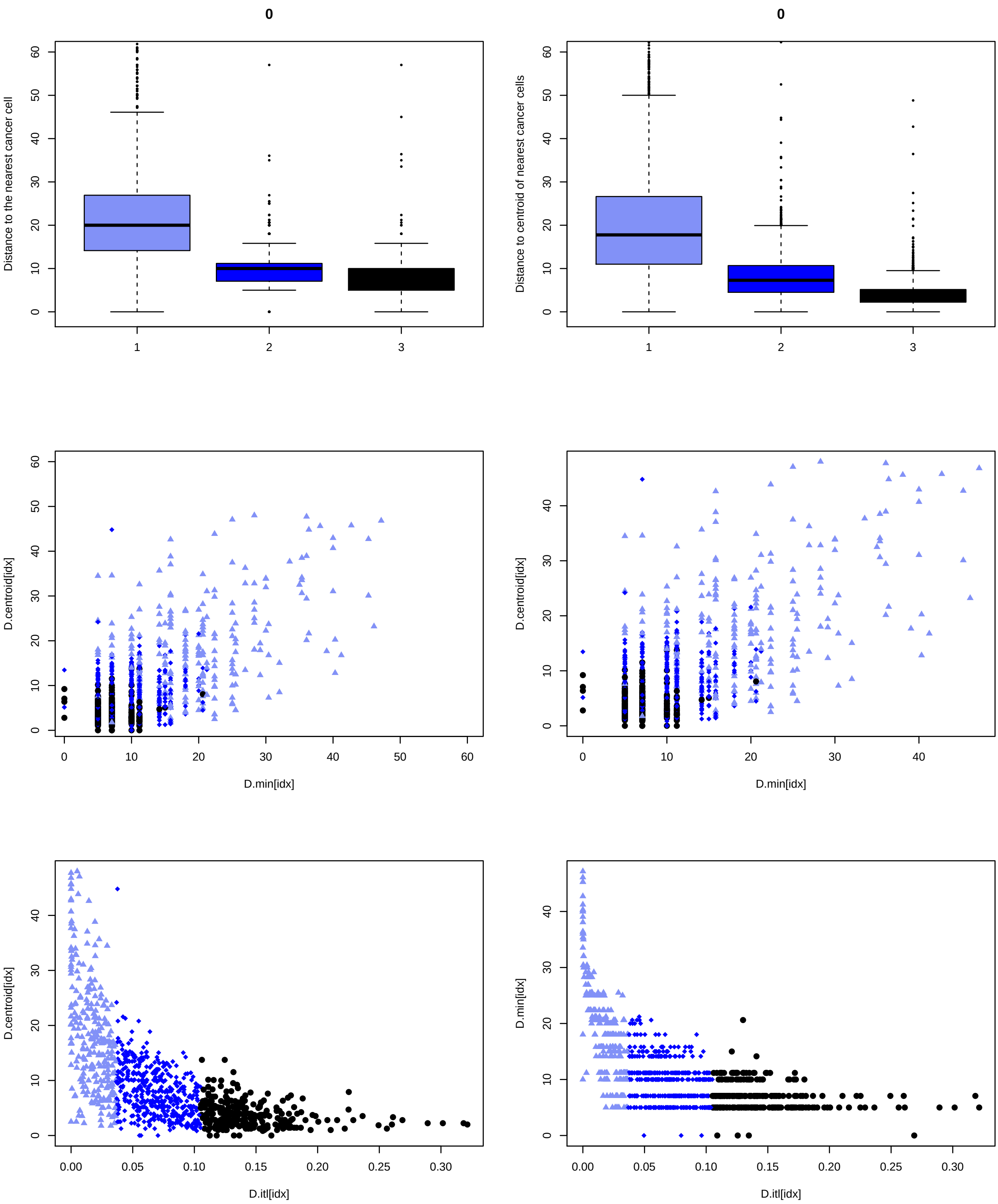

Thus the kernel-based spatial measure scales non-linearly to both minimal distance and distance to centroid.

The median, sd and interquantile range values of distance measures for ITLs, ATLs and DTLs:

```
IQR3 <- function(x, ...) quantile(x, 0.75, ...)
IQR1 <- function(x, ...) quantile(x, 0.25, ...)
sapply(1:3, function(x) median(D.min[Labels==x], na.rm=T))

## [1] 20.000 10.000  7.071

sapply(1:3, function(x) sd (D.min[Labels==x], na.rm=T))

## [1] 26.326  3.743  3.157

sapply(1:3, function(x) IQR1(D.min[Labels==x], na.rm=T))

##    25%    25%    25%
## 14.142  7.071  5.000

sapply(1:3, function(x) IQR3(D.min[Labels==x], na.rm=T))

##   75%   75%   75%
## 26.93 11.18 10.00
```

Similarity for D.centroid, and we can test the differences with t.test:

```
sapply(1:3, function(x) median (D.centroid[Labels==x], na.rm=T))

## [1] 17.766  7.289  3.606

sapply(1:3, function(x) sd (D.centroid[Labels==x], na.rm=T))

## [1] 27.261  4.751  3.095

sapply(1:3, function(x) IQR1(D.centroid[Labels==x], na.rm=T))

##    25%    25%    25%
## 11.000  4.507  2.236

sapply(1:3, function(x) IQR3(D.centroid[Labels==x], na.rm=T))

##    75%    75%    75%
## 26.627 10.680  5.154

t.test(D.centroid[Labels==1], D.centroid[Labels==2])$p.value

## [1] 7.482e-149

t.test(D.centroid[Labels==2], D.centroid[Labels==3])$p.value

## [1] 0

t.test(D.min[Labels==1], D.min[Labels==2])$p.value

## [1] 1.334e-150

t.test(D.min[Labels==2], D.min[Labels==3])$p.value

## [1] 1.226e-213
```

One could inspect all samples to see if they all have a statistically significant differences among the lymphocyte clusters in terms of the spatial arrangement of surrounding cancer cells.

```
Pvals <- NULL
for (ff in dir('./data/CellPosAndMask/')){
res <- try(load(paste('./data/CellPosAndMask/', ff, sep='')))
fn <- strsplit(ff, split='.', fixed=T)[[1]][1]
labels <- sapply(itl[fn][[1]], function(x) if (x >=th[2]){3}else{if (x<th[1]){1}else{2}})
if (nrow(CellPos)>10000){
        CellPos <- CellPos[1:10000,]
        labels <- labels[1:sum(CellPos[,1]=='l')]
        }
        d <- vectorized_pdist(as.matrix(CellPos[CellPos[,1]=='l',2:3]), as.matrix(CellPos[Cell
        d.centroid <- sapply(1:nrow(d), function(i) {
                                y <- d[i,] <= sort(d[i,])[5]
                                hullpt <- chull(CellPos[CellPos[,1]=='c',][y,2:3])
                                centroid <- colMeans(CellPos[CellPos[,1]=='c',][y,2:3][hullpt,
                                m <- rbind(CellPos[CellPos[,1]=='l',][idx,][i, 2:3], centroid)
                                dist(m)[1]
                })
        Pvals <- c(Pvals,try( t.test(d.centroid[labels==2], d.centroid[labels==3])$p.value))
}
```

5 Generating ITLR in TNBCs

5.1 Illustration of ITLs with a sample slide image

Using one of the images (19069) as an example, we plot the H&E and the corresponding cancer density map with identified lymphocytes.

```
res <- load(paste('./data/CellPosAndMask/19069.rdata', sep=''))
th=c(0.03662728, 0.10507473)
h=5
dim(CellPos)

## [1] 198694      3
```

There are 198694 cells in this image.

```
    CellPos[,1] <- as.character(CellPos[,1])
    CellPos[,2] <- as.numeric(CellPos[,2])
    CellPos[,3] <- as.numeric(CellPos[,3])
    CellPos <- CellPos[rowSums(is.na(CellPos))==0, ]
    CellPos[,3] <- ncol(Mask) - CellPos[,3] +1
    CellPos[,3][ CellPos[,3] > ncol(Mask)] <- ncol(Mask)

    library(splancs)
    cell.c <- data.frame(x=as.numeric(CellPos[CellPos[,1]=='c',2]), y=as.numeric(CellPos[CellP
    res <- kernel2d(as.points(cell.c), poly=cbind(c(0, 0, nrow(Mask), nrow(Mask)), c(0, ncol(M
    cell.l <- data.frame(x=as.numeric(CellPos[CellPos[,1]=='l',2]), y=as.numeric(CellPos[CellP
    z.l <- unlist(sapply(1:length(cell.l$x), function(x)  res$z[cell.l$x[x], cell.l$y[x]]))

    png('./figure/ITLmap.png', width=2000, height=ncol(Mask)*2000/nrow(Mask))
```

```
    par(mar=c(0,0,0,0))
    res$z[Mask[,ncol(Mask):1]==0] <- NA
    image(res, add=F, xaxt='n', yaxt='n', ylim=c(0,ncol(Mask)), xlim=c(0,nrow(Mask)),  col=col
    points(as.points(cell.l[z.l<th[1],]), pch=19, col="#8291F7", cex=.1)
    points(as.points(cell.l[(z.l>=th[1])&(z.l<th[2]),]), pch=19, col='blue', cex=.1)
    points(as.points(cell.l[z.l>=th[2],]), pch=19, col='black', cex=.1)
    segments(x0=83, y0=20, x1=122, y1=20, lwd=4)
    text(x=100, y=50, "200um", cex=3)
    contour(res, add=TRUE, levels=th, col="black")
    legend('topleft', inset=.01, cex=2, legend=c('Intra-tumour lymphocyte', 'Adjacent-to-tumou
    dev.off()
```

```
tmp=hist(z.l,br=100, border='white', xlab='Lymphocyte proximity to cancer')
hist(z.l[z.l<th[1]], br=tmp$breaks, col="#8291F7", add=T)
hist(z.l[(z.l>=th[1])&(z.l<th[2])], br=tmp$breaks, col="blue", add=T)
hist(z.l[z.l>=th[2]], br=tmp$breaks, col="black", add=T)
```

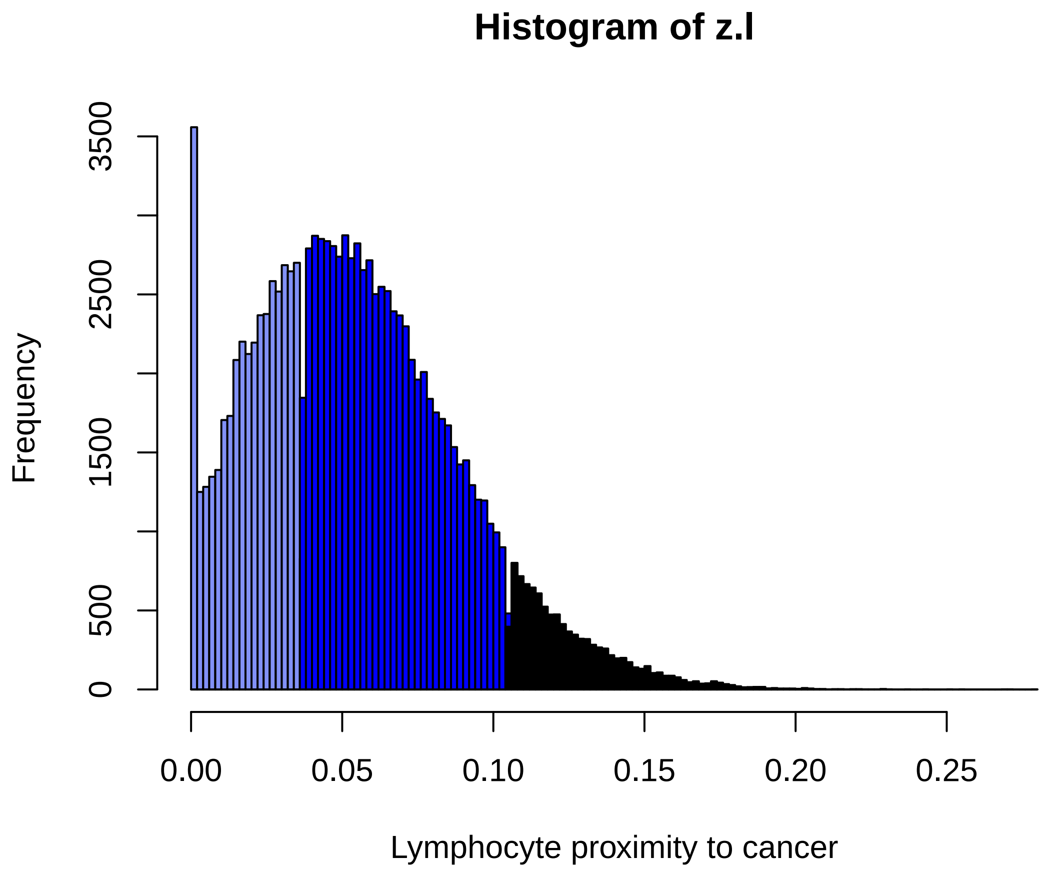


```
cell.o <- data.frame(x=as.numeric(CellPos[CellPos[,1]=='o',2]), y=as.numeric(CellPos[CellPos[,
res <- kernel2d(as.points(cell.o), poly=cbind(c(0, 0, nrow(Mask), nrow(Mask)), c(0, ncol(Mask)

## Xrange is  0 2801
## Yrange is  0 1480
## Doing quartic kernel

z.l_o <- unlist(sapply(1:length(cell.l$x), function(x)  res$z[cell.l$x[x], cell.l$y[x]]))
plot(z.l_o, z.l, pch=19, cex=.3, xlab='Lymphocyte to stroma', ylab='Lymphocyte to cancer')
```

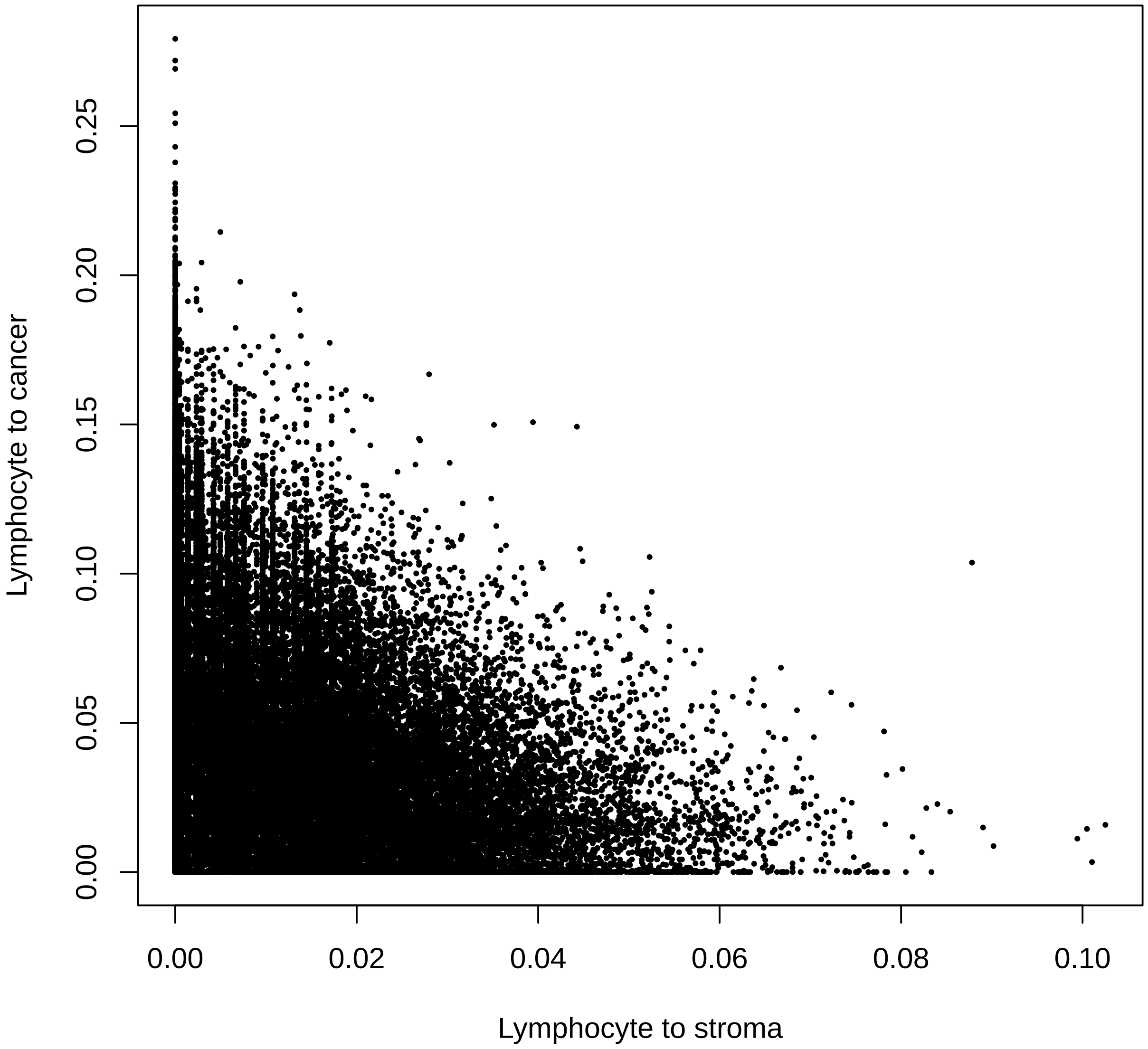

5.2 Generating ITLR, ATLR, and DTLR

Subsequently, the cut-offs can be used to classify every lymphocyte based on their data stored in the R object itl. mat.l is a matrix with columns of 'Distal', 'Adjacent', 'Intra' denoting the number of lymphocytes in each class for a tumour.

```
th=c(0.03662728, 0.10507473)
mat.l <- NULL
for (i in 1:length(itl)){
  z.l <- itl[[i]]
  cl <- rep(1,length(z.l))
  cl[z.l>th[1] & z.l<th[2]] <- 2
  cl[z.l>=th[2]] <- 3
  mat.l <- rbind(mat.l, c(sum(cl==1), sum(cl==2), sum(cl==3)))
}
colnames(mat.l) <- c('Distal', 'Adjacent', 'Intra')
rownames(mat.l) <- names(itl)
```

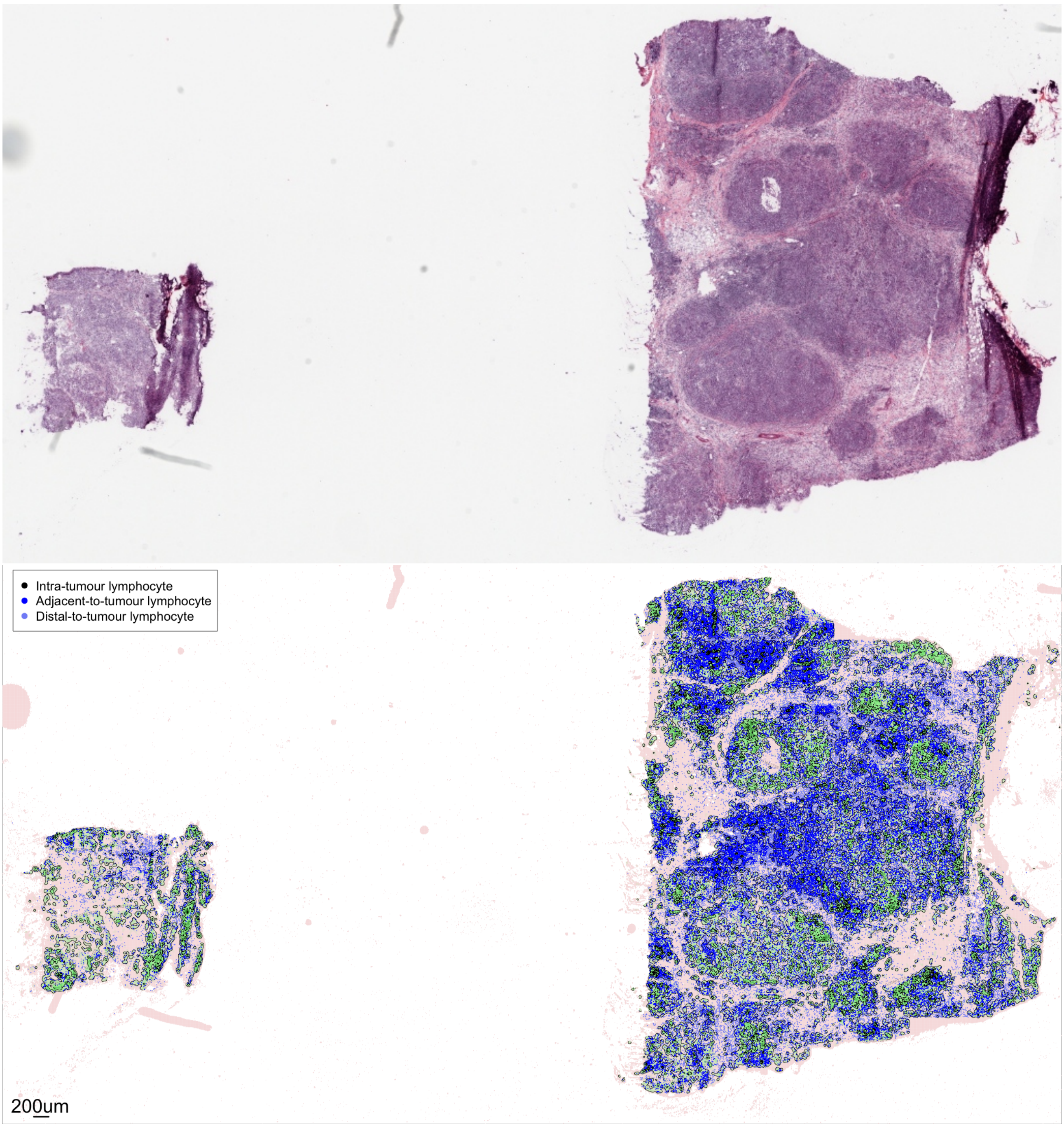
Intra-tumour lymphocyte
Adjacent-to-tumour lymphocyte
Distal-to-tumour lymphocyte
200um

The Intra column of mat.l is the number of intra-tumour lymphocytes. This divided by the number of cancer cells (trait$nTumour) is the ITLR measurement and should be identical to the column ITL of the R matrix mat used in the previous section:

```
x <- as.numeric(mat.l[,3]/trait$nTumour)
y <- as.numeric(mat[,1])
identical(x, y)

## [1] TRUE
```

6 Compare ITLR, ATLR, and DTLR

Using Site 1 as the discovery cohort and Site 2 as the validation cohort, we will test the association between prognosis and ATLR and DTLR. Again we search a range of quantiles from 20% to 80% for the optimal cut-off in the discovery cohort.

```
mat2 <- mat.l/trait$nTumour
s <- 1
Th <- NULL
par(mfrow=c(2,3))
for (i in 1:2){
p <- sapply(testrange, function(q){
  dat <- data.frame(x=mat2[Site[[s]],i]>quantile(mat2[Site[[s]],i], q), S=trait$S_10year[Site[
  fit <- survfit(S ~ x,data=dat)
  test <- survdiff(S ~ x, data=dat, rho=0)
  p.val <- 1 - pchisq(test$chisq, length(test$n) - 1)
  p.val})

plot(testrange, -log(p), pch=19, xlab='Quantile range', main=colnames(mat2)[i])
abline(h=-log(0.05), lty=2)
q <- testrange[which.min(p)]
th <- quantile(mat2[Site[[s]],i], q)
abline(v=q)
Th <- c(Th, th)

for (j in 1:2){
  tmp <- replace.vector(mat2[Site[[j]],i]>th, c(TRUE, FALSE), c('High', 'Low'))
  try( plotSurv(trait$S_10year[Site[[j]],], tmp, fileType='', name=colnames(mat2)[i]))
}
}
```

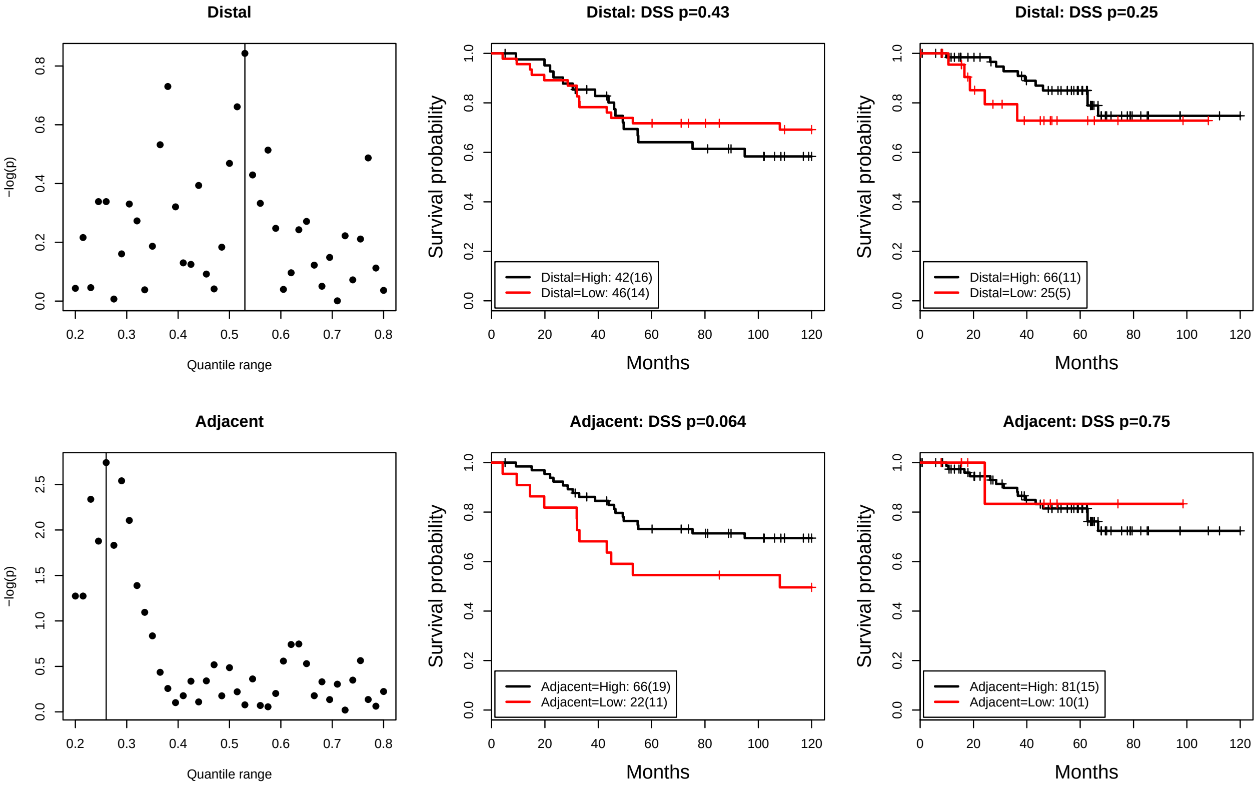

Test with Site 1 discovery cohort showed no significant differences in survival according to ATLR and DTLR. We then examined Site 2 and found the same.

```
s <- 2
Th <- NULL
par(mfrow=c(2,3))
for (i in 1:2){
p <- sapply(testrange, function(q){
  dat <- data.frame(x=mat2[Site[[s]],i]>quantile(mat2[Site[[s]],i], q), S=trait$S_10year[Site[
  fit <- survfit(S ~ x,data=dat)
  test <- survdiff(S ~ x, data=dat, rho=0)
  p.val <- 1 - pchisq(test$chisq, length(test$n) - 1)
  p.val})

plot(testrange, -log(p), pch=19, xlab='Quantile range', main=colnames(mat2)[i])
abline(h=-log(0.05), lty=2)
q <- testrange[which.min(p)]
th <- quantile(mat2[Site[[s]],i], q)
abline(v=q)
Th <- c(Th, th)

for (j in 1:2){
  tmp <- replace.vector(mat2[Site[[j]],i]>th, c(TRUE, FALSE), c('High', 'Low'))
  try( plotSurv(trait$S_10year[Site[[j]],], tmp, fileType='', name=colnames(mat2)[i]))
}
}
```

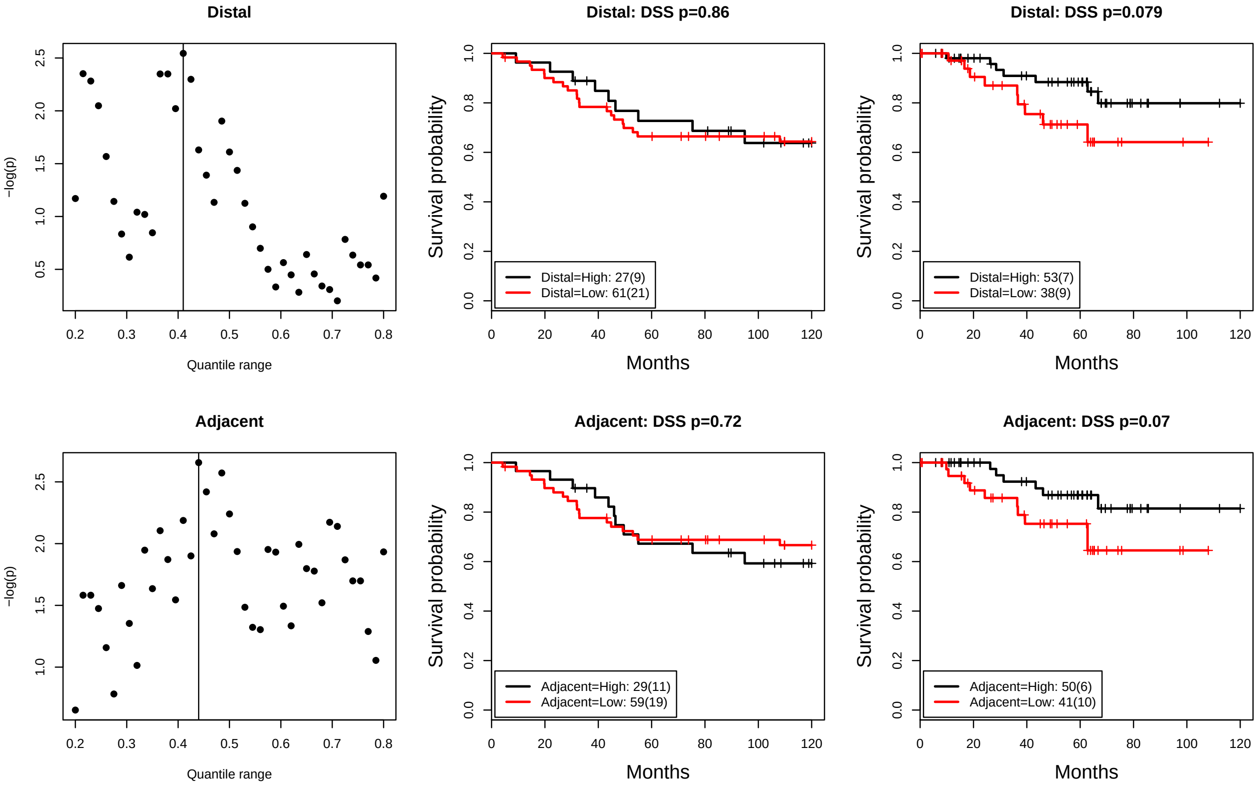

Distal
-log(p)
Quantile range
Distal: DSS p=0.86
Survival probability
Months
Distal=High: 27(9)
Distal=Low: 61(21)
Distal: DSS p=0.079
Distal=High: 53(7)
Distal=Low: 38(9)
Adjacent
Adjacent: DSS p=0.72
Adjacent=High: 29(11)
Adjacent=Low: 59(19)
Adjacent: DSS p=0.07
Adjacent=High: 50(6)
Adjacent=Low: 41(10)

7 Association with molecular profiling

Correlation analysis result between ITL and gene expression is provided as a matrix. This matrix contains probe IDs, symbol, genomic location, correlation value and q-value after FDR correction as well as gene description. To assess full gene expression data, please apply to the METABRIC consortium.

```
ITLtab <- read.csv('./data/cor.csv', as.is=T)
head(ITLtab)

##        ProbeId    Symbol Cytoband
## 1 ILMN_1780368     GPR18 13q32.3a
## 2 ILMN_1652650   SH3KBP1 Xp22.12b
## 3 ILMN_1691693     FCRL3  1q23.1d
## 4 ILMN_1759075 TNFRSF13B 17p11.2h
## 5 ILMN_1794740     CD151 11p15.5c
## 6 ILMN_1797428     FCRL3  1q23.1d
##              Genomic_location
## 1  chr13:98705370:98705419:-
## 2   chrX:19727685:19727734:-
## 3 chr1:155913076:155913125:-
## 4  chr17:16783174:16783223:-
## 5      chr11:828488:828537:+
## 6 chr1:155914667:155914716:-
##                                                 Description
## 1                              G protein-coupled receptor 18
## 2                        SH3-domain kinase binding protein 1
## 3                                        Fc receptor-like 3
## 4 tumor necrosis factor receptor superfamily, member 13B
## 5
## 6
##       cor        q
## 1  0.3928 0.001133
## 2  0.3982 0.001133
## 3  0.4017 0.001133
## 4  0.3926 0.001133
## 5 -0.3935 0.001133
## 6  0.4138 0.001133
```

8 Session Info

This document was prepared using R package knitr. Funciton knit2pdf("ITL.rnw") was used to compile the sweave file and generate the pdf file.

```
sessionInfo()

## R version 3.1.1 (2014-07-10)
## Platform: x86_64-apple-darwin13.1.0 (64-bit)
##
## locale:
## [1] en_GB.UTF-8/en_GB.UTF-8/en_GB.UTF-8/C/en_GB.UTF-8/en_GB.UTF-8
##
## attached base packages:
## [1] parallel  splines   stats     graphics  grDevices
## [6] utils     datasets  methods   base
##
## other attached packages:
## [1] splancs_2.01-34     sp_1.0-15
## [3] mclust_4.4          SAGx_1.38.0
## [5] multtest_2.20.0     Biobase_2.24.0
## [7] BiocGenerics_0.10.0 survival_2.37-7
## [9] knitr_1.6
##
## loaded via a namespace (and not attached):
##  [1] digest_0.6.4    evaluate_0.5.5  formatR_1.0
##  [4] grid_3.1.1      highr_0.3       lattice_0.20-29
##  [7] MASS_7.3-33     stats4_3.1.1    stringr_0.6.2
## [10] tools_3.1.1
```
